# Formation of Salts and Molecular Ionic Cocrystals of Fluoroquinolones and α,ω-Dicarboxylic Acids

**DOI:** 10.1021/acs.cgd.1c01509

**Published:** 2022-04-11

**Authors:** Ciaran O’Malley, Patrick McArdle, Andrea Erxleben

**Affiliations:** † School of Chemistry, National University of Ireland, Galway H91TK33, Ireland; ‡ Synthesis and Solid State Pharmaceutical Centre (SSPC), Limerick V94T9PX, Ireland

## Abstract

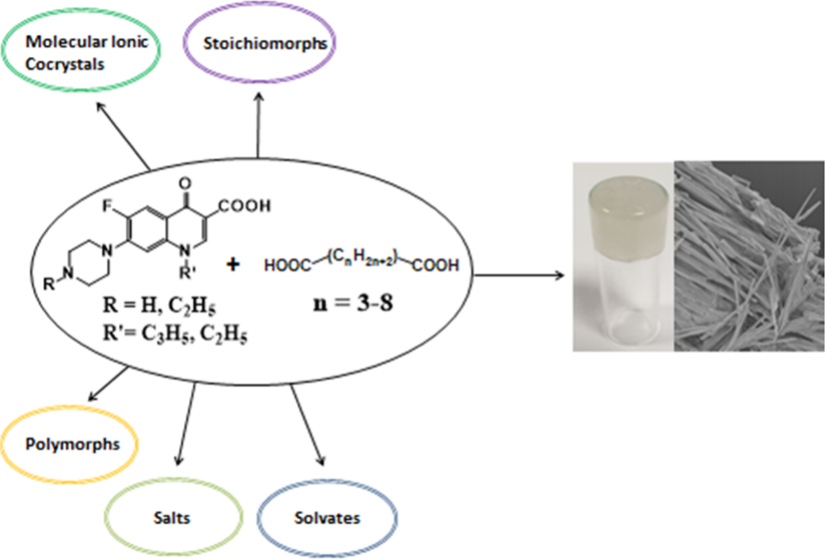

The cocrystallization of the fluoroquinolones ciprofloxacin (cip), norfloxacin (nor), and enrofloxacin (enro) with the α,ω-dicarboxylic acids glutaric acid (glu), adipic acid (adi), pimelic acid (pim), suberic acid (sub), azeliac acid (az), and sebacic acid (seb) resulted in 27 new molecular salts and ternary molecular ionic cocrystals of compositions A^+^B^–^, A_2_^+^B^2–^, A_2_^+^B^2–^B, and A^+^B^–^A. Depending on the solvent, different stoichiomorphs, solvates, or polymorphs were obtained. All salts and cocrystals contain the robust R_2_NH_2_^+...–^OOC or R_3_NH^+...–^OOC synthon but have different supramolecular ring motifs. Moderate solubility enhancements over the parent fluoroquinolones were observed. Salts in the ratio of 1:1 and 2:1 were also prepared by ball-milling. The milled sample nor/az (1:1) was shown to gel the GRAS (generally recognized as safe) solvent propylene glycol, and enro/sub (1:1) was shown to gel both propylene glycol and water. Dynamic rheology measurements confirmed that nor/az and enro/sub behave like viscoelastic materials and supramolecular gels.

## Abstract 2

The cocrystallization of the fluoroquinolones ciprofloxacin, norfloxacin, and enrofloxacin with saturated dicarboxylic acids HOOC-(CH_2_)_n_-COOH (*n* = 3−8) gave 27 new crystal structures of cocrystals, molecular salts, molecular ionic cocrystals, stoichiomorphs, solvates, and polymorphs, some of which are supramolecular gelators.

## Introduction

The fluoroquinolones ciprofloxacin (cip) and norfloxacin (nor) are broad-spectrum antibiotics that are widely prescribed to treat bacterial infections, in particular, infections of the respiratory and urinary tracts.^1,2^ Enrofloxacin (enro) is approved by the FDA for veterinary use. However, the therapeutic efficacy of these antibiotics is limited due to their low aqueous solubility and poor drug penetration.^3^ The poor solubility also impacts the development of liquid dosage forms, e.g., for parenteral or ophthalmic solutions.

**Figure 1:**
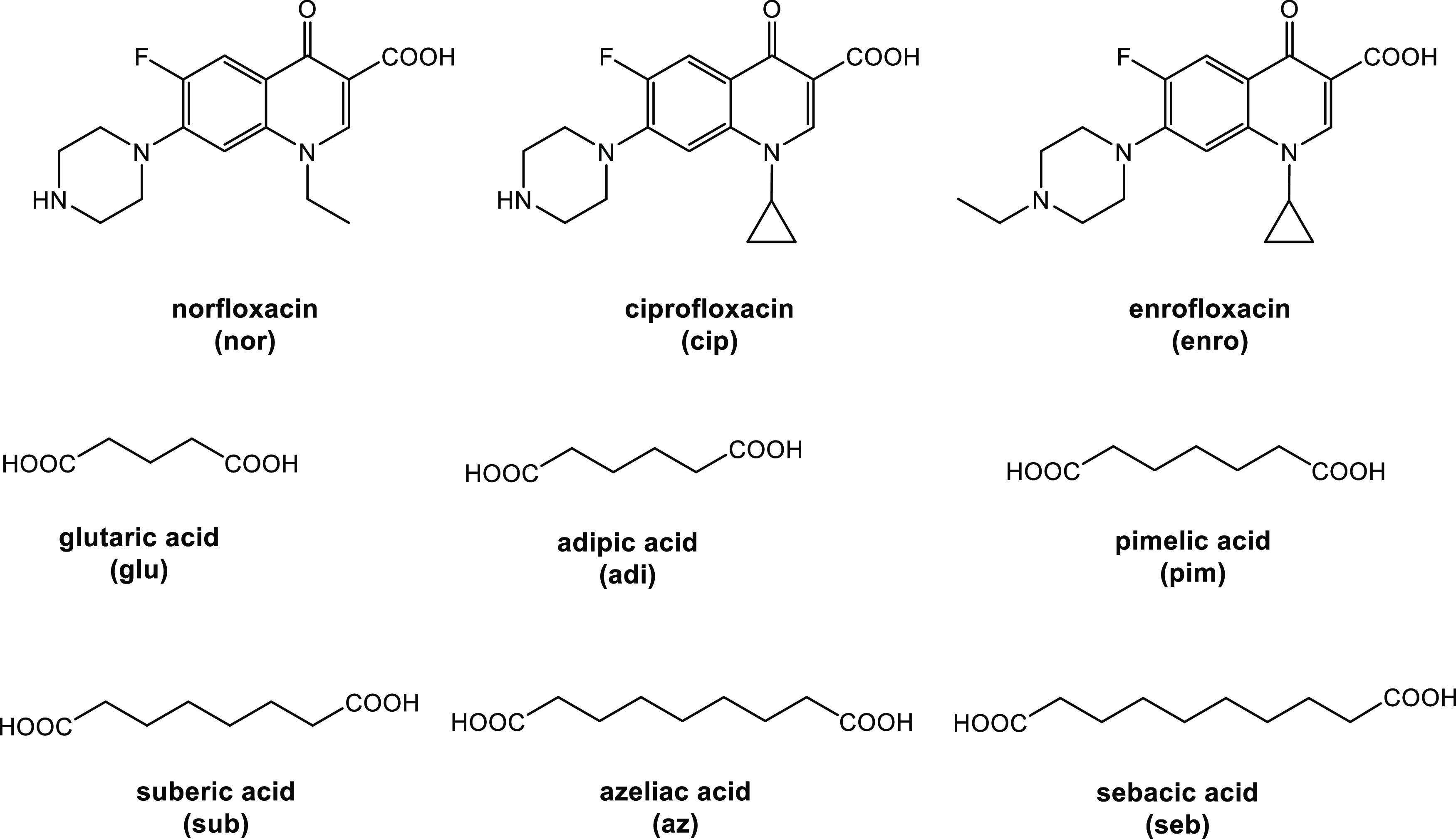
Structures of the fluoroquinolone antibiotics and dicarboxylic acids used in this study.

Nor, cip, and enro contain a basic piperazinyl nitrogen and a carboxylic acid group (Figure 1). The traditional approach to enhance the water solubility and thus the bioavailability of drug molecules with one or more ionizable groups is salt formation. Salts often achieve a 100- to 1000-fold increase in solubility over the respective neutral drug,^4^ and more than 50% of active pharmaceutical ingredients (APIs) are marketed as salts. A number of crystalline organic salts^5−17^ as well as amorphous polymeric salts^18,19^ of nor, cip, enro, and other fluoroquinolones have been investigated, and the crystal structures of various 1:1 and 2:1 salts with saccharin,^5^ monocarboxylic acid,^6−9^ dicarboxylic acid,^6,7,10,11^ and tricarboxylic acid coformers^12^ have been reported. (Nor^+^)_2_(ox^2–^)·4H_2_O, (nor^+^)(tar^–^)·2H_2_O, (cip^+^)(mal^–^)·2H_2_O, (cip^+^)(tar^–^), and (cip^+^)(cit^–^)·H_2_O, for example, gave enhanced intrinsic dissolution rates and solubilities at pH 6.8 (ox = oxalic acid, tar = tartaric acid, mal = malonic acid, cit = citric acid).^6^ (Enro^+^)(male^–^), (enro^+^)_2_(fum^2–^)·fum, and (enro^+^)_2_(suc^2–^)·suc, (enro^+^)_2_(ox^2–^)·6H_2_O (male = maleic acid, fum = fumaric acid, suc = succinic acid) were found to have a 30- to 110-fold higher solubility than enro.^7^ Gunman et al. studied the cocrystallization of pefloxacin with dicarboxylic acids and showed that the solubility of the succinate salt in phosphate buffer was 838-fold higher than that of pefloxacin.^13^ A small number of drug–drug salts of nor and cip with a sulfathiazole, diflunisal, and indoprofen counterion have also been reported.^9,14,15^

The cocrystallization of fluoroquinolones with carboxylic acids usually results in supramolecular structures containing the robust R_3_NH^+...–^OOCR heterosynthon between the protonated piperazine ring and the deprotonated carboxyl group of the coformer. Dastidar and co-workers have recently shown that ammonium monocarboxylate salts of the anticancer drug 5-fluorouracil acetic acid^20^ and of diflunisal,^21^ naproxen,^22^ and other nonsteroidal anti-inflammatory drugs^23,24^ can be low-molecular-weight gelators. Supramolecular gels of active pharmaceutical ingredients are of significant interest due to their potential application as self-drug-delivery (SDD) systems.^25^ Supramolecular gels are viscoelastic materials that form as a result of frustrated crystallization.^26^ The gelator molecules self-assemble through supramolecular interactions (H-bonding, π–π stacking, van der Waals interactions, halogen bonding) into a 1D polymeric structure with 1D H bonding being particularly important.^27−29^ This results in the formation of long fibers that can further entangle to give a self-assembled fibrillar network in which solvent molecules can be immobilized.^30^ Cocrystals and salts have several advantages over other multicomponent supramolecular gelators, such as the ease of synthesis and high, often quantitative yields.

Creating new organic molecular salts of fluoroquinolones and identifying crystal packing motifs are of interest from a crystal engineering point of view. Understanding the relationship between the crystal structure and properties such as solubility and gelation is fundamental to the design of new pharmaceutical materials. In this work, we have studied the crystal packing of molecular salts of nor, cip, and enro and α,ω-dicarboxylic acids HOOC-(CH_2_)_n_-COOH with long, flexible spacer chains (*n* = 3–8; Figure 1). Dicarboxylic acids with *n* = 2–10 are generally regarded as safe by the FDA. Azeliac acid (*n* = 7) has anti-inflammatory and antibacterial properties and is used for the treatment of acne and rosacea. While the nor and cip salts with adipic acid (*n* = 4),^16,17^ the enro salt with glutaric acid (*n* = 3),^7^ and the pefloxacin salts with glutaric, adipic, and suberic acids (*n* = 6)^13^ are already described in the literature, no systematic study on the effect of the spacer chain length has been carried out yet. Here, we report 27 new crystal structures of nor, cip, and enro salts of compositions A^+^B^–^ and A_2_^+^B^2–^ as well as molecular ionic cocrystals of compositions A_2_^+^B^2–^B and A^+^B^–^A. We compare their solubilities and also show that some of the salts behave as supramolecular gelators.

## Experimental Section

### Materials

Ciprofloxacin, norfloxacin, enrofloxacin, suberic acid, azeliac acid, and sebacic acid were obtained from Tokyo Chemical Industry (TCI, Europe). Glutaric acid, adipic acid, and pimelic acid were obtained from Sigma-Aldrich. The solvents methanol, acetonitrile (Merck Millipore), ethyl acetate (Sigma-Aldrich), acetone (Fisher Scientific), ethanol (Fisher Scientific), propylene glycol (TCI, Europe), and polyethylene glycol 400 (Sigma-Aldrich) were analytical grade and used as received.

### Preparation of the Salts

#### Solution Crystallization

Cip, nor, or enro (0.1 mmol) and a molar equivalent of the respective carboxylic acid coformer were dissolved in the minimum amount of solvent (water, methanol, ethanol, acetonitrile, acetonitrile/methanol, acetone, and ethyl acetate). The solutions were left in open vials to slowly evaporate at room temperature. Details of the crystallization experiments are provided in the Supporting Information (Tables S1–S3).

#### Ball-Milling

Bulk samples of the salts for the gelation experiments were prepared by liquid-assisted grinding. Room-temperature milling experiments were performed using an oscillatory ball mill (Mixer Mill MM400, Retsch GmbH & Co., Germany) with a 25 mL stainless steel milling jar containing one 15 mm diameter stainless steel ball. The fluoroquinolone and respective coformer were physically mixed in a 1:1 or 2:1 molar ratio (0.25 g sample total). Twenty-five microliters of methanol was added to the milling jar, and the samples were milled at 25 Hz for up to 60 min with a cool-down period of 15 min after 30 min of milling. The milled powder samples were analyzed immediately by X-ray powder diffraction.

### Thermal Analysis

Differential scanning calorimetry (DSC) and thermogravimetric analysis (TGA) were carried out with an STA625 thermal analyzer (RheometricScientific, Piscataway, New Jersey), calibrated using an indium standard. The temperature range was 20–300 °C, and the heating rate was 10 °C/min. Open aluminum crucibles were used, and nitrogen was purged in ambient mode.

### Solubility Study

Bulk samples were grown by crystallization from solution. All samples used in the solubility studies were gently ground to avoid any bias from large particles. The powder sample (100 mg) was placed in 3 mL of 0.1 M phosphate buffer (pH 6.8, 37 °C) and stirred at 300 rpm with an 8 mm magnetic stir bar for 48 h. The amount of dissolved fluoroquinolone was determined with a Varian Cary 50 SCAN UV/vis spectrophotometer (Santa Clara, CA). Cip, enro, and nor concentrations were measured at 270 nm. Standard solutions were prepared with phosphate buffer (0.1 M, pH 6.8). The resulting calibration curves were linear in the relevant concentration range. All solubility experiments were performed in triplicate.

### Gelation Experiments

Powder samples of the compounds for gelation experiments were prepared by ball-milling, as described above. To prepare a 10% w/v gel, 100 mg of the powdered sample was dissolved in 1 mL of the respective solvent in a standard 15 × 160 mm test tube. Samples were heated to within 10 °C of the respective solvent’s boiling point under stirring until dissolved. Samples were then allowed to cool slowly to room temperature.

### Rheology

Dynamic rheology measurements were performed on an Anton Paar modular compact rheometer (MCR 302) with a parallel plate setup with a 10 mm plate geometry. Experiments were performed at 37 °C.

### Determination of *T*_gel_

The sol–gel-dissociation temperature was determined by the dropping ball method. A glass ball (250 mg, 5 mm diameter) was placed on 1 mL of gel sample in a test tube (1.5 cm internal diameter). The test tube was then immersed in an oil bath, and the temperature was gradually increased by 1 °C/min until the ball touched the bottom of the test tube. The temperature at which the ball touched the bottom of the test tube was recorded as *T*_gel_.

### Scanning Electron Microscopy (SEM)

A thin layer of the dried gel was coated with a gold layer, and micrographs were captured on a Hitachi S2600N variable pressure scanning electron microscope. The experimental parameters were ×903 magnification, backscatter BSE resolution of 20 nm at 25, 5 kV accelerating voltage, 10 000 nA emission current, and 13.5 mm working distance.

### X-ray Powder Diffraction (XRPD)

X-ray powder patterns were recorded between 5 and 90° (2θ) on an Inel Equinox 3000 powder diffractometer (Artenay, France) using Cu Kα radiation (λ = 1.54178 Å, 35 kV, 25 mA). The diffractometer was fitted with a curved position-sensitive detector that was calibrated using Y_2_O_3_. The Oscail software package was used to calculate theoretical powder patterns from single-crystal data.^31^

### Crystal Structure Determination and Refinement

Single-crystal X-ray data were collected on an Oxford Diffraction Xcalibur system (Oxfordshire, U.K.) at room temperature or 150 K. The crystal structures were solved by direct methods using SHELXT and refined using SHELXL 2018/3 within the Oscail package.^31−33^ Crystallographic data and details of refinement are presented in Tables S4–S8. The cif files can be obtained free of charge at www.ccdc.cam.ac.uk/conts/retrieving.html or from the Cambridge Crystallographic Data Centre, Cambridge, U.K., with 2128064–2128092.

## Results and Discussion

Cocrystallization experiments of nor, cip, and enro with 1 equiv of glutaric acid (glu), adipic acid (adi), pimelic acid (pim), suberic acid (sub), azeliac acid (az), and sebacic acid (seb) were performed in water, methanol, ethanol, acetonitrile, acetonitrile/methanol, acetone, and ethyl acetate at room temperature. Crystals suitable for single-crystal X-ray analysis were obtained in all cases except from solutions containing cip and seb. Several of the salts crystallized as different stoichiomorphs, solvates, or polymorphs depending on the solvent. Our cocrystallization screen also yielded two new solvates of enro, namely, enro.CH_3_CN and enro.H_2_O (Figure S1).

### Single-Crystal X-ray Structures

**Figure 2:**
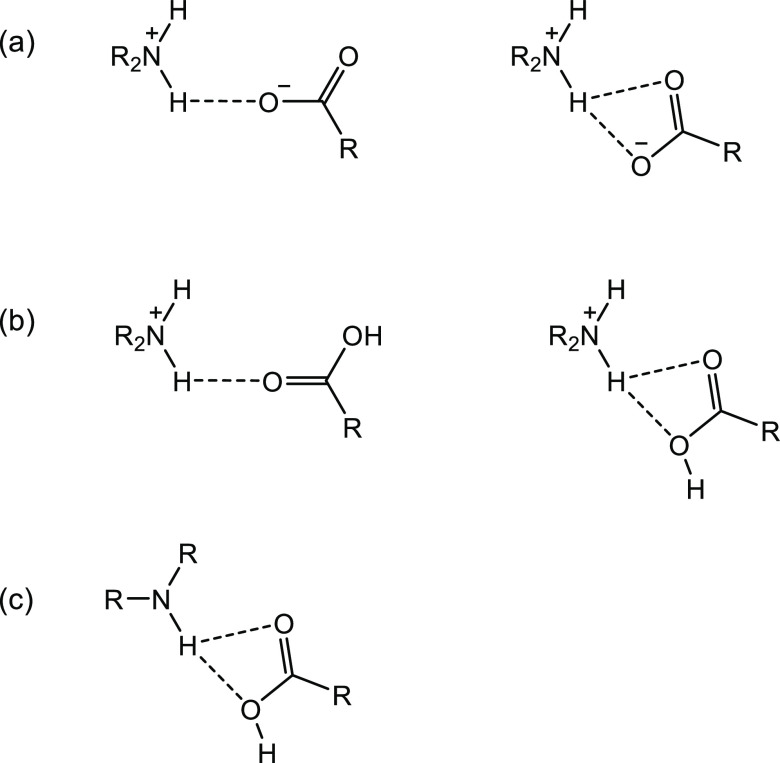
(a) R_2_NH_2_^+...–^OOC, (b) R_2_NH_2_^+...^O=C(OH)R, and (c) R_2_NH^...^O=C(OH)R synthons in the supramolecular salts of nor, cip, and enro described in this study.

X-ray analysis revealed salts of composition A^+^B^–^ containing the monodeprotonated acid anions [(cip^+^)(glu^–^), (enro^+^)(glu^–^)·0.33CH_3_CN·0.67H_2_O, (cip^+^)(pim^–^), (nor^+^)(pim^–^), (nor^+^)(pim^–^)·CH_3_OH, (enro^+^)(pim^–^)·H_2_O, (enro^+^)(pim^–^)·3H_2_O, (nor^+^)(sub^–^), (nor^+^)(sub^–^)·3H_2_O, (enro^+^)(sub^–^)·H_2_O, (enro^+^)(az^–^), (cip^+^)(az^–^)·CH_3_CN, and (enro^+^)(seb^–^)], salts of composition A_2_^+^B^2–^ containing the acid dianions [(nor^+^)_2_(glu^2–^)·H_2_O·CH_3_OH, (nor^+^)_2_(glu^2–^)·H_2_O·0.75CH_3_CN, (nor^+^)_2_(adi^2–^)·2H_2_O form I, (nor^+^)_2_(adi^2–^)·2H_2_O form II, (cip^+^)_2_(pim^2–^)·H_2_O, (nor^+^)_2_(pim^2–^)·C_2_H_5_OH, (enro^+^)_2_(pim^2–^)·1.5H_2_O, (cip^+^)_2_(sub^2–^)·4H_2_O, and (nor^+^)_2_(sub^2–^)·CH_3_OH, (nor^+^)_2_(seb^2–^)·4CH_3_OH], salts of composition A_2_^+^B^2–^B containing one acid dianion and one neutral acid molecule [(nor^+^)_2_(az^2–^)·az·4H_2_O, (enro^+^)_2_(adi^2–^)·adi·2CH_3_CN], and salts of composition A^+^B^–^A containing one cation, one acid monoanion, and one neutral fluoroquinolone molecule [(nor^+^)(seb^–^)·nor·H_2_O]. All structures show the expected R_2_NH_2_^+...–^OOC or R_3_NH^+...–^OOC synthon with the protonated piperazine nitrogen forming a single or a bifurcated H bond with the deprotonated carboxylate oxygen of the organic acid (Figure 2). In the A_2_^+^B^2–^B and A^+^B^–^A ionic cocrystals, the R_2_NH_2_^+...^O=C(OH)R/R_3_NH^+...^O=C(OH)R and R_2_NH^...^O=C(OH)R synthons are also observed. The carboxyl groups of the fluoroquinolone cations are less acidic than the dicarboxylic acids used (p*K*_a_ ∼ 6 vs 3.7–4.7). They are therefore protonated and form a strong intramolecular H bond to the neighboring carbonyl oxygen (S_1_^1^(6) motif). A table with H-bonding interactions in the supramolecular salts is provided in the Supporting Information (Table S9).

#### Salts of Composition A^+^B^–^

**Figure 3:**
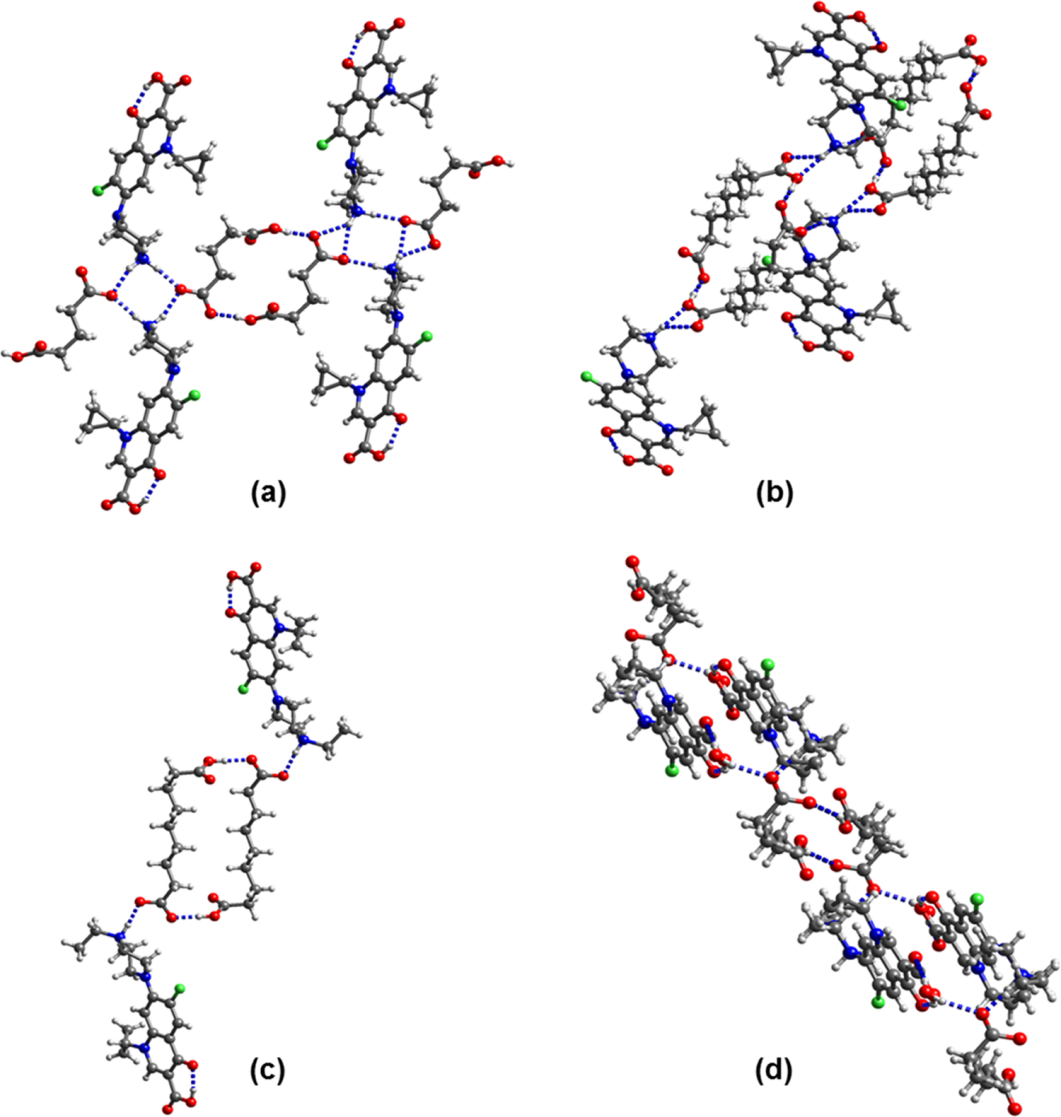
Hydrogen-bonding motifs in (a) (cip^+^)(glu^–^), (b) (cip^+^)(az^–^)·CH_3_CN, (c) (enro^+^)(az^–^), and (d) (enro^+^)(pim^–^)·H_2_O. Solvent molecules of crystallization in (cip^+^)(az^–^)·CH_3_CN is omitted for clarity.

In the 1:1 salts obtained in this study, the dicarboxylic acid monoanions form either infinite chains or cyclic dimers via COOH^...–^OOC H bonding. The only exception is (enro^+^)(pim^–^)·3H_2_O that contains neither of these two structural motifs. In (cip^+^)(glu^–^) (Figure 3a), (cip^+^)(az^–^)·CH_3_CN (Figure 3b), (enro^+^)(az^–^) (Figure 3c), (enro^+^)(pim^–^)·H_2_O (Figure 3d), and (enro^+^)(glu^–^)·0.33CH_3_CN·0.67H_2_O (Figure S2), the glu^–^, az^–^ and pim^–^ anions build up R_2_^2^(16), R_2_^2^(24) and R_2_^2^(20) rings. The COOH group is in the syn conformation in (cip^+^)(glu^–^) (τ = 5.8 and −10.4° for the two crystallographically independent glu^–^ in the asymmetric unit), (enro^+^)(pim^–^)·H_2_O (τ = −14.2°), (enro^+^)(az^–^) (τ = 1.3°), and (enro^+^)(glu^–^)·0.33CH_3_CN·0.67H_2_O (τ = 11.0, −2.4 and −7.3° for the three crystallographically independent glu^–^ in the asymmetric unit) and in the anti-conformation in (cip^+^)(az^–^)·CH_3_CN (τ = −178.3°). In (cip^+^)(glu^–^), H bonding between the protonated amino groups of two cip^+^ cations and the deprotonated carboxyl groups of two pim^–^ anions generates R_4_^2^(8) rings, while R_6_^6^(16) rings of two amino groups, two COO^–^ groups, and two COOH groups are present in (cip^+^)(az^–^)·CH_3_CN.

The hydrate (enro^+^)(pim^–^)·H_2_O contains an R_8_^8^(20) motif. The water of crystallization forms H bonds with the OH group of pim^–^, with the COO^–^ group of pim^–^ and with another water molecule of crystallization. Overall, four water molecules, two pim-OH groups and two pim-COO^–^ groups, are linked into a 20-membered ring. The latter also accepts a H bond from the protonated amino group of enro^+^. Furthermore, there is H bonding between the water of crystallization and the carboxyl group of the enro^+^ cation. By contrast, two enro^+^ cations interact with the COO^–^ groups of the cyclic az^–^ or glu^–^ dimers in (enro^+^)(az^–^) and (enro^+^)(glu^–^)·0.33H_3_CN·0.67H_2_O resulting in discrete enro^...^acid dimer^...^enro entities in these two salts. The crystal packing of the latter is built up by interleaved stacks of the z-shaped enro^...^(glu)_2..._enro units (Figure S2).

**Figure 4:**
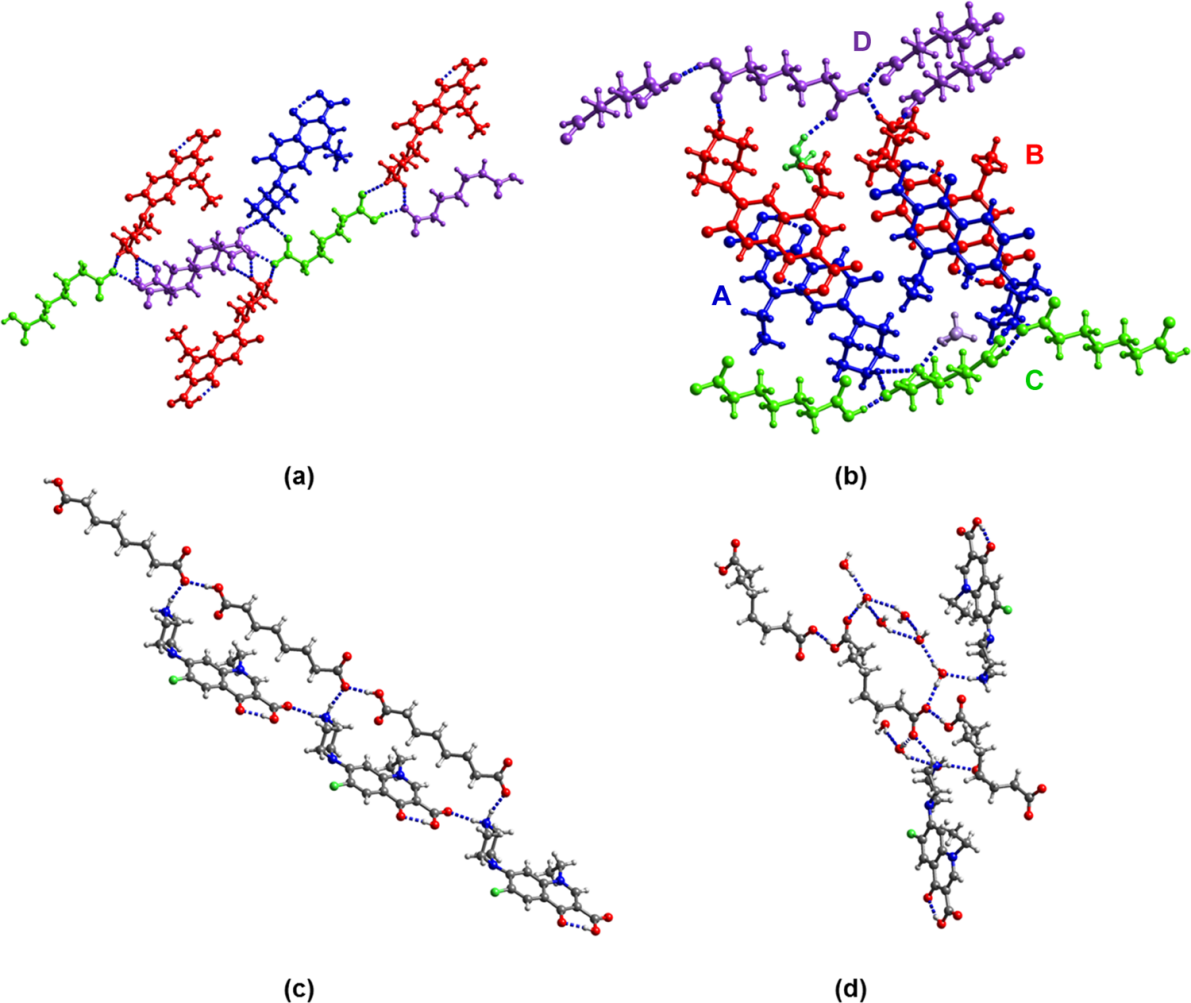
Hydrogen-bonding motifs in (a) (nor^+^)(pim^–^), (b) (nor^+^)(pim^–^)·CH_3_OH, (c) (nor^+^)(sub^–^), and (d) (nor^+^)(sub^–^)·3H_2_O. The colors in panels (a) and (b) indicate crystallographically independent cations and anions.

The H-bonding motifs of (nor^+^)(pim^–^), (nor^+^)(sub^–^), (nor^+^)(pim^–^)·CH_3_OH, (nor^+^)(sub^–^)·3H_2_O, (cip^+^)(pim^–^), (enro^+^)(seb^–^), and (enro^+^)(sub^–^)·H_2_O having infinite chains of carboxylic acid monoanions are shown in Figures 4 and S3–S5. The asymmetric unit of (nor^+^)(pim^–^) contains two nor^+^ cations and two monodeprotonated pim^–^. Charge-assisted NH^+...–^OOC and NH^+...^O=C(OH) H bonds create R_2_^3^(8) and R_4_^4^(12) rings (Figure 4a). The methanol solvate of (nor^+^)(pim^–^) has an asymmetric unit with two cations (denoted A and B), two anions (denoted C and D), and two methanol molecules of crystallization. The protonated amino group of A forms a bifurcated H bond with the deprotonated carboxyl group of C, while the protonated amino group of B interacts with the COOH group of D. Furthermore, H bonding between the COO^–^ groups of C and D and methanol is observed. In the anhydrous 1:1 salt of nor and sub, the cations form a 1D arrangement via NH_2_^+...^O=C(OH) H bonds. The nor^+^ and sub^–^ chains are connected through NH_2_^+...–^OOC H bonds so that the structure can be described as fused R_4_^4^(28) rings (Figure 4c). In the corresponding trihydrate (nor^+^)(sub^–^)·3H_2_O, eight-membered rings of H-bonded water molecules are present (R_4_^4^(8) motif; Figure 4d). One of the water molecules of the ring interacts with the carbonyl oxygen of the COOH group of sub^–^. Another water of the ring forms a H bond with another water, which, in turn, H-bonds to the sub-COO^–^ group creating an R_5_^4^(19) motif. Besides the usual NH_2_^+...–^OOC interaction, the protonated amino group of nor^+^ also participates in H bonding with a water molecule of crystallization.

As in (nor^+^)(pim^–^)·CH_3_OH, the R_2_NH_2_^+^ group in (cip^+^)(pim^–^) interacts with both the COO^–^ and the COOH groups of the carboxylate chain (Figure S3). In (enro^+^)(seb^–^) and (enro^+^)(sub^–^)·H_2_O, the amino group donates a H bond to the COO^–^ group only; in the former, this H bond is bifurcated (Figures S4 and S5).

**Figure 5:**
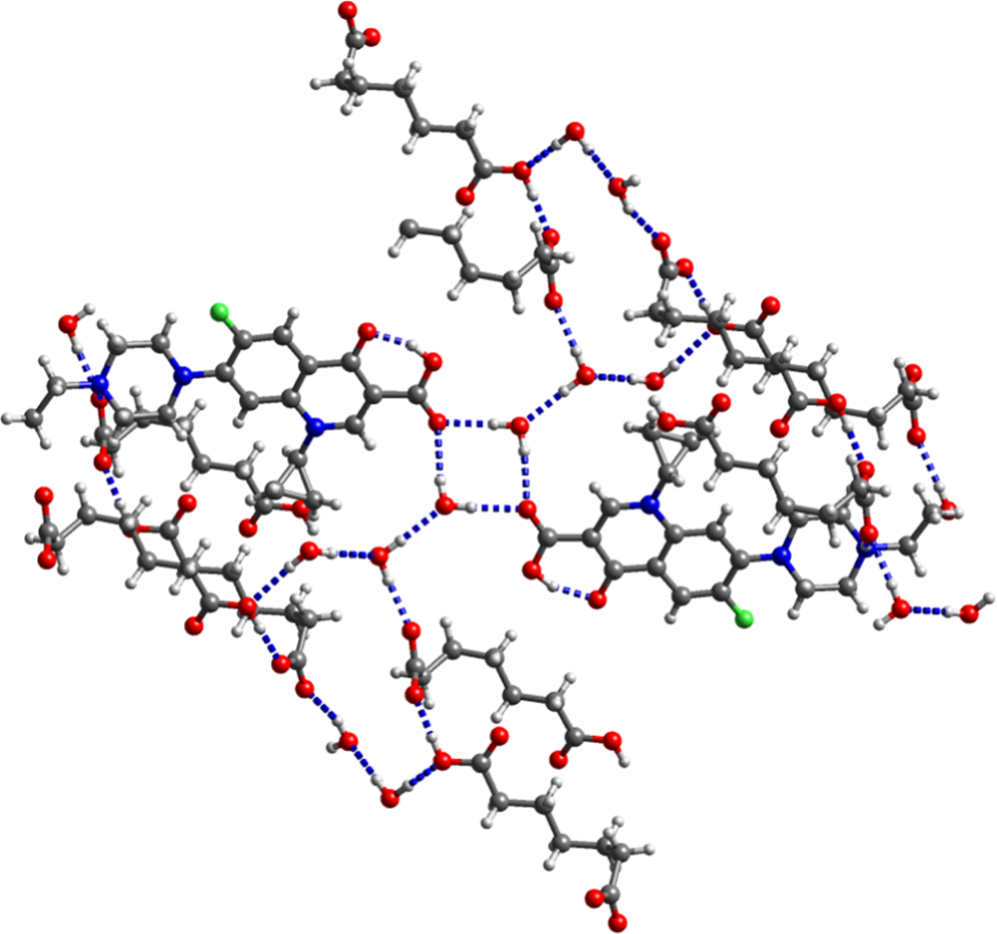
Hydrogen-bonding motif in (enro^+^)(pim^–^)·3H_2_O.

Figure 5 shows the crystal structure of (enro^+^)(pim^–^)·3H_2_O, the only 1:1 salt that does not contain cyclic dimers or 1D chains of H-bonded carboxylate monoanions. Water molecules of crystallization and the COOH and COO^–^ groups of pim^–^ and enro^+^ form R_4_^2^(8) and R_8_^8^(20) rings.

#### Salts of Composition A_2_^+^B^2–^

**Figure 6:**
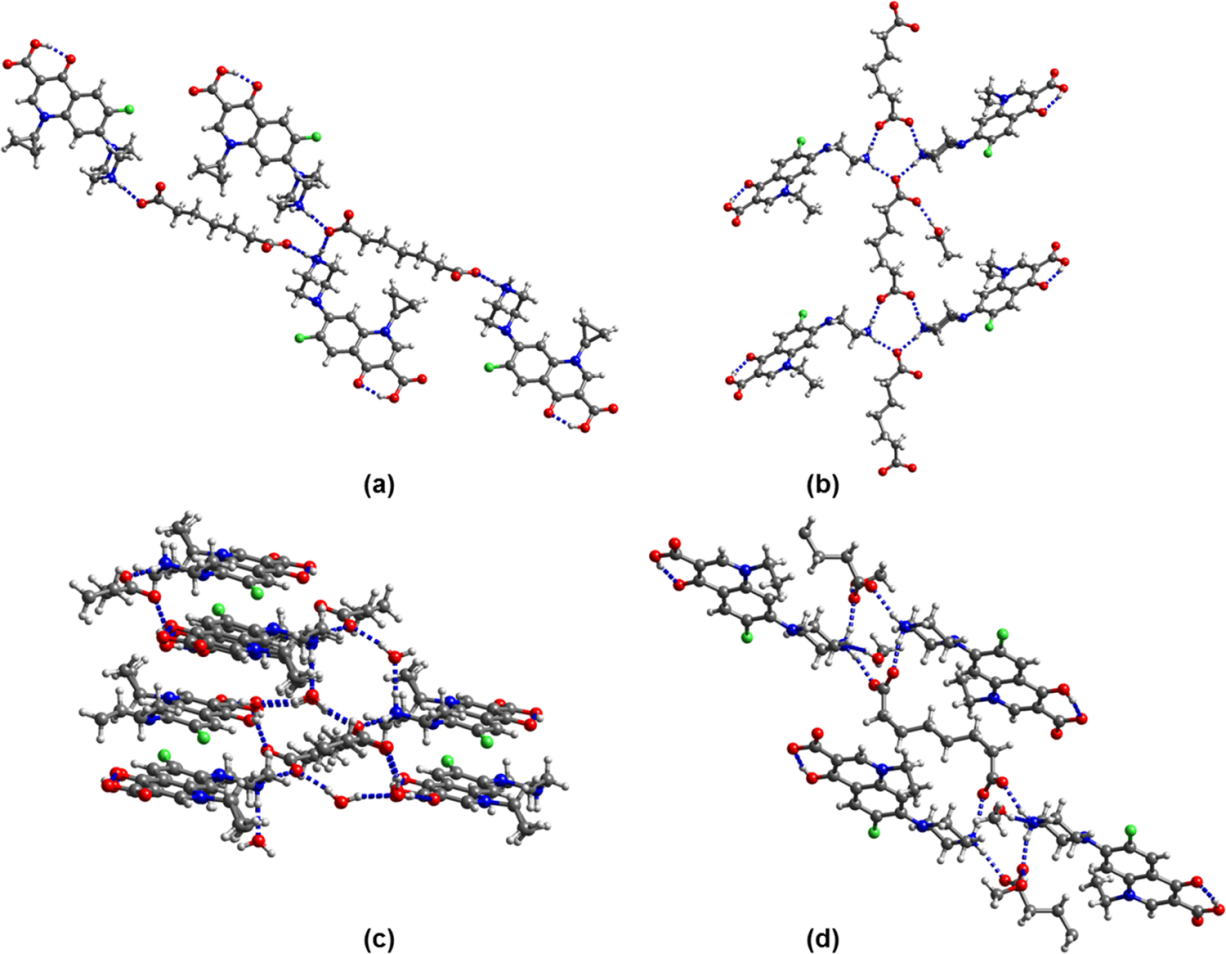
Hydrogen-bonding motifs in (a) (cip^+^)_2_(pim^2–^)·H_2_O, (b) (nor^+^)_2_(pim^2–^)·C_2_H_5_OH, (c) (cip^+^)_2_(sub^2–^)·H_2_O, and (d) (nor^+^)_2_(sub^2–^)·CH_3_OH. For clarity, only one component of the disordered pim^–^ in (nor^+^)_2_(pim^–^)·C_2_H_5_OH and only components A and B of (nor^+^)_2_(sub^2–^)·CH_3_OH are shown. The water of crystallization is omitted in panel (a).

All of the 2:1 salts described in this section crystallize as solvates. The structures of (enro^+^)_2_(pim^2–^)·1.5H_2_O and (nor^+^)_2_(seb^2–^)·4CH_3_OH with both carboxylate groups of the acid dianions forming R_3_NH^+...–^OOC H bonds with the fluoroquinolone cation are shown in the Supporting Information (Figures S6 and S7). Likewise, the structure of (cip^+^)_2_(pim^2–^)·H_2_O is built up by H-bonded cip^+...^pim^2–...^cip^+^ entities (Figure 6a). Two of the carboxylate oxygens of pim^2–^ act as bifurcated H bond acceptors so that the cip^+...^pim^2–...^cip^+^ units are connected to a 2D network. In the ethanol solvate of the corresponding nor salt, (nor^+^)_2_(pim^2–^)·C_2_H_5_OH, R_2_NH_2_^+...–^OOC H bonding between two nor^+^ cations and two pim^2–^ dianions gives 10-membered rings that are linked into an infinite 1D supramolecular structure (R_4_^3^(10); Figure 6b). Neighboring chains are interlocked (Figure S8). The ethanol molecules of crystallization donate H bonds to the carboxylate oxygens of pim^2–^. The structure of (cip^+^)_2_(sub^2–^)·H_2_O contains R_6_^4^(12) and R_3_^3^(15) motifs, formed by two protonated amino groups, two carboxylate oxygens, and two water molecules of crystallization and by two COO^–^ groups and four water molecules, respectively (Figure 6c). The asymmetric unit of (nor^+^)_2_(sub^2–^)·CH_3_OH comprises four nor^+^ cations (denoted A–D), four half-sub^2–^ dianions (A–D), and two methanol molecules of crystallization. Hydrogen bonding between the protonated amino groups of nor^+^ A and B and the carboxylate groups of sub^2–^ A and B gives rise to R_4_^4^(12) rings (Figure 6d). The same motif is observed for nor^+^ and sub^2–^ C and D. The methanol molecules of crystallization donate H bonds to the amino nitrogen and carboxylate oxygen.

**Figure 7:**
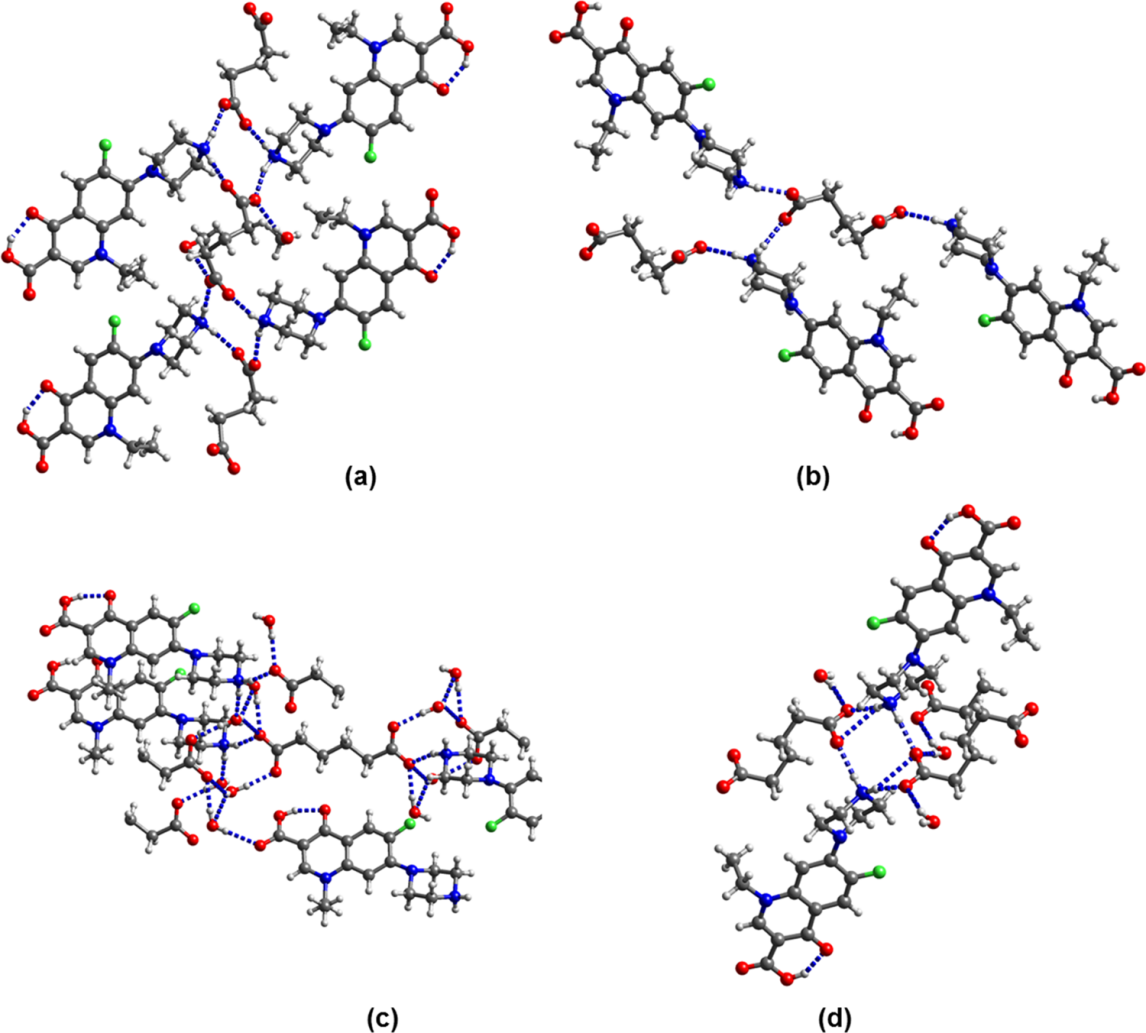
Hydrogen-bonding motifs in (a) (nor^+^)_2_(glu^2–^)·H_2_O·CH_3_OH, (b) (nor^+^)_2_(glu^2–^)·H_2_O·0.75CH_3_CN, (c) (nor^+^)_2_(adi^2–^)·2H_2_O form I, and (d) (nor^+^)_2_(adi^2–^)·2H_2_O form II. For clarity, only one component of the disordered glu^2–^ in (nor^+^)_2_(glu^2–^)·H_2_O·CH_3_OH and only two of the four crystallographically independent nor^+^ ions and one of two crystallographically independent glu^2–^ ions of (nor^+^)_2_(glu^2–^)·H_2_O·0.75CH_3_CN are shown. Water and solvent molecules of crystallization are not shown in panels (a) and (b).

The crystal structures of two different solvates of the 2:1 salt of nor and glu are shown in Figure 7a,b. (Nor^+^)_2_(glu^2–^)·H_2_O·CH_3_OH R_4_^4^(12) rings of two amino groups and two carboxylate groups extend as R_4_^4^(12)^...^glu^2–...^R_4_^4^(12)^...^glu repeats in one dimension. Further H bonding occurs between the methanol OH group and carboxylate oxygen. In the mixed hydrate–acetonitrile solvate (nor^+^)_2_(glu^2–^)·H_2_O·0.75CH_3_CN, three of the four carboxylate oxygens participate in H bonding with three nor^+^ cations (Figure 7b).

Two polymorphs of (nor^+^)_2_(adi^2–^)·2H_2_O were obtained from water and methanol/acetonitrile (1:1), respectively, denoted form I and form II (Figure 7c,d). The H-bonding interactions between water molecules and COO^–^ groups of adi^2–^ generate R_4_^4^(12) rings and R_2_^2^(4) “triangles” in form I. One of the amino protons of nor^+^ forms H bonds with COO^–^ and water oxygens. There is also H bonding between the carboxyl group of nor^+^ and water of crystallization. The main structural motif in (nor^+^)_2_(adi^2–^)·2H_2_O form II is the R_4_^2^(8) motif of two carboxylate oxygens and two amino groups.

#### Salts of Compositions A_2_^+^B^2–^B and A^+^B^–^A

**Figure 8:**
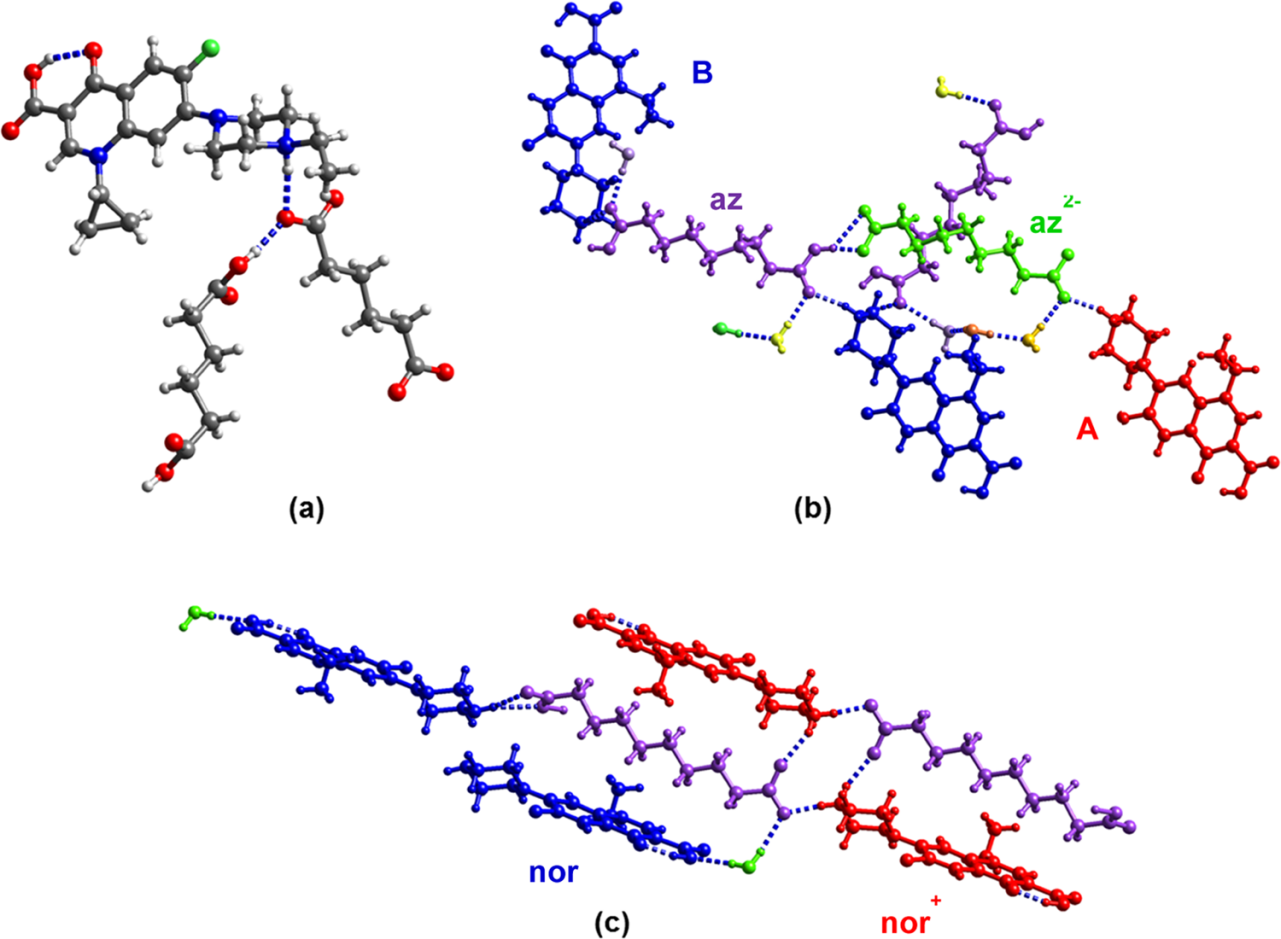
Hydrogen-bonding motifs in (a) (enro^+^)_2_(adi^2–^)·adi·CH_3_CN, (b) (nor^+^)_2_(az^2–^)·az·4H_2_O: red, nor^+^ A; blue, nor^+^ B; green, az^2–^; and purple, az; and (c) (nor^+^)(seb^–^)·nor·H_2_O: red, nor^+^; blue, nor. Solvent molecules of crystallization are not shown in panel (a).

Cocrystallizing enro with adi from acetonitrile and nor with az from methanol gave two new stoichiomorphs of enro/adi and nor/az that can be classified as conjugate acid/base molecular ionic cocrystals.^34^ The H-bonding motifs in (enro^+^)_2_(adi^2–^)·adi·CH_3_CN and (nor^+^)_2_(az^2–^)·az·4H_2_O are shown in Figure 8. (Enro^+^)_2_(adi^2–^)·adi·CH_3_CN has the R_3_NH^+...–^O(CO)^...^HOOC motif. Two crystallographically independent nor^+^ ions (A and B), one az^2–^ dianion, a neutral az molecule, and four water molecules of crystallization make up the asymmetric unit of (nor^+^)_2_(az^2–^)·az·4H_2_O. One of the carboxylate groups of az^2–^ forms a H bond with the protonated amino group of nor^+^ A. The other COO^–^ group of az^2–^ participates in a bifurcated H-bonding interaction with the COOH group of az. The carbonyl oxygen of the neutral az interacts with the amino group of nor^+^ B and with a water molecule of crystallization. Three water molecules, the COOH group of az, a carbonyl oxygen of another az, the amino group of nor^+^ B, and az^2–^ are arranged in an R_7_^6^(26) motif.

Figure 8c shows the H bonding in the molecular ionic cocrystal (nor^+^)(seb^–^)·nor·H_2_O. Two protonated amino groups and two COO^–^ groups form the frequently found R_4_^4^(12) synthon. The amino proton of the neutral nor molecule interacts with both oxygens of the COOH group of seb^–^ in a bifurcated H bond. The carboxyl group of seb^–^ is in the anti-conformation (τ = 176.3°) and donates a H bond to the COO^–^ group of an adjacent seb^–^. The water molecule forms H bonds with the carboxylate oxygen of seb^–^ and with the hydroxyl oxygen of the nor COOH group.

Some of the salts characterized by single-crystal X-ray analysis, namely, (cip^+^)_2_(pim^2–^)·H_2_O, (nor^+^)_2_(adi^2–^)·2H_2_O form II, (enro^+^)(pim^–^)·H_2_O, (enro^+^)(seb^–^), (nor^+^)_2_(pim^2–^)·C_2_H_5_OH, (nor^+^)_2_(az^2–^)·az·4H_2_O, and (nor^+^)(seb^–^)·nor·H_2_O, could not be prepared in bulk quantities and were therefore excluded from further studies. Solvates that were not pharmaceutically acceptable were also excluded. The compositions of the bulk crystalline samples of the remaining anhydrous salts and hydrates, (enro^+^)_2_(pim^2–^)·1.5H_2_O, (enro^+^)(pim^–^)·3H_2_O, (enro^+^)(sub^–^)·H_2_O, (enro^+^)(az^–^), (nor^+^)_2_(adi^2–^)·2H_2_O form I, (nor^+^)(pim^–^), (nor^+^)(sub^–^)·3H_2_O, (nor^+^)(sub^–^), (nor^+^)_2_(seb^2–^)·3H_2_O, (cip^+^)(glu^–^), (cip^+^)_2_(sub^2–^)·4H_2_O, and (cip^+^)(pim^–^), were confirmed by comparing the XRPD patterns with the simulated patterns of the single crystals (Figures S9–S18).

### Thermal Analysis

The DSC and TGA plots of the anhydrous salts and hydrates are shown in Figures S19–S30. The DSC plots of the anhydrates (cip^+^)(pim^–^) and (cip^+^)(glu^–^) show only one endotherm due to melting at 195.0 and 229.2 °C, respectively. In both cases, the melting point lies between those of cip (255–257 °C) and the coformer (pim: 103–105 °C; glu: 97.8 °C). (Nor^+^)(pim^–^) gives a melting endotherm at 201.9 °C. Another endotherm at 181.4 °C indicates that a solid-state transformation takes place prior to melting. Melting of the salt is followed by melting and decomposition of nor at 227.9 °C.

The TGA plots of (cip^+^)_2_(sub^2–^)·4H_2_O, (nor^+^)_2_(adi^2–^)·2H_2_O form I, (enro^+^)_2_(pim^2–^)·1.5H_2_O, (nor^+^)_2_(seb^2–^)·3H_2_O, and (enro^+^)(sub^–^)·H_2_O are in agreement with the compositions as tetra-, di-, sesqui-, tri-, and monohydrates. The loss of the water in (cip^+^)_2_(sub^2–^)·4H_2_O occurs as two endothermic events at 70.3 and 87.3 °C. The total weight loss in the TGA plot agrees with the calculated percentage of water (exp. 7.5%, calcd 7.9%). The endotherm at 86.9 °C in the DSC plot of (nor^+^)_2_(adi^2–^)·2H_2_O form I is accompanied by a 4.1% weight loss in the TGA corresponding to two water molecules of crystallization (calcd 4.4%). The melting endotherms of (cip^+^)_2_(sub^2–^)·4H_2_O and (nor^+^)_2_(adi^2–^)·2H_2_O form I give peaks at 232.8 and 230.2 °C, respectively.

(Enro^+^)_2_(pim^2–^)·1.5H_2_O is dehydrated at 119.1 °C (observed weight loss: 3.3%, calcd 3.0%). Two broad endotherms at 202.8 and 255.9 °C in the DSC plot of (enro^+^)_2_(pim^2–^)·1.5H_2_O are due to melting and decomposition. The dehydration endotherm of (enro^+^)(sub^–^)·H_2_O is observed at 98.4 °C. The shoulder around 79 °C and the deviation of the experimental weight loss from the calculated value 4.2% (exp.) vs 3.3% (calcd) indicate the presence of traces of surface water that could not be removed despite prolonged drying of the sample. The endothermic events at 183.4 and 266 °C are attributed to melting and decomposition.

The water of crystallization of (nor^+^)_2_(seb^2–^)·3H_2_O is lost at 70.3 °C. A broad endotherm with shoulders covers the 90–175 °C range, suggesting that the anhydrous form undergoes several solid-state transformations before melting at 208.6 °C.

The DSC plot of (enro^+^)(pim^–^)·3H_2_O shows three endothermic events at 93.8, 111.8, and 127.5 °C. However, only the endotherm at 93.8 °C is associated with a loss of one water molecule of crystallization in the TGA plot (exp. 3.1%; calcd 3.1%). The endotherms at 183.9 and 255.2 °C are due to melting and decomposition. The XRPD pattern of the bulk sample clearly matches the simulated pattern of the single-crystal structure (Figure S9). No additional peaks are observed so that the XRPD analysis indicates a phase-pure bulk sample. We do not have an explanation for the discrepancy between the TGA and XRPD data.

In the DSC plot of (nor^+^)(sub^–^)·3H_2_O, three endothermic events occur at 82.8, 136.3, and 204.1 °C that can be assigned to dehydration, structural rearrangement of the dehydrated form, and melting. When the trihydrate is heated to 140 °C for 15 min before the DSC analysis, the only thermal event in the thermogram is melting at 211.9 °C. This is different from the thermal behavior of the anhydrous (nor^+^)(sub^–^) salt prepared by solution crystallization that melts at 183.1 °C followed by crystallization of nor at 200.2 °C followed by melting of nor at 228.8 °C.

The DSC plot of (enro^+^)(az^–^) shows endotherms at 144.7, 173.4, and 263.5 °C due to melting of the salt, crystallization of enro, and melting of enro.

As often observed for salts and cocrystals, the salts have melting points that are between the melting points of the respective two components. The only exception is (nor^+^)_2_(adi^2–^)·2H_2_O form I, the melting point of which is higher than those of nor and adi.

### Solubility

The solubility of the anhydrous salts and hydrates that could be crystallized in bulk quantities was determined in phosphate buffer (pH 6.8, 37 °C) and compared with that of the parent fluoroquinolones (Table 1). To investigate whether the salts undergo phase transformations in solution, the solid phases were recovered after the solubility measurement and analyzed by XRPD (Figures S9–S18). In all cases, changes in the XRPD pattern were observed. Additional peaks or new patterns appeared, indicating that the salts transform in the dissolution medium. However, it was not possible to unambiguously identify possible dissociation or transformation products. The solubilities obtained for cip (0.099 ± 0.001 mg mL^–1^), nor (0.466 ± 0.003 mg mL^–1^), and enro (0.289 ± 0.003 mg mL^–1^) are in good agreement with data previously reported in the literature.^17,35,36^ For the supramolecular salts, 2.3- to 15.6-fold increases in solubility were observed. The 2:1 salt (enro^+^)_2_(pim^2–^)·1.5H_2_O was found to be less soluble than the corresponding 1:1 salt (enro^+^)(pim^–^)·3H_2_O. The highest solubility enhancement over the parent fluoroquinolone occurs for (cip^+^)(glu^–^) (7.3-fold), (enro^+^)(sub^–^)·H_2_O (11.7-fold), (cip^+^)(pim^–^) (9.6-fold), and (enro^+^)(az^–^) (15.6-fold). (Enro^+^)(az^–^) contains discrete enro^...^acid dimer^...^enro entities, while in (cip^+^)(pim^–^), chains of monocarboxylate anions are present. Neither of these two salts contain supramolecular rings of fluoroquinolone cations and acid anions. Noteworthily, (enro^+^)(az^–^) has the lowest melting point of the studied salts, indicating a low lattice energy. (Cip^+^)(glu^–^), which shows a 7.3-fold solubility enhancement, contains a highly soluble coformer. However, overall, there is no clear correlation between the solubility of the coformer and the apparent solubility of the supramolecular salt.

**Table 1:** Apparent Solubility of the Supramolecular Salts in Phosphate Buffer, pH 6.8, 37 °C

compound	solubility (mg mL^–1^)	solubility enhancement over parent fluoroquinolone
enro	0.289 ± 0.003	
(enro^+^)_2_(pim^2–^)·1.5H_2_O	0.668 ± 0.003	2.31
(enro^+^)(pim^–^)·3H_2_O	1.079 ± 0.001	3.73
(enro^+^)(sub^–^)·H_2_O	3.386 ± 0.004	11.69
(enro^+^)(az^–^)	4.503 ± 0.002	15.55
nor	0.466 ± 0.003	
(nor^+^)_2_(adi^2–^)·2H_2_O form I	1.147 ± 0.003	2.46
(nor^+^)(pim^–^)	1.373 ± 0.004	2.95
(nor^+^)(sub^–^)·3H_2_O	1.294 ± 0.004	2.78
(nor^+^)(sub^–^)	1.240 ± 0.006	2.66
(nor^+^)_2_(seb^2–^)·3H_2_O	1.125 ± 0.006	2.42
cip	0.099 ± 0.001	
(cip^+^)(glu^–^)	0.724 ± 0.005	7.33
(cip^+^)(pim^–^)	0.949 ± 0.006	9.62
(cip^+^)_2_(sub^2–^)·4H_2_O	0.317 ± 0.004	3.04

### Gelator Properties

**Figure 9:**
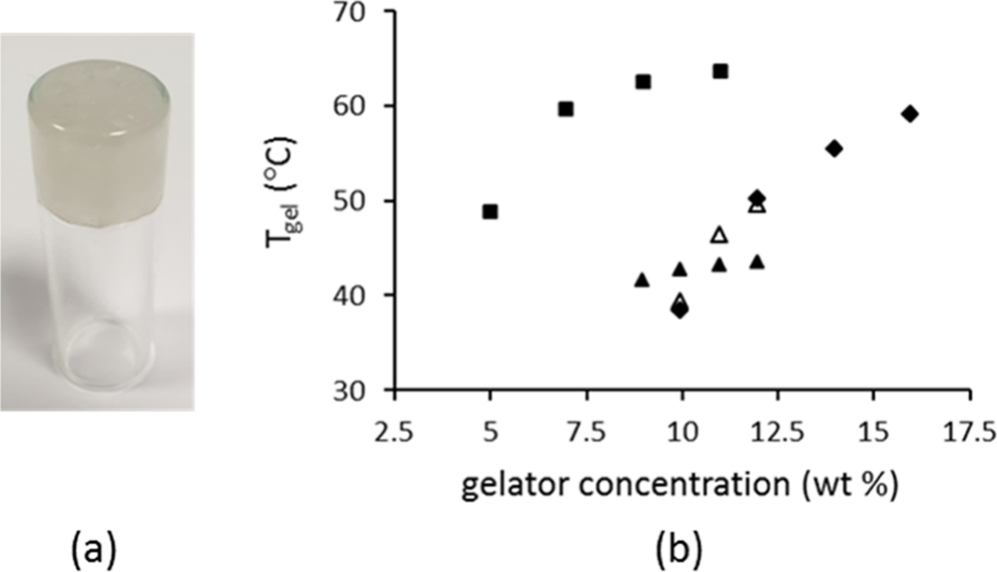
(a) Gel formation of nor/az/propylene glycol. (b) Plot of *T*_gel_ (°C) vs gelator concentration (wt %) for cip/sub/propylene glycol (solid squares), nor/az/propylene glycol (solid diamonds), enro/sub/propylene glycol (solid triangles) and enro/sub/H_2_O (open triangles).

Enro/glu, cip/glu, nor/glu, enro/adi, cip/adi, nor/adi, enro/pim, nor/pim, enro/sub, cip/sub, enro/az, nor/az, cip/seb, and enro/seb mixtures formed gel-like precipitates in some of the solvents used in the crystallization experiments. We therefore performed gelation studies with eight solvents including water and three solvents classified by the FDA as GRAS (generally recognized as safe): ethanol, propylene glycol, and polyethylene glycol (PEG-400). Mixtures in the ratio of 1:1 and 2:1 of the respective fluoroquinolone and carboxylic acid were ball-milled, and salt formation was confirmed by X-ray powder diffraction (Figures S31–S48). For most mixtures, different patterns were observed for the 1:1 and 2:1 ratios indicating different salt products. In the case of cip/az, cip/adi, cip/pim, cip/glu, enro/az, and enro/glu, the patterns of the 2:1 mixtures showed the peaks of the 1:1 salt and free fluoroquinolone. The milled samples were screened for their gelator properties by heating them at 1–10% concentrations in the respective solvent followed by slow cooling to room temperature. Gel formation was initially assessed by visual inspection and the test tube inversion method (Figure 9a), and the results are summarized in Table S10. For 26 of the 216 salt/solvent combinations, gelation occurred. Most of the salts were not soluble in ethanol, and none gelled the alcohol. Cip/glu (1:1), nor/glu (2:1), enro/glu (2:1), cip/adi (1:1), nor/adi (1:1), nor/adi (2:1), enro/adi (2:1), nor/pim (2:1), enro/pim (1:1), enro/pim (2:1), enro/sub (1:1), enro/sub (2:1), cip/sub (1:1), nor/az (1:1), enro/az (2:1), cip/seb (1:1), enro/seb (1:1), and enro/seb (2:1) proved to be gelators in at least one of the other GRAS solvents. Four of these systems were selected for further studies based on their appearance and apparent strength of the gel: nor/az 1:1 (propylene glycol), cip/sub 1:1 (propylene glycol), enro/sub 1:1 (propylene glycol), and enro/sub 1:1 (H_2_O). The gel-dissociation temperatures *T*_gel_ were determined, and the plots of *T*_gel_ vs gelator concentration are displayed in Figure 9b. As expected for supramolecular gels, *T*_gel_ increases with increasing gelator concentration. *T*_gel_ of 10% (wt) salt solutions ranges from 40 to 60 °C. The gels are thermoreversible, i.e., dissolve on heating and re-form on cooling. The stability of the gels (10 wt % gelator concentration) was monitored at ambient temperature and 56% relative humidity. Enro/sub/H_2_O and nor/az/propylene glycol did not undergo any visible changes for 2 weeks, and their *T*_gel_ decreased by <3 °C. By contrast, a 9 °C reduction in *T*_gel_ was observed for the aged enro/sub/propylene glycol and cip/sub/propylene glycol gels after 2 weeks.

The flow behavior of the four gels (10 wt % gelator concentration) was further characterized by dynamic rheology measurements. Amplitude sweep measurements in the linear viscoelastic region (Figure S49) suggested that frequency sweep experiments, which are required to ascertain the viscoelastic nature of the gels, should be performed at a constant strain of 0.1%. The corresponding frequency sweep measurements for these systems showed the expected characteristics for viscoelastic materials. In the case of nor/az/propylene glycol, enro/sub/propylene glycol, and enro/sub/H_2_O, the elastic modulus values (*G*′) were found to be larger than the viscous loss modulus values (*G*″) (Figure S50) and were largely frequency-invariant on a longer time scale, which is typical of viscoelastic materials such as supramolecular gels. In the case of cip/sub/propylene glycol, *G*′ was found to cross *G*″, which indicates a viscoelastic solid-like behavior and suggests that this gel structure undergoes sedimentation at higher frequencies.

**Figure 10:**
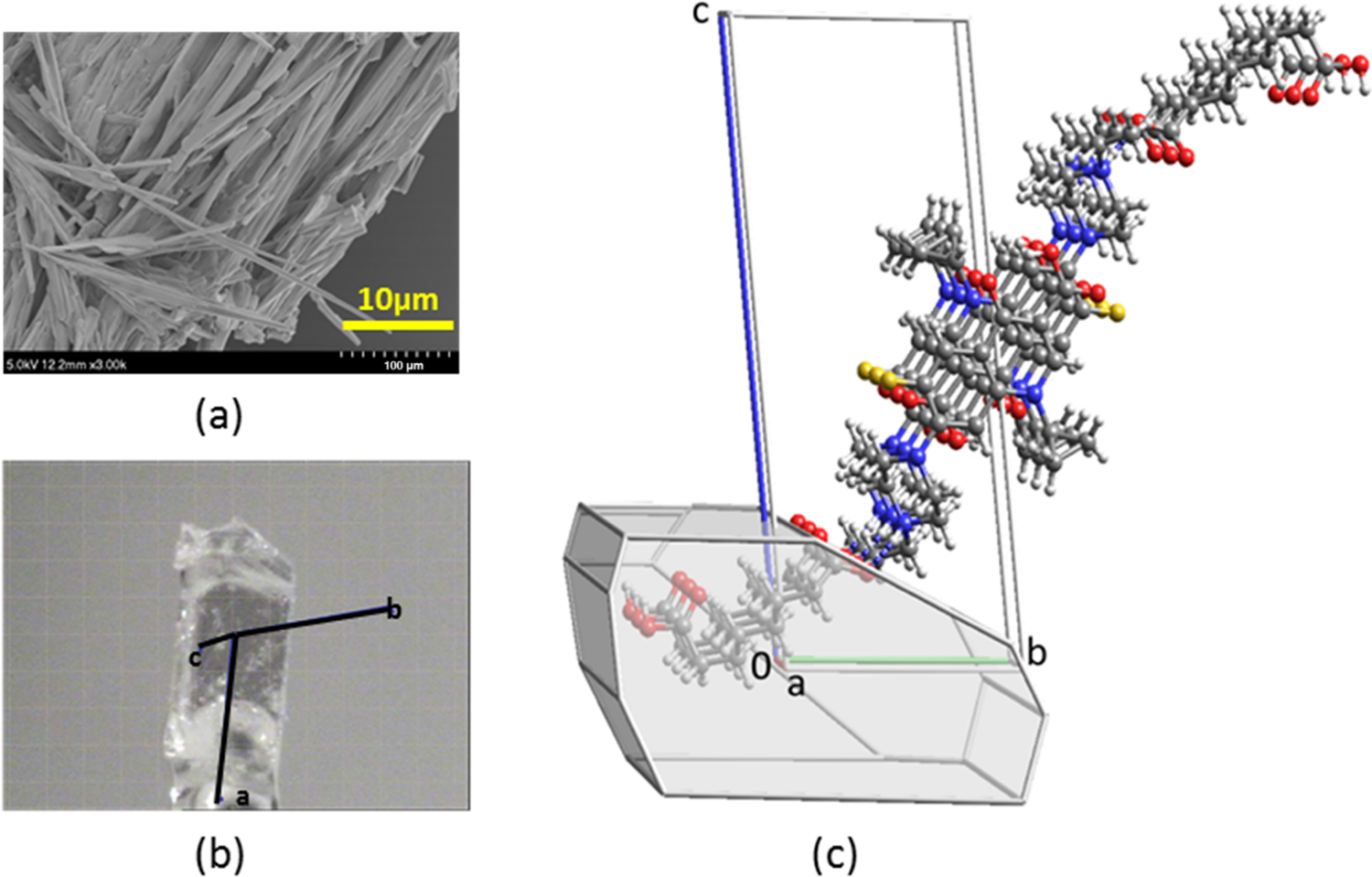
(a) Scanning electron microscopic image of the enro/sub/propylene glycol xerogel. (b) Single crystal of (enro^+^)(sub^–^)·H_2_O indexed on the diffractometer. (c) Unit cell diagram of (enro^+^)(sub^–^)·H_2_O.

As described above, the gelation of low-molecular-weight gelators results from molecules self-assembling through supramolecular interactions. In particular, anisotropic noncovalent interactions such as 1D H bonding are responsible for the formation of 1D nanofibers that induce gelation. Figure 10a shows the scanning electron microscopic image of the dried enro/sub/propylene glycol gel. Needles of >10 μm length formed. Likewise, long needles were observed in the micrograph of the nor/az/propylene glycol xerogel (Figure S51a). The enro/sub/H_2_O gel formed needles and elongated blocks on drying (Figure S51c), while cip/sub/propylene glycol that showed viscoelastic solid-like behavior gave irregular plates. To gain insight into the structures of the gels, the XRPD patterns of the dried gels were recorded and compared with the theoretical patterns calculated from the crystal data of (enro^+^)(sub^–^)·H_2_O and (nor^+^)_2_(az^2–^)(az)·4H_2_O. The XRPD pattern of the enro/sub/H_2_O xerogel is a good match with that of the (enro^+^)(sub^–^)·H_2_O single crystal (Figure S52). Indexation of the single crystal revealed needle growth in the *a* axis direction (Figure 10b). In the single-crystal structure of (enro^+^)(sub^–^)·H_2_O, the carboxylate monoanions are linked into infinite H-bonded chains. The enro^+^ cations are connected to the carboxylate chain via R_3_NH^+...–^OOC H bonding and are stacked along the *a* direction with 61.2% of the atoms in the van der Waals contact, with several C^...^C contacts close to 3.6 Å. (Figure 10c). We have shown in previous papers that neutral molecules stacking within a 1D motif with more than −30 kJ mol^–1^ interaction energy and at least 50% van der Waals contact will drive needle growth and that stacking interactions of this type are more important for needle growth than H bonding.^37,38^ It may appear surprising that crystal growth could be driven in a similar way when the stacking interaction involves cations, which might be expected to repel each other. However, close contact anion stacking has been observed in croconic acid salts and has been attributed to “electrostatic compression”^39^ or “Coulombic compression”.^40^ The strong Coulombic forces, which attract cation–anion pairs to each other, “compress” the stacked ions closer together than might have been expected. The net interaction energy between the enro^+^ cations in the stacks may indeed be repulsive, but this is overcome by the strong ionic forces that drive the addition of H-bonded ion pairs across inversion centers to the growing crystal. The efficient packing provided by flat molecule stacking may also be an important factor influencing crystal growth. The creation of 1D motifs with hydrophilic regions is the classic requirement for the gelation of water.^41^ Yadav et al. studied the gelator properties of 24 cocrystals of 2-aminothiazole with dicarboxylic acids.^42^ One of the cocrystals was able to gel water, and this was attributed to the presence of hydrophilic cavities that immobilize water molecules. In addition to NH^+...–^OC(R)=O^...^HOOC (hydrophilic regions) structures, the crystal structure of (enro^+^)(sub^–^)·H_2_O shows unused H-bonding capacity around the fluoroquinolone that makes this class of organic salts good gel formers.

The XRPD patterns of the dried enro/sub/propylene glycol and nor/az/propylene glycol gels do not match the simulated patterns of the structurally characterized enro/sub and nor/az salts (Figures S52 and S53) so that no assumption regarding the presence or absence of 1D motifs in these gels can be made.

## Conclusions

Salts of the fluoroquinolones cip, nor, and enro and α,ω-carboxylic acids HOOC-(CH_2_)_n_-COOH with *n* = 3–8 have rich solid-state landscapes. The salts have A^+^B^–^, A_2_^+^B^2–^, A_2_^+^B^2–^B, and A^+^B^–^A compositions. Several of the salts could not be obtained as pure phases in bulk quantities, which hindered a systematic study of the correlation between structure and solubility and of the effect of the spacer length. The 1:1 salts of enro/sub and nor/az are low-molecular-weight gelators, and in the case of enro/sub, the gelation ability can be attributed to the 1D stacking motif in the crystal structure.

## Supporting Information Available

Experimental details of solution crystallization experiments; crystal data; H-bonding interactions; and figures of the X-ray structures of enro.CH_3_CN, (enro^+^)(glu^–^)·0.33CH_3_CN·0.67H_2_O, (cip^+^)(pim^–^), (enro^+^)(seb^–^), (enro^+^)(sub^–^)·H_2_O, XRPD patterns, DSC, and TGA plots (PDF)

## Accession Codes

CCDC 2128064–2128092 contain the supplementary crystallographic data for this paper. These data can be obtained free of charge via www.ccdc.cam.ac.uk/data_request/cif, or by emailing data_request@ccdc.cam.ac.uk, or by contacting The Cambridge Crystallographic Data Centre, 12 Union Road, Cambridge CB2 1EZ, UK; fax: +44 1223 336033.
