# Supplementary material for: Formation of Salts and Molecular Ionic Cocrystals of Fluoroquinolones and α,ω-Dicarboxylic Acids
Source: Cryst Growth Des. 2022 Apr 11;22(5):3060–71. doi: 10.1021/acs.cgd.1c01509 (PMC9073931; doi:10.1021/acs.cgd.1c01509)
Supplement: Supplementary file 1 — cg1c01509_si_001.pdf [file cg1c01509_si_001.pdf]

Formation of Salts and Molecular Ionic Cocrystals of Fluoroquinolones and α,ω-Dicarboxylic Acids

Ciaran O'Malley,[1] Patrick McArdle,[1,*] and Andrea Erxleben[1,2,*]

[1] School of Chemistry, National University of Ireland, Galway, H91TK33, Ireland

[2] Synthesis and Solid State Pharmaceutical Centre (SSPC), Limerick, V94T9PX, Ireland

*Corresponding author email address: andrea.erxleben@nuigalway.ie (AE);

p.mcardle@nuigalway.ie (PM)

Supporting Information

Table S1. Solution crystallization experiments for supramolecular salts of enro.

Table S2. Solution crystallization experiments for supramolecular salts of nor.

Table S3. Solution crystallization experiments for supramolecular salts of cip.

Table S4. Crystallographic data of the supramolecular salts of enro.

Table S5. Crystallographic data of the supramolecular salts of nor (1/2).

Table S6. Crystallographic data of the supramolecular salts of nor (2/2).

Table S7. Crystallographic data of the supramolecular salts of cip.

Table S8. Crystallographic data of enro·CH_3CN and enro·H_2O.

Table S9. H bonding interactions in the salts of nor, cip and enro.

Table S10. Results of the screening of the fluoroquinolone salts for gelator properties by heating followed by slow cooling to room temperature (salt : solvent = 1 : 10 (w/w)).

Figure S1. Crystal structures of enro·CH_3CN and enro·H_2O.

Figure S2. H bonding motif and crystal packing of $(enro^+)(glu^-)\cdot 0.33CH_3CN\cdot 0.67H_2O$.

Figure S3. H bonding motif in $(cip^+)(pim^-)$.

Figure S4. H bonding motif in $(enro^+)(seb^-)$.

Figure S5. H bonding motif in $(enro^+)(sub^-)\cdot H_2O$.

Figure S6. H bonding motif in $(enro^{+})_2(pim^{2-})\cdot 1.5H_2O$.

Figure S7. H bonding motif in $(nor^{+})_2(seb^{2-})\cdot 4CH_3OH$.

Figure S8. Crystal packing of $(nor^{+})_2(pim^{2-})\cdot C_2H_5OH$.

Figures S9 – S18. XRPD patterns of the solid phases before and after the solubility measurement.

Figures S19 – S30. DSC and TGA plots**.**

Figures S31 – S48. XRPD patterns of the salts prepared by ball-milling.

Figure S49. Rheological response (oscillatory amplitude sweep) of supramolecular gels of cip/sub from propylene glycol, nor/az from propylene glycol, enro/sub from propylene glycol and enro/sub from water.

Figure S50. Rheological response (frequency sweep) of supramolecular gels of cip/sub from propylene glycol, nor/az from propylene glycol, enro/sub from propylene glycol and enro/sub from water.

Figure S51. Scanning electron microscopy image of the (a) nor/az/propylene glycol, (b) cip/sub/propylene glycol and (c) enro/sub/water xerogels.

Figure S52. XRPD pattern of the dried nor/az/propylene glycol gel.

Figure S53. XRPD patterns of the dried enro/sub/H_2O and enro//sub/propylene glycol gels.

Table S1. Solution crystallization experiments for supramolecular salts of enro.

Coformer	**Solvent**	**Result**
Glu	Acetonitrile	Crystallization of $(enro^{+})(glu^{-})\cdot0.33CH_3CN\cdot0.67H_2O$
Glu	1:1 Acetonitrile/methanol	Powder
Glu	Water	Powder
Adi	Methanol	Crystallization of $enro\cdot H_2O$
Adi	Ethanol	Powder
Adi	Acetonitrile	Crystallization of $(enro^{+})_2(adi^{2-})\cdot adi\cdot2CH_3CN$
Adi	1:1 Acetonitrile/methanol	Poor quality crystals
Adi	Water	Powder
Pim	Methanol	Crystallization of $(enro^{+})_2(pim^{2-})\cdot1.5H_2O$
Pim	Ethanol	Powder
Pim	Acetonitrile	Crystallization of $(enro^{+})(pim^{-})\cdot H_2O$
Pim	1:1 Acetonitrile/methanol	Crystallization of $enro\cdot CH_3CN$
Pim	Water	Crystallization of $(enro^{+})(pim^{-})\cdot3H_2O$
Sub	Methanol	Powder
Sub	Ethanol	Poor quality crystals
Sub	Acetonitrile	Crystallization of $(enro^{+})(sub^{-})\cdot H_2O$
Sub	1:1 Acetonitrile/methanol	Powder
Sub	Water	Crystallization of $(enro^{+})(sub^{-})\cdot H_2O$
Az	Methanol	Poor quality crystals
Az	Ethanol	Powder
Az	Acetonitrile	Crystallization of $(enro^{+})(az^{-})$
Az	1:1 Acetonitrile/methanol	Powder
Az	Water	Crystallization of $(enro^{+})(az^{-})$
Seb	Acetonitrile	Crystallization of $(enro^{+})(seb^{-})$
Seb	1:1 Acetonitrile/methanol	Poor quality crystals
Seb	Water	Powder

Table S2. Solution crystallization experiments for supramolecular salts of nor.

Coformer	**Solvent**	**Result**
Glu	Methanol	Crystallization of $(nor^{+})_2(glu^{2-})\cdot H_2O\cdot CH_3OH$
Glu	Acetonitrile	Crystallization of $(nor^{+})_2(glu^{2-})\cdot H_2O\cdot 0.75CH_3CN$
Glu	1:1 Acetonitrile/Methanol	Crystallization of $(nor^{+})_2(glu^{2-})\cdot H_2O\cdot CH_3OH$
Glu``	Water	Powder
Adi	Methanol	Crystallization of LOQKOT
Adi	Ethanol	Poor quality crystals
Adi	Acetonitrile	Powder
Adi	1:1 Acetonitrile/Methanol	Crystallization of known salt $(nor^{+})_2(adi^{2-})\cdot 2H_2O$ Form II
Adi	Water	Crystallization of $(nor^{+})_2(adi^{2-})\cdot 2H_2O$ Form I
Adi	Acetone	Powder
Adi	Ethyl Acetate	Powder
Pim	Methanol	Crystallization of $(nor^{+})(pim^{-})\cdot CH_3OH$
Pim	Ethanol	Crystallization of $(nor^{+})_2(pim^{2-})\cdot C_2H_5OH$
Pim	Acetonitrile	Crystallization of $(nor^{+})(pim^{-})$
Pim	1:1 Acetonitrile/Methanol	Crystallization of $(nor^{+})(pim^{-})\cdot CH_3OH$
Pim	Water	Powder
Pim	Acetone	Powder
Pim	Ethyl Acetate	Powder
Sub	Methanol	Crystallization of $(nor^{+})_2(sub^{2-})\cdot CH_3OH$
Sub	Ethanol	Crystallization of $(nor^{+})(sub^{-})$
Sub	Acetonitrile	Powder
Sub	1:1 Acetonitrile/Methanol	Crystallization of $(nor^{+})(sub^{-})$
Sub	Water	Crystallization of $(nor^{+})(sub^{-})\cdot 3H_2O$
Az	Methanol	Crystallization of $(nor^{+})_2(az^{2-})\cdot az\cdot 4H_2O$
Az	Ethanol	Poor quality crystals
Az	Acetonitrile	Poor quality crystals
Az	1:1 Acetonitrile/Methanol	Powder
Az	Water	Crystallization of $(nor^{+})_2(az^{2-})\cdot az\cdot 4H_2O$

Az	Toluene	Poor quality crystals
Seb	Methanol	Crystallization of $(nor^{+})_2(seb^{2-})\cdot 4CH_3OH$
Seb	Acetonitrile	Crystallization of $(nor^{+})(seb^{-})\cdot nor\cdot H_2O$
Seb	1:1 Acetonitrile/Methanol	Crystallization of $(nor^{+})(seb^{-})\cdot nor\cdot H_2O$
Seb	Water	Crystallization of $(nor^{+})_2(seb^{2-})\cdot 3H_2O$

Table S3. Solution crystallisation experiments for supramolecular salts of cip.

Coformer	**Solvent**	**Resul**
Glu	Methanol	Powder
Glu	Acetonitrile	Crystallization of $(cip^{+})(glu^{-})$
Glu	1:1 Acetonitrile/Methanol	Powder
Glu	Water	Powder
Adi	Methanol	Poor quality crystals
Adi	Ethanol	Poor quality crystals
Adi	Acetonitrile	Powder
Adi	1:1 Acetonitrile/Methanol	Crystallization of known salt (ref code QUKHOV)
Adi	Water	Poor quality crystals
Adi	Acetone	Powder
Adi	Ethyl Acetate	Powder
Pim	Methanol	Crystallization of $(cip^{+})(pim^{-})$
Pim	Ethanol	Crystallization of $(cip^{+})(pim^{-})$
Pim	Acetonitrile	Powder
Pim	1:1 Acetonitrile/Methanol	Crystallization of $(cip^{+})_2(pim^{2-})\cdot H_2O$
Pim	Water	Crystallization of $(cip^{+})(pim^{-})$
Pim	Acetone	Powder
Pim	Ethyl Acetate	Powder
Sub	Methanol	Crystallization of $(cip^{+})_2(sub^{2-})\cdot 4H_2O$
Sub	Ethanol	Crystallization of $(cip^{+})_2(sub^{2-})\cdot 4H_2O$

Sub	Acetonitrile	Powder
Sub	1:1 Acetonitrile/Methanol	Poor quality crystals
Sub	Water	Crystallization of $(cip^+)_2(sub^{2-})\cdot 4H_2O$
Az	Methanol	Powder
Az	Ethanol	Powder
Az	Acetonitrile	Crystallization of $(cip^+)(az^-)\cdot CH_3CN$
Az	1:1 Acetonitrile/Methanol	Powder
Az	Water	Powder
Az	Acetone	Poor quality crystals
Az	Toluene	Powder
Seb	Methanol	Powder
Seb	Acetonitrile	Poor quality crystals
Seb	1:1 Acetonitrile/Methanol	Powder
Seb	Water	Poor quality crystals

Table S4. Crystallographic data of the supramolecular salts of enro.

Compound	**(enro$^+$)(glu$^-$) ·0.33CH$_3$CN·0.67H$_2$O**	**(enro$^+$)$_2$(adi^{2-}) ·adi·2CH$_3$CN**	**(enro$^+$)$_2$(pim^{2-}) ·1.5H$_2$O**	**(enro$^+$)(pim$^-$) ·H$_2$O**	**(enro$^+$)(pim$^-$) ·3H$_2$O**	**(enro$^+$)(sub$^-$) ·H$_2$O**	**(enro$^+$)(az$^-$)**	**(enro$^+$)(seb$^-$)**
Formula	$C_{74}H_{93}F_3N_{10}O_{23}$	$C_{27}H_{35}FN_4O_7$	$C_{22.5}H_{28}FN_3O_{5.75}$	$C_{26}H_{36}FN_3O_8$	$C_{26}H_{40}FN_3O_{10}$	$C_{27}H_{36}FN_3O_8$	$C_{28}H_{38}FN_3O_7$	$C_{58}H_{80}F_2N_6O_{14}$
M_r	1547.58	546.59	451.48	537.58	573.61	549.59	547.61	1123.28
Habit	Plate	Plate	Block	Block	Needle	Needle	Block	Plate
Crystal size (mm)	0.6 x 0.5 x 0.1	0.3 x 0.3 x 0.1	0.5 x 0.45 x 0.25	0.8 x 0.6 x 0.4	0.8 x 0.15 x 0.1	0.4 x 0.1 x 0.1	0.8 x 0.7 x 0.4	0.5 x 0.3 x 0.1
Crystal system	Triclinic	Triclinic	Monoclinic	Triclinic	Monoclinic	Triclinic	Triclinic	Triclinic
Space group	P-1	P-1	C2/c	P-1	C2/c	P-1	P-1	P-1
Unit cell dimensions								
a [Å]	9.6423	7.0026	12.4883	7.6363	26.0155	7.0794	7.8836	13.5018
b [Å]	20.0015	9.7600	13.8372	11.9558	7.2556	9.3834	13.1102	14.0602
c [Å]	20.7763	21.9405	26.409	15.2337	29.1351	21.9918	13.5774	15.4916
α [°]	107.550	102.046	-	90.673	-	95.841	78.324	91.525
β [°]	94.599	91.811	91.064	101.762	94.345	91.412	83.147	99.744
γ [°]	94.758	107.468	-	100.700	-	105.585	83.569	100.296
V [Å^3]	3783.8	1391.73	4562.8	1336.15	5483.7	1397.81	1358.86	2846.9
Z	2	2	8	2	8	2	2	2
D_{calc} (g cm^{-3})	1.358	1.304	1.314	1.336	1.390	1.306	1.338	1.310
No. measd. reflections	27232	10057	14737	8632	10780	9156	11766	23415
no. unique reflections (R_{int})	13792 (0.0466)	5103 (0.0196)	4169 (0.0816)	4890 (0.0198)	5018 (0.0343)	5104 (0.025)	6221 (0.0149)	13084 (0.0356)
No. obs. reflections	6018	3374	2598	3212	3721	2635	4803	5787
Final R_1, wR_2 (obs. reflections)	0.0758, 0.1913	0.0615,0.1638	0.799, 0.2490	0.0788, 0.2053	0.0469, 0.1021	0.0751, 0.2212	0.0509, 0.1376	0.0572, 0.1025
Goodness-of-fit (obs. reflections)	0.960	0.993	0.906	1.274	1.072	1.015	1.037	0.938

Table S5. Crystallographic data of the supramolecular salts of nor (1/2).

Compound	**$(nor^+)_2(glu^{2-})$ $\cdot H_2O \cdot CH_3OH$**	**$(nor^+)_2(glu^{2-})$ $\cdot H_2O \cdot 0.75CH_3CN$**	**$(nor^+)(pim^-)$ $\cdot CH_3OH$**	**$(nor^+)_2(pim^{2-})$ $\cdot C_2H_5OH$**	**$(nor^+)(pim^-)$**	**$(nor^+)_2(sub^{2-})$ $\cdot CH_3OH$**	**$(nor^+)(sub^-)$ $\cdot 3H_2O$**	**$(nor^+)(sub^-)$**
Formula	$C_{76}H_{96}F_4N_{12}O_{23}$	$C_{77}H_{92.5}F_4N_{13.5}O_{24}$	$C_{48}H_{68}F_2N_6O_{16}$	$C_{41}H_{54}F_2N_6O_{11}$	$C_{46}H_{60}F_2N_6O_{14}$	$C_{84}H_{116}F_4N_{12}O_{24}$	$C_{24}H_{38}FN_3O_{10}$	$C_{24}H_{32}FN_3O_7$
M_r	1621.64	1667.14	1023.08	844.90	959.0	1753.88	547.57	493.52
Habit	Block	Block	Block	Plate	Block	Block	Block	Needle
Size (mm)	0.75 x 0.4 x 0.4	0.3 x 0.3 x 0.2	0.6 x 0.4 x 0.3	0.8 x 0.3 x 0.15	0.7 x 0.7 x 0.5	0.5 x 0.5 x 0.4	0.5 x 0.5 x 0.3	0.7 x 0.05 x 0.0[illegible]
Crystal system	Monoclinic	Triclinic	Orthorhombic	Triclinic	Triclinic	Triclinic	Triclinic	Triclinic
Space group	P21/c	P1	$Pca2_1$	P-1	P-1	P-1	P-1	P-1
Unit cell dimensions								
a [Å]	32.5998	6.9942	17.221	9.8226	11.5268	15.035	9.3132	10.0319
b [Å]	13.8206	9.433	6.8957	12.6939	14.5175	16.0126	11.4933	10.2407
c [Å]	17.3765	32.6585	42.523	17.2426	15.8561	18.2009	14.5910	12.1158
α [°]	-	90.929	-	90.388	63.498	89.882	91.758	101.200
β [°]	93.070	94.484	-	92.939	89.529	75.612	106.550	94.535
γ [°]	-	109.056	-	104.379	78.900	87.569	111.386	98.737
V [$Å^3$]	7817.7	2028.38	5049.6	2079.4	2321.0	4240.4	1378.09	1199.2
Z	4	1	8	2	2	2	2	2
D_{calc} (g cm^{-3})	1.378	1.365	1.346	1.349	1.372	1.374	1.320	1.367
No. measd. reflections	61477	14111	12255	13966	16048	29421	10990	7812
no. unique reflections (R_{int})	18760 (0.0554)	10209 (0.022)	6663 (0.0434)	7590 (0.0847)	8466 (0.0424)	15455 (0.0711)	6319 (0.0237)	4380 (0.1038)
No. obs. reflections	9987	4901	5140	3352	5145	8578	3663	1141
Final R_1, wR_2 (obs. reflections)	0.1057, 0.2481	0.076, 0.2136	0.0848, 0.2562	0.1339, 0.3515	0.0714, 0.1854	0.0711, 0.1742	0.0627, 0.1477	0.0577,0.0792
Goodness-of-fit (obs. reflections)	1.023	1.082	1.063	1.113	1.017	0.987	1.014	0.709

Table S6. Crystallographic data of the supramolecular salts of nor (2/2).

Compound	**$(nor^+)_2(az^{2-})·az·4H_2O$**	**$(nor^+)_2(seb^{2-})·4CH_3OH$**	**$(nor^+)(seb^-)nor·H_2O$**	**$(nor^+)_2(adi^{2-})·2H_2O$ form I**	**$(nor^+)_2(adi^{2-})·2H_2O$ form II**	**$(nor^+)_2(seb^{2-})·3H_2O$**
Formula	$C_{25}H_{39}FN_3O_9$.6	$C_{23}H_{35}FN_3O_7$	$C_{42}H_{55.5}F_2N_6O_{11}$	$C_{19}H_{27}FN_3O_7$	$C_{19}H_{25}FN_3O_6$	$C_{42}H_{54}F2N_6O_{16.50}$
M_r	554.19	484.54	858.42	428.43	410.42	944.91
Habit	Plate	Plate	Block	Block	Needle	Plate
Size (mm)	0.4 x 0.2 x 0.2	0.5 x 0.3 x 0.1	0.5 x 0.2 x 0.2	0.6 x 0.5 x 0.4	0.8 x 0.15 x 0.1	0.5 x 0.3 0.2
Crystal system	Monoclinic	Monoclinic	Triclinic	Triclinic	Triclinic	Triclinic
Space group	$P2_1/n$	C2/c	P-1	P-1	P-1	P-1
Unit cell dimensions						
a [Å]	9.3287	42.543	9.6354	7.2502	6.8861	7.4605
b [Å]	22.9608	6.8319	11.1605	11.2327	9.7466	11.2181
c [Å]	25.1702	17.295	21.3064	12.8744	15.4257	14.8638
α [°]	-	-	96.888	87.992	82.400	103.595
β [°]	90.289	97.421	98.719	75.520	86.148	99.497
γ [°]	-	-	111.392	87.474	69.953	92.348
V [$Å^3$]	5391.3	4987.7	2070.1	1013.88	963.78	1188.43
Z	8	8	2	2	2	1
D_{calc} (g cm^{-3})	1.366	1.291	1.377	1.403	1.414	1.320
No. measd. reflections	16452	16190	13468	6722	7179	7759
no. unique reflections (R_{int})	9750 (0.0236)	4556 (0.0507)	7548 (0.0468)	3715 (0.0185)	4393 (0.0315)	4325(0.0262)
No. obs. reflections	6665	2564	3072	2745	3065	3042
Final R_1, wR_2 (obs. reflections)	0.0584, 0.1469	0.0618, 0.1459	0.0996, 0.2166	0.0454 0.1096	0.0639, 0.1761	0.0591, 0.1700
Goodness-of-fit (obs. reflections)	0.947	1.013	1.038	0.999	0.984	0.935

Table S7. Crystallographic data of the supramolecular salts of cip.

Compound	$(cip^+)(glu^-)$	$(cip^+)(pim^-)$	$(cip^+)_2(pim^{2-})\cdot H_2O$	$(cip^+)_2(sub^{2-})\cdot 4H_2O$	$(cip^+)(az^-)\cdot CH_3CN$
Formula	$C_{44}H_{52}F_2N_6O_{14}$	$C_{24}H_{30}FN_3O_7$	$C_{20.5}H_{24}FN_3O_7$	$C_{21}H_{29}FN_3O_7$	$C_{28}H_{37}FN_4O_7$
M_r	926.91	491.51	443.43	454.47	560.61
Habit	Plate	Needle	Needle	Needle	Block
Size	0.3 x 0.2 x 0.2	0.4 x 0.25 x 0.2	0.8 x 0.5 x 0.2	0.75 x 0.15 x 0.05	0.4 x 0.4 x 0.3
Crystal system	Triclinic	Orthorhombic	Monoclinic	Triclinic	Triclinic
Space group	P-1	Pbca	C2/c	P-1	P-1
Unit cell dimensions					
a [Å]	7.1811	8.4398	24.9174	7.4801	9.9902
b [Å]	16.5398	23.4988	7.1174	11.4803	12.1238
c [Å]	18.8198	24.002	24.5887	13.2385	12.6545
α [°]	93.924	-	-	94.096	71.432
β [°]	99.728	-	95.082	102.254	82.750
γ [°]	90.242	-	-	95.417	78.192
V [Å^3]	2197.71	4760.2	4343.6	1101.08	1419.09
Z	2	8	8	2	2
D_{calc} (g cm^{-3})	1.401	1.372	1.356	1.371	1.312
No. measd. reflections	15549	16697	14898	6827	11555
no. unique reflections (R_{int})	8047 (0.0530)	16697 (0.1218)	3977 (0.0237)	4008 (0.0192)	6591 (0.0192)
No. obs. reflections	3185	7570	2987	2855	4149
Final R_1, wR_2 (obs. reflections)	0.0568, 0.0850	0.1137, 0.2069	0.0747, 0.2041	0.0481, 0.1197	0.0540, 0.1187
Goodness-of-fit (obs. reflections)	0.893	1.022	1.016	0.929	0.993

Table S8. Crystallographic data of the enro solvates.

Compound	enro·CH_3CN	enro·H_2O
Formula	$C_{21}H_{25}FN_4O_3$	$C_{19}H_{24}FN_3O_4$
M_r	400.45	377.41
Crystal system	Monoclinic	Monoclinic
Space group	$P2_1/c$	$P2_1/n$
Unit cell dimensions		
a [Å]	13.6993	9.988
b [Å]	7.7470	7.0869
c [Å]	19.2496	25.637
α [°]	90	90
β [°]	103.303	95.35
γ [°]	90	90
V [Å^3]	1988.1	1806.7
Z	4	4
D_{calc} (g cm^{-3})	1.338	1.388
No. measd. reflections	16614	15082
no. unique reflections (R_{int})	4831 (0.0459)	4351 (0.0849)
No. obs. reflections	3155	1644
Final R_1, wR_2 (obs. reflections)	0.0602, 0.1517	0.0753, 0.1294
Goodness-of-fit (obs. reflections)	1.044	0.926

Table S9. H bonding interactions in the salts of nor, cip and enro and carboxylic acids HOOC-$(CH_2)_n$-COOH (n = 3 – 8).

	D-H···A	d(D···A) (Å)	∠(DHA) (°)	symmetry code
$(enro^+)(glu^-)\cdot0.33CH_3CN\cdot0.67H_2O$	N(1)-H(1N1)...O(4)	2.710(4)	167(4)	
	N(7)-H(1N7)...O(11)	2.699(4)	163(5)	
	N(7)-H(1N7)...O(12)	3.190(6)	138(4)	
	N(4)-H(1N4)...O(6)	2.710(4)	168(3)	
	O(9)-H(1O9)...O(6)	2.543(4)	175(5)	
	O(8)-H(1O8)...O(11)	2.541(4)	164(6)	
	O(20)-H(1O3)...O(21)	2.545(5)	156(5)	
	O(17)-H(1O1)...O(18)	2.558(5)	152(5)	
	O(14)-H(1O4)...O(15)	2.550(5)	160(5)	
	O(2)-H(1O2)...O(4)	2.539(5)	161(6)	x-1,-y+2,-z
$(enro^+)_2(adi^{2-})\cdot adi\cdot2CH_3CN$	O(1)-H(1)...O(3)	2.519(3)	153.1	
	O(5)-H(1O5)...O(6)	2.558(3)	164(4)	
	N(1)-H(1N1)...O(6)	2.640(3)	170(4)	
	N(1)-H(1N1)...O(7)	3.255(3)	132(3)	
$(enro^+)_2(pim^{2-})\cdot1.5H_2O$	O(2)-H(1O2)...O(3)	2.533(4)	156(6)	
	N(3)-H(1N3)...O(4)	2.592(4)	170(8)	
	N(3)-H(1N3)...O(5)	3.249(4)	131(6)	
$(enro^+)(pim^-)\cdot H_2O$	O(4)-H(4)...O(7)	2.633(5)	150.6	-x+1,-y+1,-z
	N(1)-H(1N1)...O(6)	2.592(5)	171(4)	
	N(1)-H(1N1)...O(7)	3.152(5)	126(3)	
	O(2)-H(1O2)...O(3)	2.530(4)	148(5)	
	O(8)-H(1O8)...O(1)	2.962(4)	171(8)	-x+1,-y+1,-z+1
	O(8)-H(2O8)...O(6)	2.793(5)	164(3)	
$(enro^+)(pim^-)\cdot3H_2O$	O(8)-H(1O8)...O(5)	2.926(2)	168(3)	-x+2,-y+1,-z+1
	O(8)-H(2O8)...O(5)	2.792(2)	178(3)	
	O(10)-H(1O1)...O(9)	2.867(3)	178(4)	
	O(10)-H(2O1)...O(4)	2.932(2)	171(3)	x-1/2,y+1/2,z
	O(9)-H(1O9)...O(1)	2.755(2)	163(3)	
	O(9)-H(2O9)...O(8)	2.812(2)	177(3)	-x+3/2,-y+1/2,-z+1
	N(1)-H(1N1)...O(1)	2.703(2)	172(2)	
	O(6)-H(1O6)...O(7)	2.480(2)	158(3)	
	O(4)-H(1O4)...O(2)	2.502(2)	178(3)	-x+3/2,y-1/2,-z+3/2
	O(2)-H(2)...O(3)	2.544(5)	146.2	
$(enro^+)(sub^-)\cdot H_2O$	N(1)-H(1N1)...O(4)	2.658(4)	175(3)	
	O(7)-H(1O7)...O(4)	2.555(4)	168(5)	x+1,y+1,z
$(enro^+)(az^-)$	N(1)-H(1N1)...O(5)	2.6391(18)	172(2)	
	N(1)-H(1N1)...O(4)	3.318(2)	129.9(17)	
	O(2)-H(1O2)...O(3)	2.5252(17)	162(3)	
	O(7)-H(1O7)...O(4)	2.542(2)	172(3)	-x+2,-y+2,-z+1

$(enro^{+})(seb^{-})$	N(4)-H(1N4)...O(3)	3.197(3)	121(2)	
	N(4)-H(1N4)...O(4)	2.635(3)	178(3)	
	O(1)-H(1O1)...O(4)	2.599(3)	169(3)	x-1,y,z
	N(1)-H(1N1)...O(5)	2.622(3)	170(3)	
	N(1)-H(1N1)...O(6)	3.067(3)	128(2)	
	O(10)-H(1O2)...O(11)	2.540(2)	157(3)	
	O(13)-H(1O3)...O(14)	2.550(3)	153(3)	
	O(8)-H(1O8)...O(6)	2.509(3)	168(3)	x+1,y,z
$(nor^{+})_2(glu^{2-})\cdot H_2O\cdot CH_3OH$	O(22)-H(22)...O(9)	2.789(6)	167.8	
	O(23)-H(23)...O(8)	2.719(6)	161.5	
	C(71)-H(71)...O(13)	3.320(5)	146.6	-x+2,-y+1,-z+2
	O(2)-H(1O2)...O(3)	2.511(4)	150(5)	
	O(12)-H(1O1)...O(11)	2.514(4)	148(7)	
	O(15)-H(1O3)...O(14)	2.505(4)	142(5)	
	N(3)-H(1N3)...O(8)	2.713(5)	178(5)	
	N(3)-H(2N3)...O(20)	2.749(6)	174(5)	x,y,z-1
	N(12)-H(1N2)...O(19)	2.687(5)	164(4)	x,y,z-1
	N(12)-H(2N2)...O(7)	2.693(5)	166(5)	
	N(7)-H(1N7)...O(9)	2.677(5)	161(6)	
	N(7)-H(2N7)...O(17)	2.700(7)	175(5)	
	O(5)-H(1O5)...O(6)	2.518(4)	151(5)	
	N(6)-H(1N6)...O(18)	2.703(6)	166(7)	
	N(6)-H(2N6)...O(10)	2.722(6)	176(8)	
	O(2)-H(2)...O(3)	2.515(9)	146.9	
$(nor^{+})_2(glu^{2-})\cdot H_2O\cdot 0.75CH_3CN$	O(8)-H(8)...O(9)	2.510(10)	153.4	
	O(11)-H(11)...O(12)	2.519(10)	148.0	
	N(3)-H(3A)...O(18)	2.590(11)	170.3	
	N(3)-H(3B)...O(22)	2.693(12)	171.0	x-1,y-1,z
	N(4)-H(4B)...O(17)	2.678(11)	171.8	
	N(4)-H(4A)...O(20)	2.786(10)	151.3	x-1,y-1,z
	N(7)-H(7A)...O(23)	2.745(12)	174.3	x-1,y-1,z
	N(7)-H(7B)...O(16)	2.784(14)	169.3	
	N(10)-H(10A)...O(15)	2.725(12)	172.3	
	N(10)-H(10B)...O(13)	2.688(10)	147.4	x-1,y-1,z
	N(10)-H(10B)...O(14)	3.487(15)	152.1	x-1,y-1,z
$(nor^{+})(pim^{-})\cdot CH_3OH$	O(1)-H(1)...O(3)	2.498(8)	154.1	
	O(14)-H(14)...O(11)#1	2.501(10)	140.4	x-1/2,-y,z
	N(4)-H(4A)...O(13)#2	2.780(11)	160.5	x+1/2,-y+1,z
	N(4)-H(4B)...O(11)	2.691(10)	155.3	
	N(4)-H(4B)...O(12)	3.362(13)	146.8	
	O(2X)-H(2X)...O(5)	2.650(11)	143.8	
	O(1X)-H(1X)...O(12)	2.708(13)	158.5	
	N(1)-H(1N1)...O(4)	2.661(11)	131(10)	
	N(1)-H(1N1)...O(5)	3.365(11)	161(9)	
	N(1)-H(2N1)...O(6)#7	2.808(11)	152(9)	x-1/2,-y+1,z
	O(7)-H(1O7)...O(4)#9	2.537(9)	162(11)	x+1/2,-y,z
	O(8)-H(1O8)...O(10)	2.512(8)	149(11)	
$(nor^{+})_2(pim^{2-})\cdot C_2H_5OH$	O(2)-H(2)...O(3)	2.487(6)	155.0	

	O(9)-H(9)...O(10)	2.530(5)	153.3	
	N(3)-H(3BX)...O(4)	2.708(6)	172.1	x,y-1,z
	N(3)-H(3BX)...O(5A)	3.30(3)	133.4	x,y-1,z
	N(4)-H(4AX)...O(6)	2.612(6)	169.3	x,y+1,z
	N(4)-H(4BX)...O(4)	2.668(7)	165.7	
$(nor^+)(pim^-)$	O(2)-H(1O2)...O(3)	2.539(3)	156(6)	
	O(2)-H(1O2)...O(14)	2.969(3)	110(4)	x+1,y-2,z+1
	O(13)-H(1O3)...O(14)	2.534(3)	167(5)	
	N(3)-H(1N3)...O(4)	2.668(3)	177(3)	
	N(3)-H(2N3)...O(9)	2.701(4)	170(4)	-x+1,-y+1,-z+1
	N(4)-H(1N4)...O(5)	2.873(4)	129(3)	
	N(4)-H(1N4)...O(6)	2.943(4)	132(3)	-x+1,-y+1,-z+1
	N(4)-H(2N4)...O(8)	2.897(4)	134(3)	-x+1,-y+1,-z+1
	N(4)-H(2N4)...O(11)	2.999(4)	128(3)	x-1,y-1,z+1
$(nor^+)_2(sub^{2-})\cdot CH_3OH$	O(23)-H(23)...O(11)	2.785(4)	169.6	
	O(22)-H(22)...O(5)	2.714(4)	165.0	
	O(21)-H(21)...N(3)	3.123(5)	139.1	
	O(24)-H(24)...N(5)	3.208(5)	135.4	
	N(3)-H(1N3)...O(7)	2.695(3)	159(4)	
	N(3)-H(1N3)...O(21)	3.123(5)	118(3)	
	N(3)-H(2N3)...O(5)	2.614(4)	159(3)	
	N(5)-H(1N5)...O(11)	2.651(3)	161(4)	
	N(5)-H(2N5)...O(13)	2.602(4)	155(4)	
	N(8)-H(1N8)...O(6)	2.628(4)	167(4)	
	N(8)-H(2N8)...O(4)	2.788(3)	161(3)	
	N(8)-H(2N8)...O(23)	3.064(4)	111(3)	-x+1,-y+1,-z+1
	N(11)-H(1N1)...O(12)	2.711(4)	149(3)	
	N(11)-H(1N1)...O(22)	3.173(4)	120(3)	-x+2,-y+1,-z+1
	N(11)-H(2N2)...O(14)	2.665(4)	152(5)	
	O(8)-H(1O8)...O(10)	2.568(4)	154(6)	
	O(2)-H(1O2)...O(3)	2.535(4)	156(5)	
	O(19)-H(1O1)...O(20)	2.556(4)	155(4)	
	O(17)-H(1O7)...O(16)	2.520(3)	165(7)	
$(nor^+)(sub^-)\cdot 3H_2O$	O(6)-H(6)...O(5)	2.509(2)	169.7	x,y-1,z
	N(3)-H(1N3)...O(9)	2.832(3)	166(3)	-x+2,-y+2,-z+1
	N(3)-H(2N3)...O(4)	2.735(3)	162(2)	
	O(2)-H(1O2)...O(3)	2.541(2)	158(4)	
	O(2)-H(1O2)...O(3)	3.052(2)	114(3)	-x-1,-y+2,-z
	O(10)-H(1O1)...O(4)	2.791(3)	177(5)	
	O(10)-H(2O1)...O(8)	3.115(5)	158(4)	-x+2,-y+1,-z+1
	O(9)-H(1O9)...O(5)	2.745(3)	176(3)	
	O(9)-H(2O9)...O(8)	2.909(4)	168(4)	-x+3,-y+1,-z+1
	O(8)-H(2O8)...O(7)	2.790(3)	169(5)	
	O(8)-H(1O8)...O(10)	2.840(4)	158(4)	x+1,y,z
$(nor^+)(sub^-)$	N(1)-H(1N1)...O(4)	2.617(7)	162(6)	
	N(1)-H(2N1)...O(5)#6	2.854(6)	173(6)	x-1,y-1,z
	O(6)-H(1O6)...O(7)	2.507(6)	157(9)	
	O(1)-H(1O1)...O(4)#6	2.526(4)	169(6)	x-1,y-1,z

$(nor^+)_2(az^{2-})\cdot az\cdot 4H_2O$	N(3)-H(3A)...O(4)	2.750(3)	172.6	
	N(3)-H(3B)...O(7)	2.784(3)	162.2	-x+1/2,y-1/2,-z+1/2
	N(6)-H(6A)...O(9)	2.764(3)	174.3	-x+3/2,y+1/2,-z+1/2
	N(6)-H(6B)...O(11)	2.759(3)	159.1	
	O(19)-H(19A)...O(4)	2.804(3)	178(4)	
	O(19)-H(19B)...O(9)	2.831(3)	179(4)	-x-1/2,y-1/2,-z+1/2
	O(5)-H(1O5)...O(10)	2.471(2)	176(6)	x-1,y-1,z
	O(17)-H(17A)...O(7)	2.793(3)	173(4)	
	O(18)-H(18A)...O(19)	2.888(3)	168(4)	x+1,y,z
	O(18)-H(18B)...O(17)	2.867(4)	163(4)	
	O(15)-H(15C)...O(11)	2.808(3)	173(4)	
	O(15)-H(15D)...O(3)	2.866(4)	171(4)	x+5/2,-y+1/2,z-1/2
	O(16)-H(16C)...O(15)	2.797(4)	176(5)	
	O(16)-H(16D)...O(19)	2.880(4)	172(4)	-x+1/2,y+1/2,-z+1/2
	O(6)-H(6C)...O(8)	2.463(3)	170(6)	
	O(6)-H(6C)...O(9)	3.124(3)	127(5)	
	O(2)-H(2A)...O(12)	3.158(3)	113(3)	x-7/2,-y+1/2,z+1/2
	O(2)-H(2A)...O(1)	2.507(3)	161(4)	
	O(14)-H(14C)...O(12)	2.502(3)	158(5)	
	O(17)-H(17B)...O(13)	2.951(4)	179(5)	-x+3,-y+1,-z
$(nor^+)_2(seb^{2-})\cdot 4CH_3OH$	O(6)-H(6)...O(4)	2.751(4)	167.3	
	O(7)-H(7)...O(5)	2.673(4)	170.2	
	N(1)-H(1N1)...O(4)	2.726(4)	172(3)	
	N(1)-H(1N1)...O(5)	3.232(4)	128(2)	
	O(2)-H(1O2)...O(3)	2.504(3)	154(4)	
	N(1)-H(2N1)...O(7)	2.743(4)	173(3)	x,y-1,z
$(nor^+)(seb^-)\cdot nor\cdot H_2O$	O(11)-H(1O8)...O(2)	2.935(7)	171(8)	-x,-y+1,-z
	O(11)-H(2O8)...O(7)	2.939(8)	167(8)	
	O(2)-H(1O2)...O(3)	2.482(5)	155(6)	
	N(4)-H(1N4)...O(6)	3.250(6)	130(7)	
	N(4)-H(1N4)...O(7)	2.645(6)	173(8)	
	N(4)-H(2N4)...O(6)	2.774(8)	150(6)	-x+3,-y+1,-z+1
	O(9)-H(1O9)...O(8)	2.508(6)	160(9)	
	N(3)-H(1N3)...O(4)	3.211(6)	154(6)	
	N(3)-H(1N3)...O(5)	2.895(6)	148(6)	
	O(4)-H(1O4)...O(4)	2.420(9)	180.0(4)	-x+1,-y+1,-z
$(nor^+)_2(adi^{2-})\cdot 2H_2O$, form I	N(3)-H(1N3)...O(5)	2.688(2)	160(2)	
	N(3)-H(2N3)...O(7)	2.760(2)	172(2)	-x+1,-y+1,-z
	O(6)-H(1O6)...O(1)	2.980(2)	170(3)	-x+1,-y,-z+1
	O(6)-H(2O6)...O(5)	2.764(2)	170(3)	
	O(7)-H(1O7)...O(4)	2.695(2)	174(3)	
	O(7)-H(2O7)...O(5)	3.274(3)	148(3)	-x+2,-y+1,-z
	O(7)-H(2O7)...O(6)	3.140(3)	142(3)	-x+2,-y+1,-z
$(nor^+)_2(adi^{2-})\cdot 2H_2O$, form II	O(2)-H(1O2)...O(3)	2.524(3)	149(4)	
	N(1)-H(1N1)...O(4)	2.724(3)	175(3)	
	N(1)-H(2N1)...O(4)	3.082(3)	121(2)	-x+1,-y+2,-z+2
	N(1)-H(2N1)...O(5)	2.737(3)	170(3)	-x+1,-y+2,-z+2

	O(6)-H(1O6)...O(4)	2.815(3)	175(4)	
	O(6)-H(2O6)...O(5)	2.904(3)	159(4)	x-1,y,z
$(nor^{+})_2(seb^{2-})\cdot 3H_2O$	N(2)-H(1N2)...O(7A)	2.820(5)	168(3)	
	N(2)-H(1N2)...O(7B)	2.676(6)	159(3)	
	N(2)-H(2N2)...O(4)	2.676(3)	169(3)	
	O(2)-H(1O2)...O(3)	2.519(3)	156(4)	
$(cip^{+})(glu^{-})$	O(7)-H(1O7)...O(9)	2.521(5)	172(4)	
	O(12)-H(1O8)...O(14)	2.526(5)	165(5)	
	O(2)-H(1O2)...O(3)	2.531(4)	155(4)	
	O(5)-H(1O5)...O(6)	2.521(4)	149(4)	
	N(6)-H(1N6)...O(13)	2.712(5)	179(6)	
	N(6)-H(2N6)...O(13)	2.968(5)	155(3)	-x+3,-y+1,-z+1
	N(6)-H(2N6)...O(14)	2.910(5)	142(3)	-x+3,-y+1,-z+1
	N(3)-H(1N3)...O(10)	2.715(4)	173(3)	
	N(3)-H(2N3)...O(10)	2.776(4)	159(3)	-x,-y+1,-z
$(cip^{+})(pim^{-})$	O(7)-H(7A)...O(5)	2.425(6)	165.9	-x+5/2,y-1/2,z
	O(1)-H(1O1)...O(3)	2.536(8)	158(8)	
	N(3)-H(1N3)...O(4)	2.810(7)	168(6)	
	N(3)-H(2N3)...O(6)#7	2.809(8)	170(7)	-x+2,y+1/2,-z+1/2
$(cip^{+})_2(pim^{2-})\cdot H_2O$	N(1)-H(1N1)...O(1)	2.716(4)	171(4)	
	N(1)-H(2N1)...O(1)	2.726(3)	175(4)	-x+1/2,y-1/2,-z+1/2
	N(1)-H(2N1)...O(2)	3.319(4)	129(3)	-x+1/2,y-1/2,-z+1/2
	O(4)-H(1O4)...O(3)	2.507(3)	156(5)	
$(cip^{+})_2(sub^{2-})\cdot 4H_2O$	O(6)-H(1O6)...O(2)	2.774(3)	159(3)	
	O(6)-H(2O6)...O(3)	2.941(3)	166(4)	-x+1,-y,-z+1
	O(7)-H(1O7)...O(1)	2.668(3)	167(3)	
	O(7)-H(2O7)...O(6)	2.802(3)	161(3)	-x,-y+1,-z
	N(3)-H(1N3)...O(1)	2.618(2)	168(3)	
	N(3)-H(2N3)...O(7)	2.686(3)	177(3)	-x+1,-y+1,-z
	O(4)-H(1O4)...O(5)	2.521(2)	153(3)	
$(cip^{+})(az^{-})\cdot CH_3CN$	N(3)-H(1N3)...O(5)	2.737(3)	161.9(19)	
	N(3)-H(2N3)...O(6)	2.823(2)	160(2)	x,y+1,z
	N(3)-H(2N3)...O(7)	3.017(2)	133(2)	x,y+1,z
	O(2)-H(1O2)...O(1)	2.519(2)	155(3)	
	O(6)-H(1O6)...O(4)	2.483(2)	171(4)	-x+1,-y+1,-z+1
	O(6)-H(1O6)...O(5)	3.150(2)	117(3)	-x+1,-y+1,-z+1

Table S10. Results of the screening of the fluoroquinolone salts for gelator properties by heating followed by slow cooling to room temperature (salt : solvent = 1 : 10 (w/w)).

Co-former	Solvent	Norfloxacin		Ciprofloxacin		Enrofloxacin	
		1:1	2:1	1:1	2:1	1:1	2:1
**Glu**	H_2O	precipitate	precipitate	precipitate	insoluble	precipitate	precipitate
	ethanol	insoluble	insoluble	insoluble	insoluble	insoluble	precipitate
	PG	gelatinous ppt	gel (8 – 10 %)	gel (6 – 10 %)	gelatinous ppt	precipitate	gel (10 %)
	PEG	insoluble	insoluble	insoluble	insoluble	gelatinous ppt	gelatinous ppt
	methanol	precipitate		insoluble		gel (7 – 10 %)	
	CH_3OH/CH_3CN	precipitate		insoluble		precipitate	
	acetonitrile	precipitate		insoluble		precipitate	
	ethyl acetate	insoluble		insoluble		precipitate	
**Adi**	H_2O	precipitate	precipitate	gel (5 – 10 %)	insoluble	precipitate	precipitate
	ethanol	insoluble	insoluble	insoluble	insoluble	precipitate	precipitate
	PG	gel (9 – 10 %)	gel (10 %)	precipitate	precipitate	precipitate	gel (10 %)
	PEG	insoluble	insoluble	insoluble	precipitate	gelatinous ppt	gel (5-6%)
	methanol	insoluble		insoluble		precipitate	
	CH_3OH/CH_3CN	insoluble		insoluble		precipitate	
	acetonitrile	insoluble		insoluble		gelatinous ppt	
	ethyl acetate	insoluble		insoluble		insoluble	
**Pim**	H_2O	precipitate	gel (10 %)	precipitate	insoluble	precipitate	precipitate
	ethanol	insoluble	precipitate	insoluble	Insoluble	gelatinous ppt	precipitate
	PG	gelatinous ppt	precipitate	gelatinous ppt	precipitate	gel (10 %)	gel (10 %)
	PEG	insoluble	insoluble	insoluble	precipitate	gelatinous ppt	gelatinous ppt
	methanol	precipitate		insoluble		precipitate	
	CH_3OH/CH_3CN	precipitate		insoluble		precipitate	
	acetonitrile	insoluble		insoluble		gelatinous ppt	
	ethyl acetate	insoluble		insoluble		insoluble	

**Sub**	H_2O	precipitate	precipitate	precipitate	insoluble	gel (10 %)	insoluble
	ethanol	insoluble	insoluble	insoluble	Insoluble	gelatinous ppt	precipitate
	PG	gelatinous ppt	precipitate	gel (4 – 10 %)	gelatinous ppt	gel (8 – 10 %)	gelatinous ppt
	PEG	insoluble	insoluble	insoluble	precipitate	gel (6 – 10 %)	gel (4-6%)
	methanol	insoluble		insoluble		gelatinous ppt	
	CH_3OH/CH_3CN	precipitate		insoluble		precipitate	
	acetonitrile	insoluble		insoluble		precipitate	
	ethyl acetate	insoluble		insoluble		insoluble	
**Az**	H_2O	precipitate	precipitate	precipitate	insoluble	precipitate	precipitate
	ethanol	precipitate	insoluble	insoluble	insoluble	precipitate	precipitate
	PG	gel (7 – 17 %)	precipitate	precipitate	gelatinous ppt	gelatinous ppt	gel (10 %)
	PEG	insoluble	insoluble	insoluble	precipitate	gelatinous ppt	gel (5%)
	methanol	precipitate		insoluble		precipitate	
	CH_3OH/CH_3CN	insoluble		insoluble		precipitate	
	acetonitrile	insoluble		insoluble		precipitate	
	ethyl acetate	insoluble		insoluble		precipitate	
**Seb**	H_2O	precipitate	gelatinous ppt	gel (5 – 7 %)	insoluble	gel (7 – 10 %)	gel (5 – 10 %)
	ethanol	precipitate	insoluble	insoluble	insoluble	gelatinous ppt	precipitate
	PG	precipitate	precipitate	precipitate	gelatinous ppt	precipitate	gel (10 %)
	PEG	insoluble	insoluble	insoluble	insoluble	gel (7 – 10 %)	gel (4-8%)
	methanol	precipitate		insoluble		precipitate	
	CH_3OH/CH_3CN	precipitate		insoluble		precipitate	
	acetonitrile	insoluble		insoluble		precipitate	
	ethyl acetate	insoluble		insoluble		precipitate	

(a)

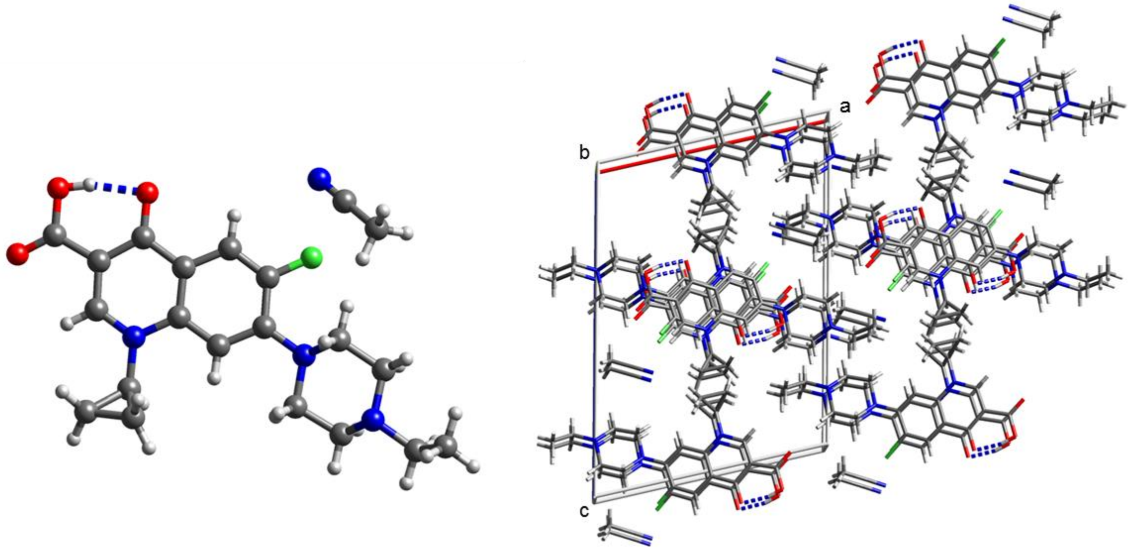

(b)

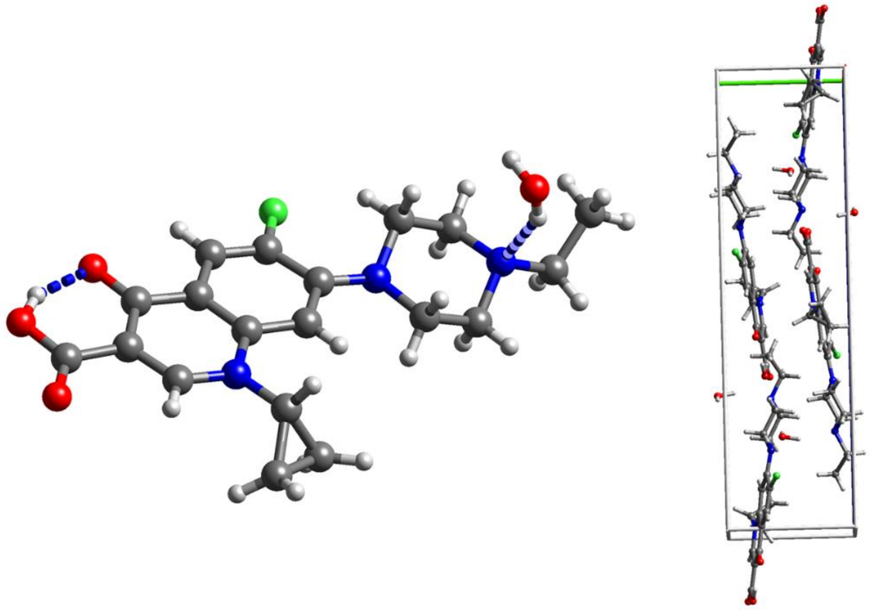

Figure S1. Crystal structures of (a) enro·CH_3CN and (b) enro·H_2O.

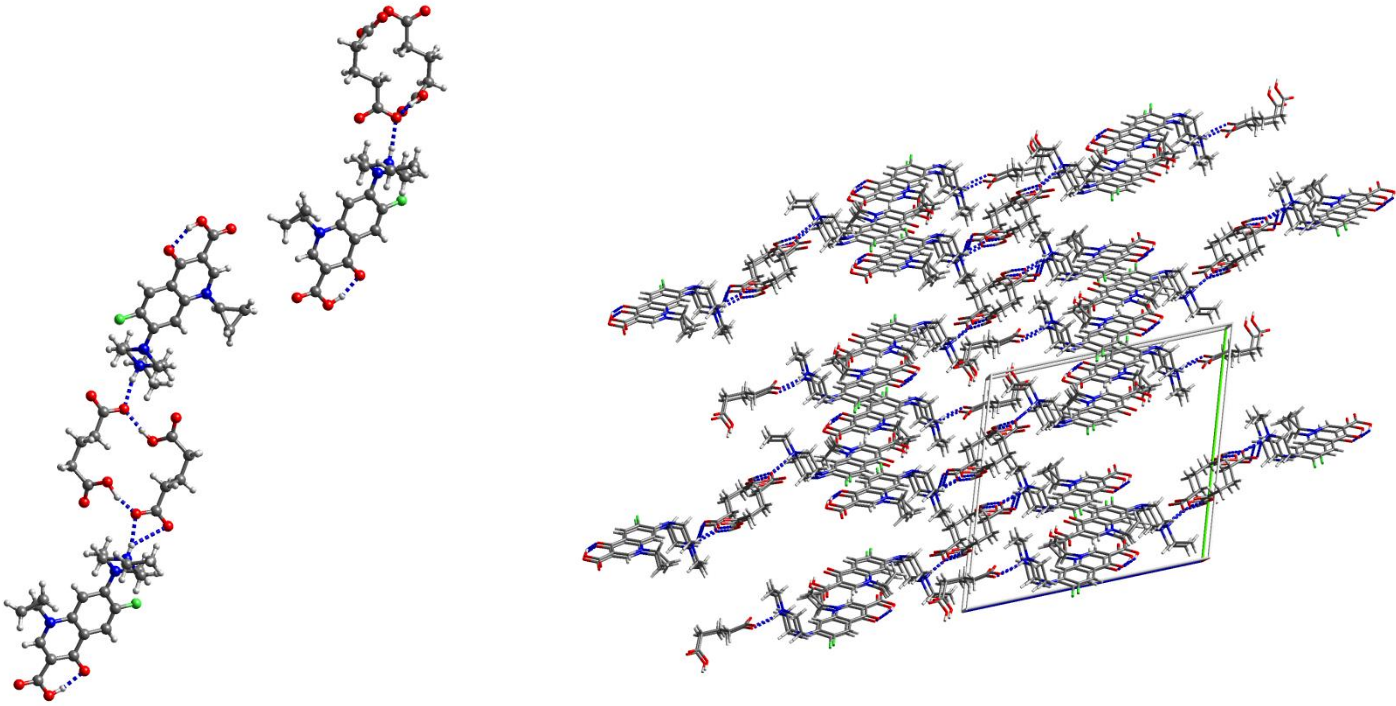

Figure S2. H bonding motif and crystal packing of (enro$^+$)(glu$^-$)·0.33CH_3CN·0.67H_2O. The asymmetric unit contains three enro$^+$ cations, three glu$^-$ anions, one acetonitrile molecule and two water molecules of crystallization. The solvent and water molecules of crystallization are not shown for clarity.

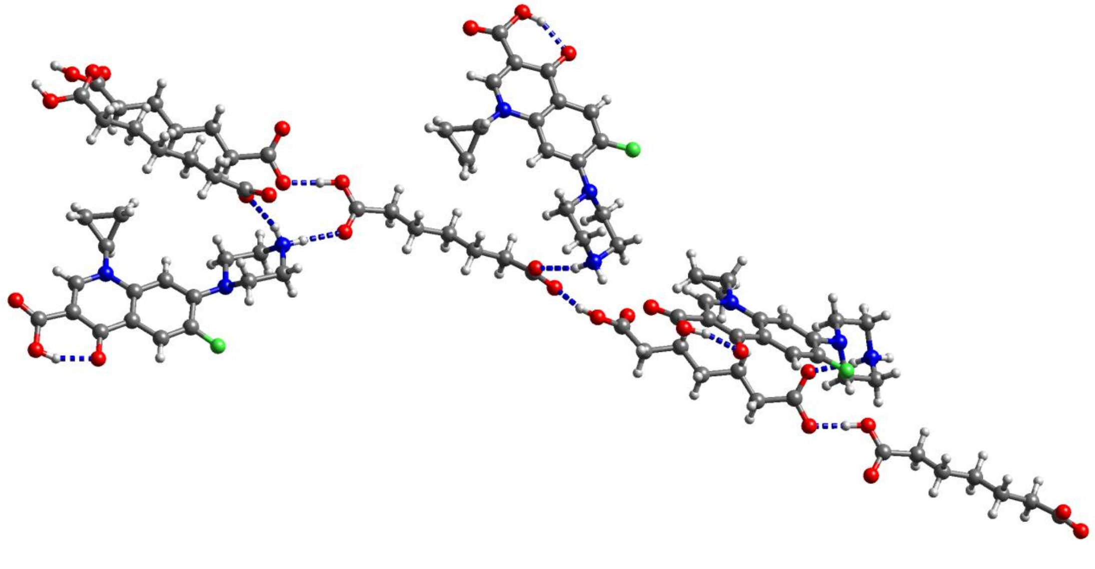

Figure S3. H bonding motif in (cip$^+$)(pim$^-$).

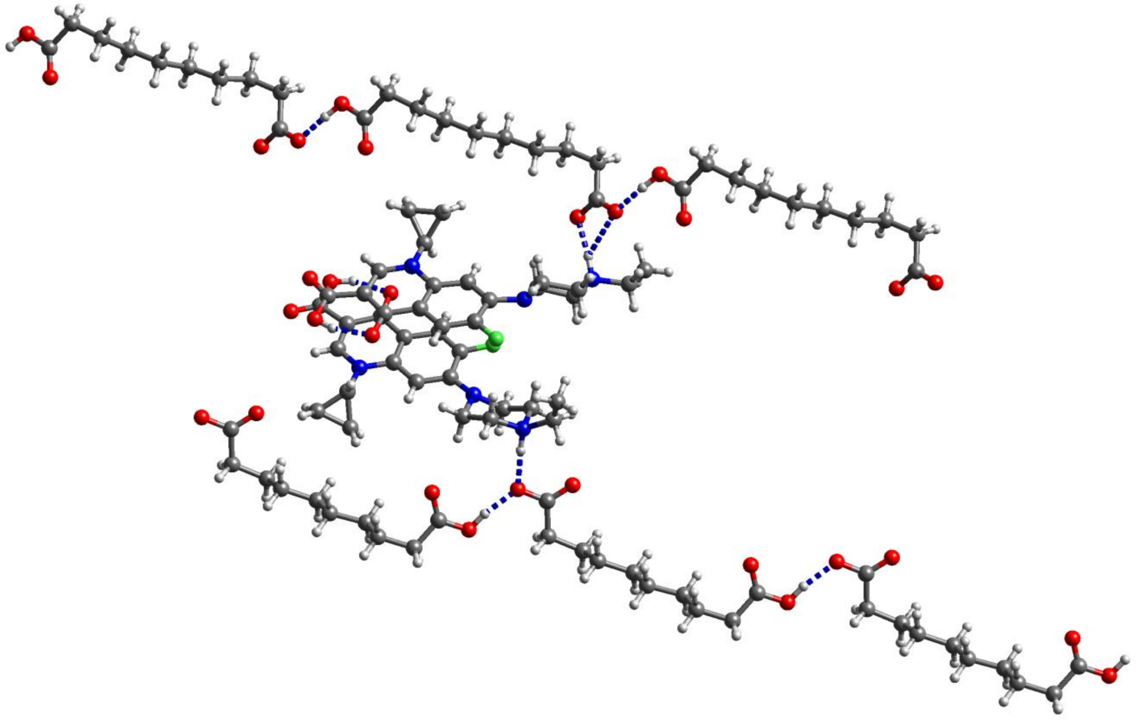

Figure S4. H bonding motif in (enro$^+$)(seb$^-$). For clarity, only one component of the disordered ethyl group of enro$^+$ is shown.

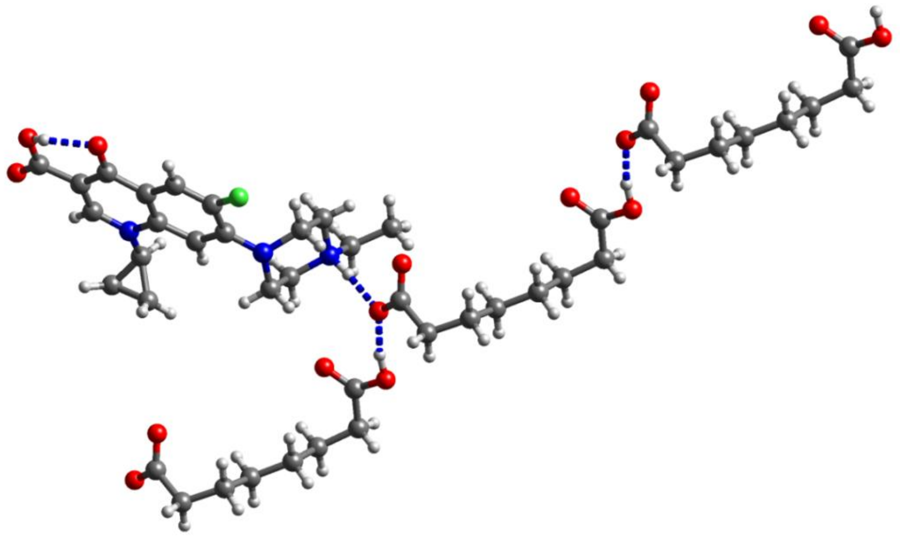

Figure S5. H bonding motif in (enro$^+$)(sub$^-$)·H_2O. For clarity, the water molecule of crystallization is not shown.

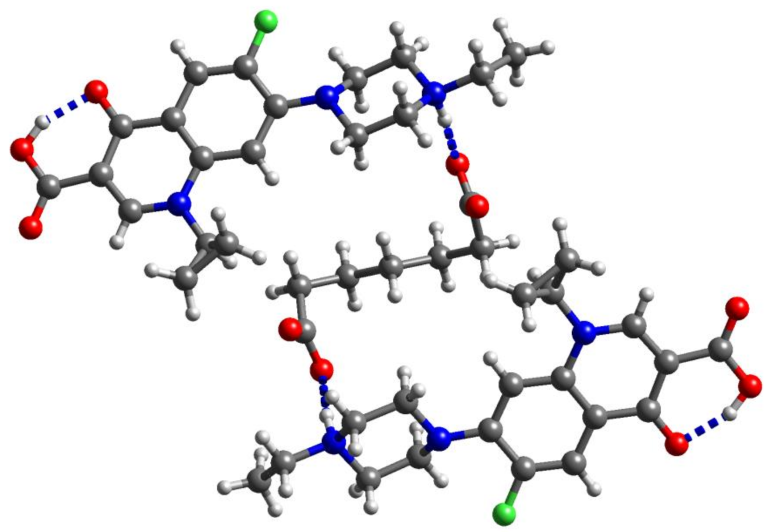

Figure S6. H bonding motif in $(enro^{+})_2(pim^{2-})\cdot 1.5H_2O$. The water molecule of crystallization is not shown.

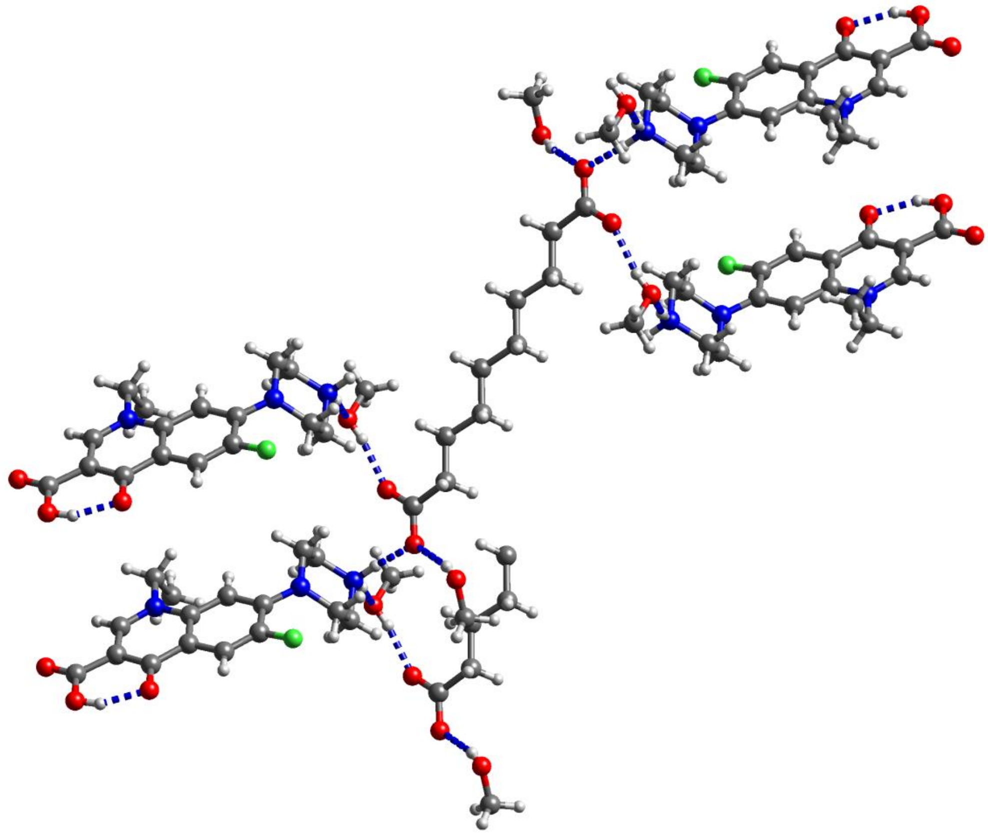

Figure S7. H bonding motif in $(nor^{+})_2(seb^{2-})\cdot 4CH_3OH$.

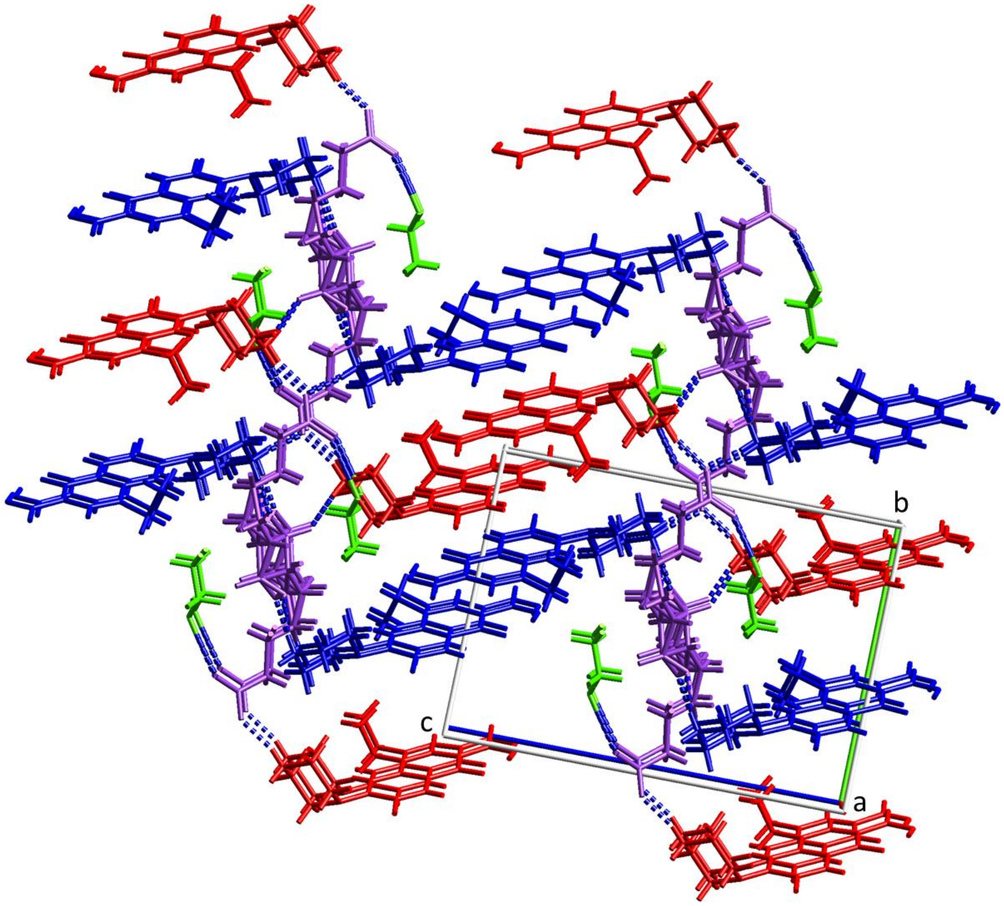

Figure S8. Crystal packing of $(nor^{+})_2(pim^{2-})\cdot C_2H_5OH$.

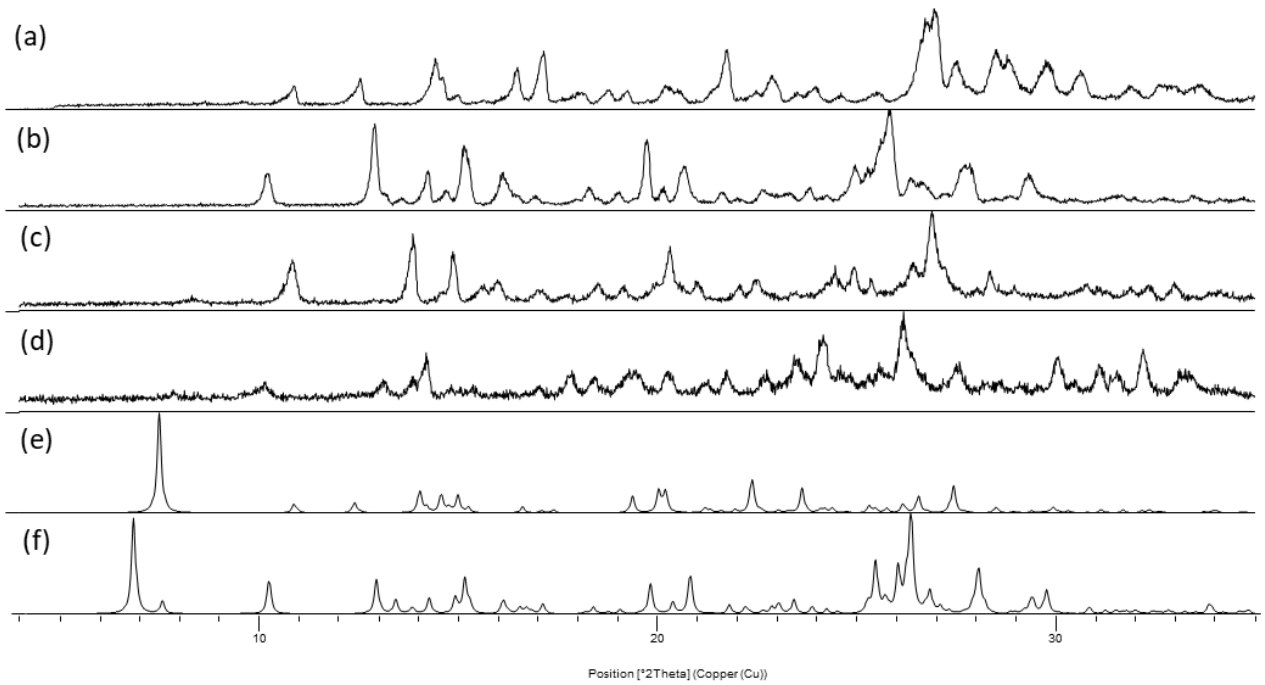

Figure S9. (a) XRPD pattern of the bulk sample of $(enro^+)_2(pim^{2-})\cdot 1.5H_2O$. (b) XRPD pattern of the bulk sample of $(enro^+)(pim^-)\cdot 3H_2O$. (c) XRPD pattern of the residue recovered after the solubility measurement of $(enro^+)_2(pim^{2-})\cdot 1.5H_2O$. (d) XRPD pattern of the residue recovered after the solubility measurement of $(enro^+)(pim^-)\cdot 3H_2O$. (e) Theoretical XRPD pattern calculated from the single crystal data of $(enro^+)_2(pim^{2-})\cdot 1.5H_2O$. (f) Theoretical XRPD pattern calculated from the single crystal data of $(enro^+)(pim^-)\cdot 3H_2O$

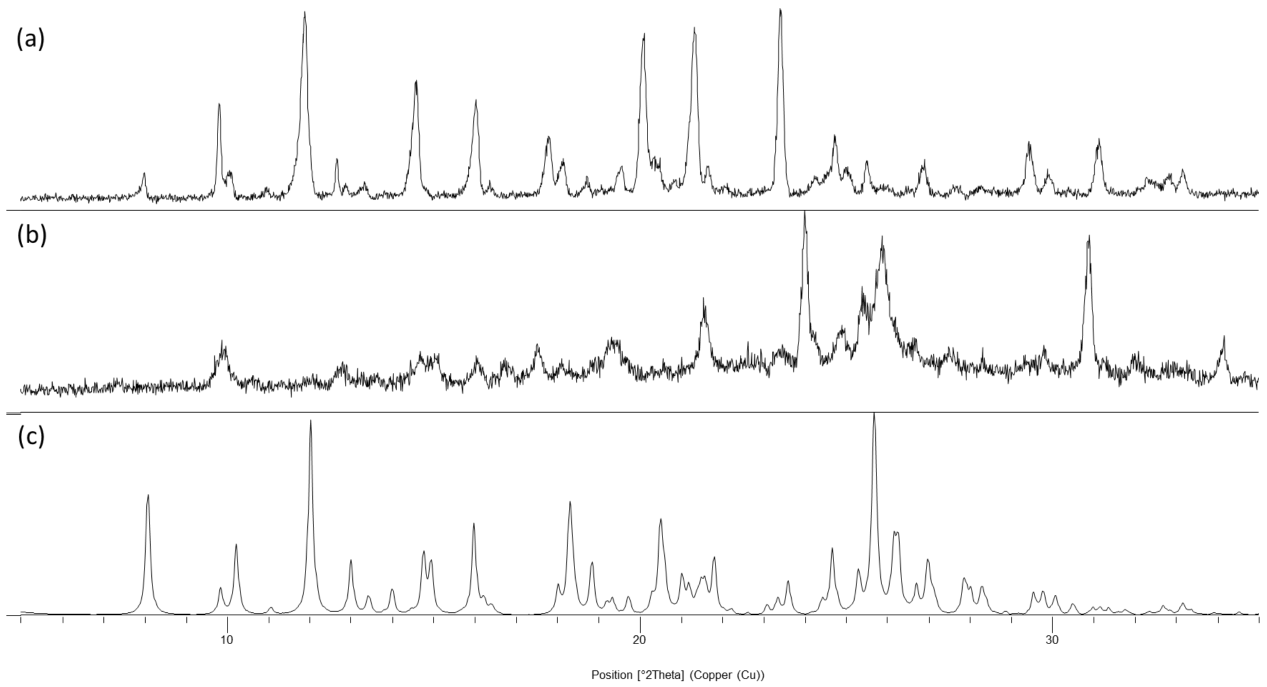

Figure S10. (a) XRPD pattern of the bulk sample of (enro$^+$)(sub$^-$)·H_2O. (b) XRPD pattern of the residue recovered after the solubility measurement. (c) Theoretical XRPD pattern calculated from the single crystal data.

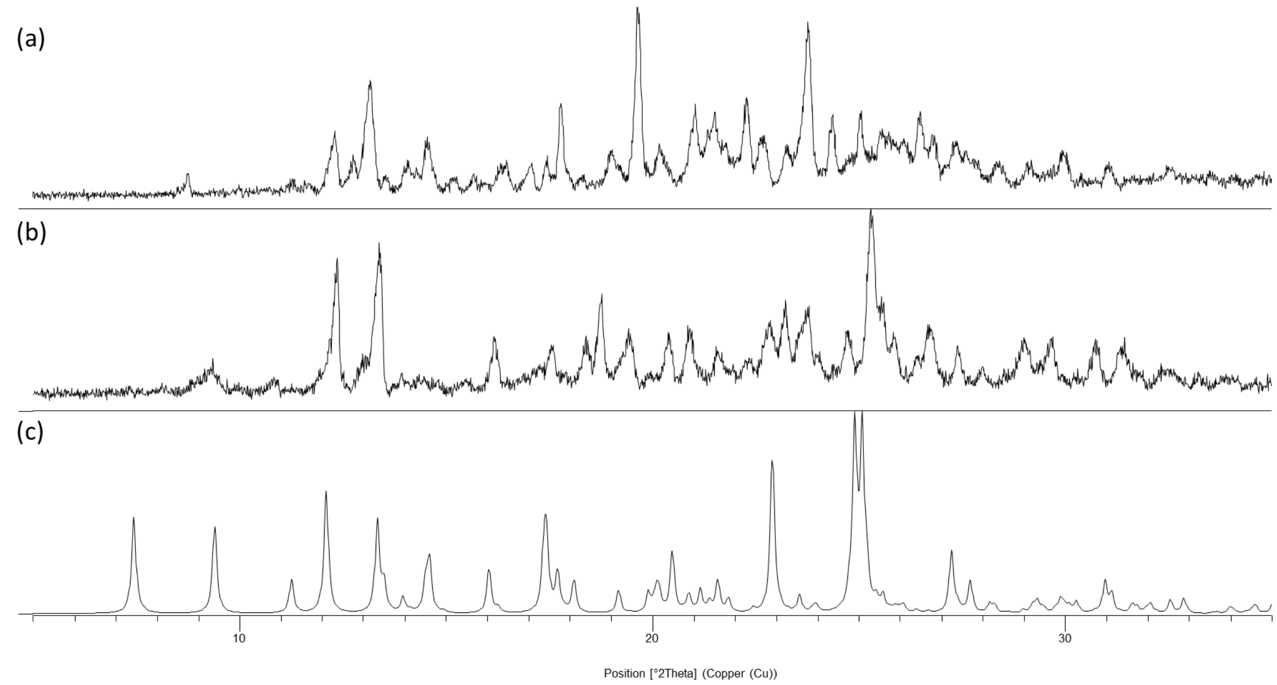

Figure S11. (a) XRPD pattern of the bulk sample of (enro$^+$)(az$^-$). (b) XRPD pattern of the residue recovered after the solubility measurement. (c) Theoretical XRPD pattern calculated from the single crystal data.

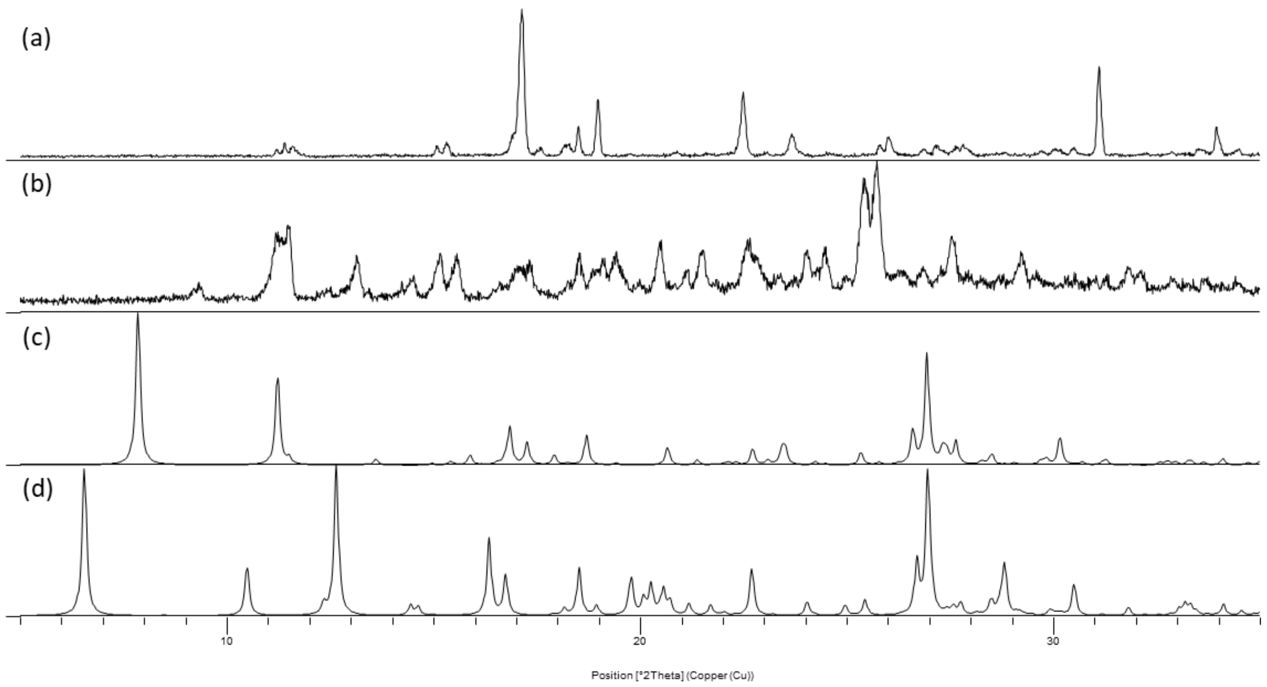

Figure S12. (a) XRPD pattern of the bulk sample of $(nor^{+})_2(adi^{2-})·2H_2O$ form I. (b) XRPD pattern of the residue recovered after the solubility measurement. (c) Theoretical XRPD pattern calculated from the single crystal data of $(nor^{+})_2(adi^{2-})·2H_2O$ form I.(d) Theoretical XRPD pattern calculated from the single crystal data of $(nor^{+})_2(adi^{2-})·2H_2O$ form II.

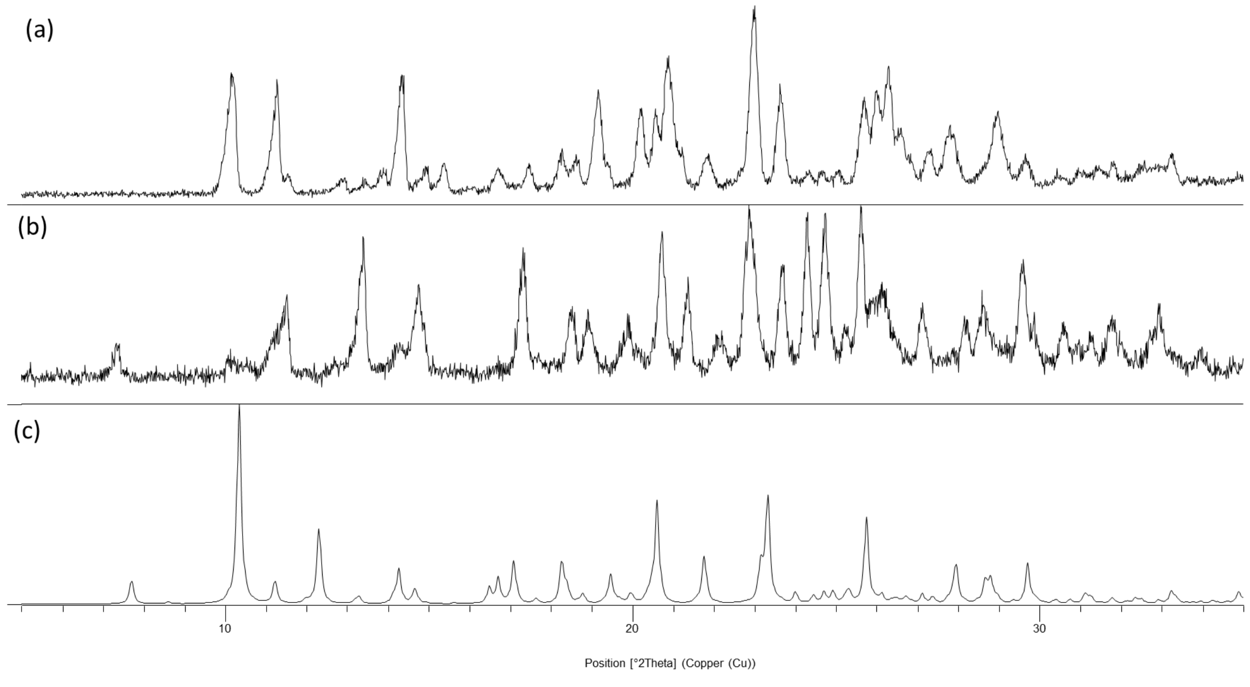

Figure S13. (a) XRPD pattern of the bulk sample of $(nor^{+})(pim^{-})$. (b) XRPD pattern of the residue after the solubility testing. (c) Theoretical XRPD pattern calculated from the single crystal data.

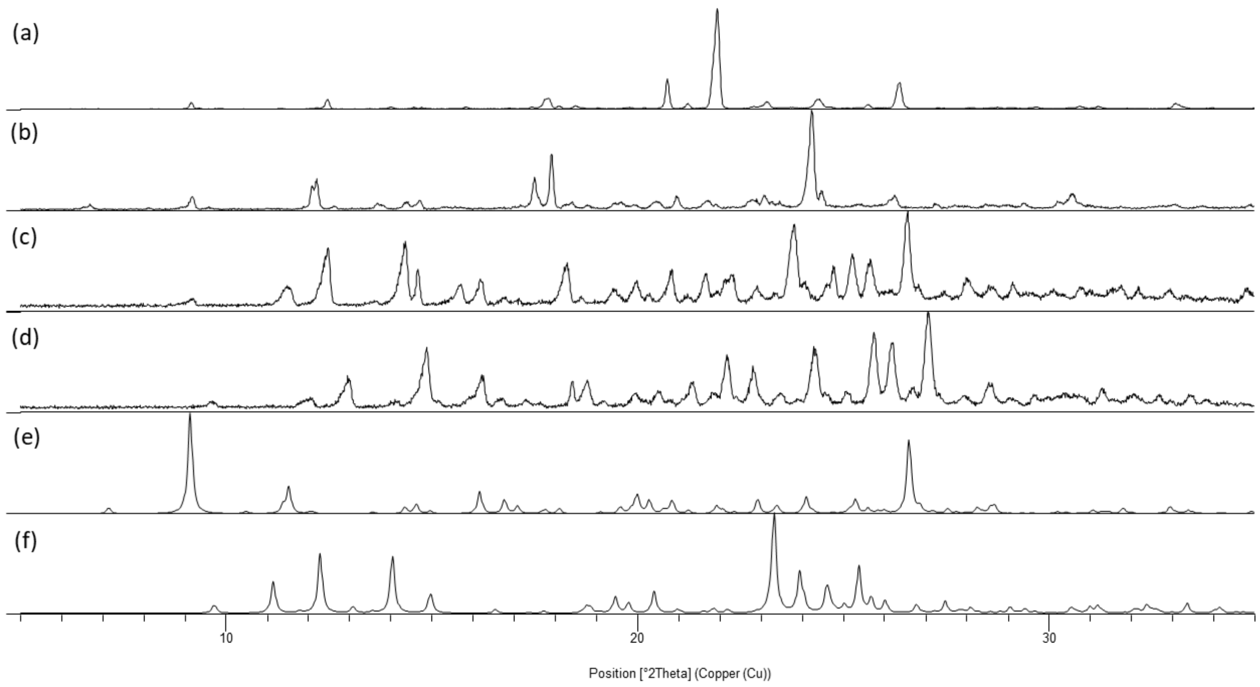

Figure S14. (a) XRPD pattern of the bulk sample of (nor^+)(sub^-)·$3H_2O$. (b) XRPD pattern of the bulk sample of (nor^+)(sub^-). (c) XRPD pattern of the residue recovered after the solubility measurement of (nor^+)(sub^-)·$3H_2O$. (d) XRPD pattern of the residue recovered after the solubility measurement of (nor^+)(sub^-). (e) Theoretical XRPD pattern calculated from the single crystal data of (nor^+)(sub^-)·$3H_2O$. (f) Theoretical XRPD pattern calculated from the single crystal data of (nor^+)(sub^-).

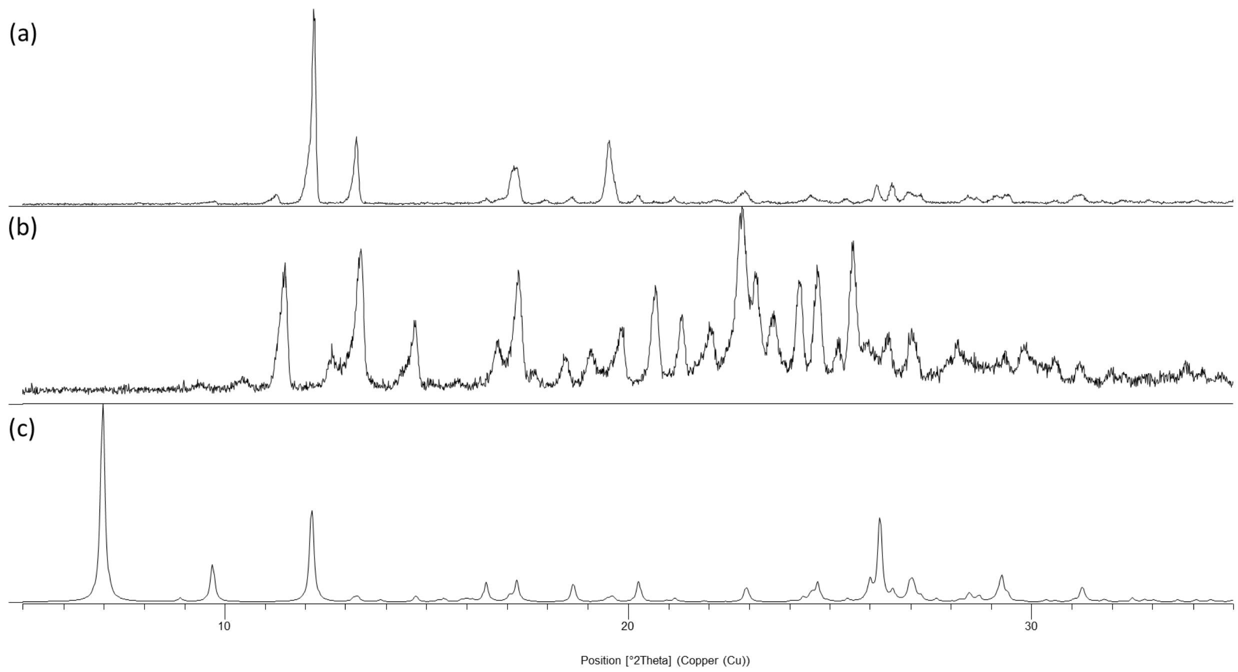

Figure S15. (a) XRPD pattern of the bulk sample of $(nor^+)_2(seb^{2-})\cdot 3H_2O$. (b) XRPD pattern of the residue recovered after the solubility measurement. (c) Theoretical XRPD pattern calculated from the single crystal data.

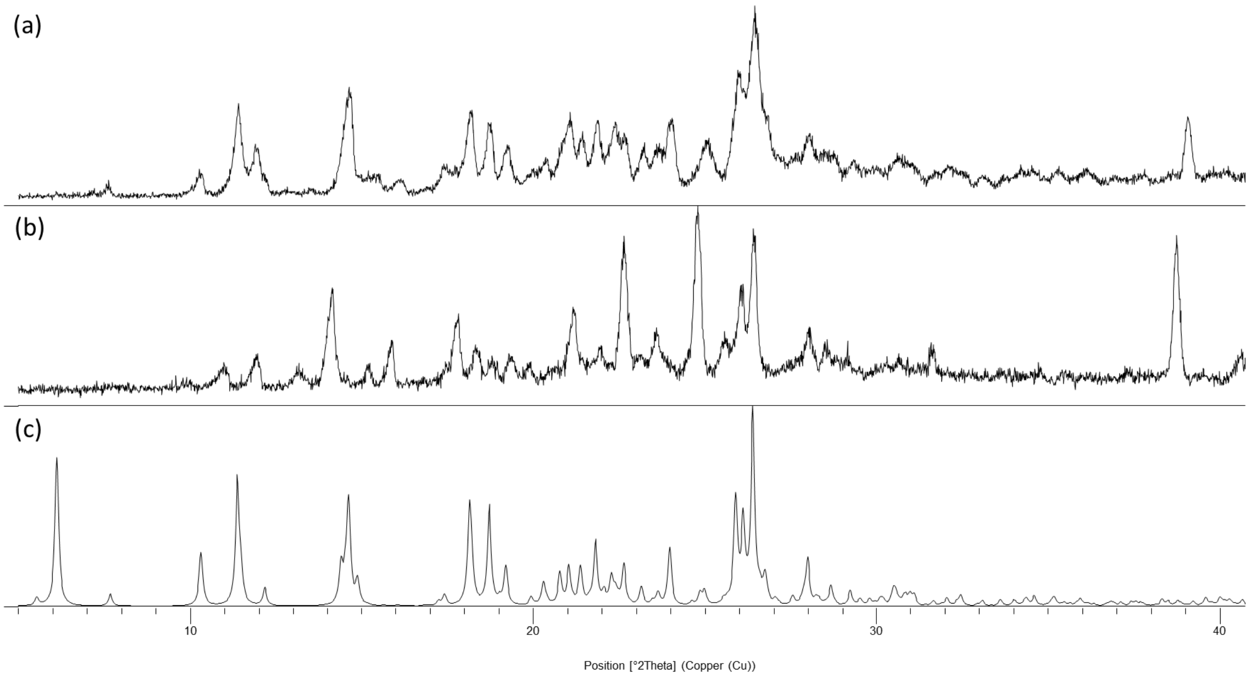

Figure S16. (a) XRPD pattern of the bulk sample of $(cip^+)(glu^-)$. (b) XRPD pattern of the residue recovered after the solubility measurement. (c) Theoretical XRPD pattern calculated from the single crystal data.

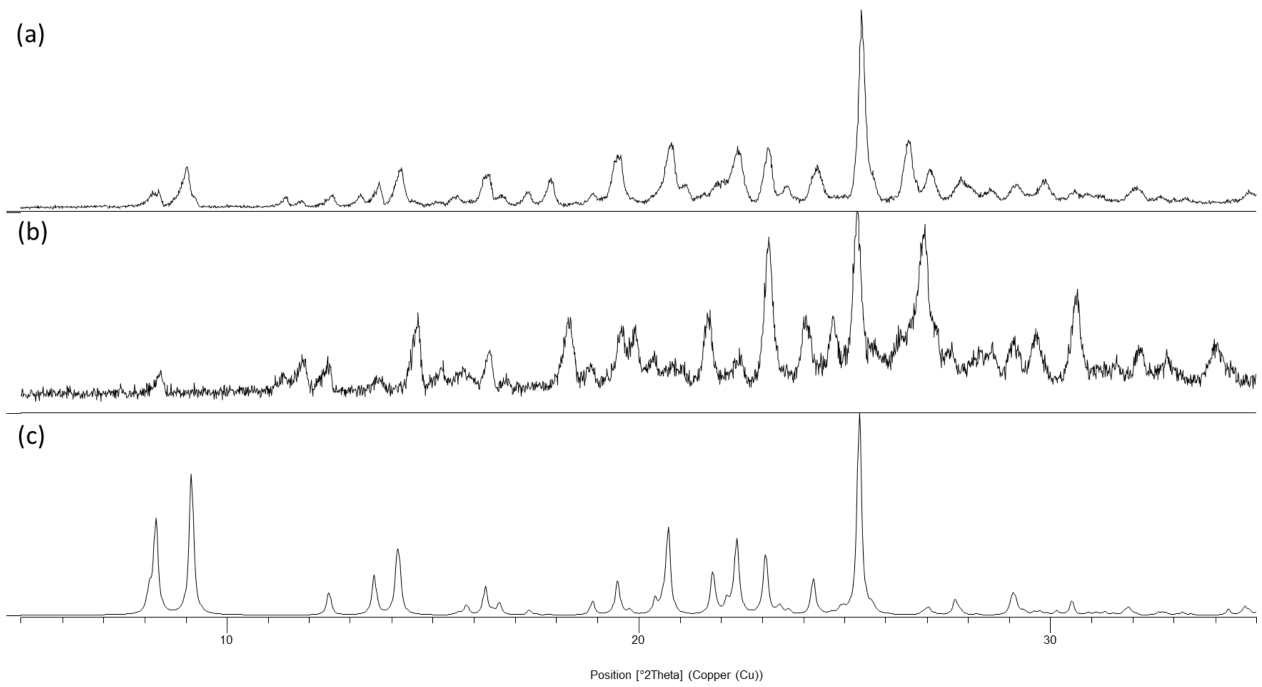

Figure S17. (a) XRPD pattern of the bulk sample of (cip$^+$)(pim$^-$). (b) XRPD pattern of the residue recovered after the solubility measurement. (c) Theoretical XRPD pattern calculated from the single crystal data.

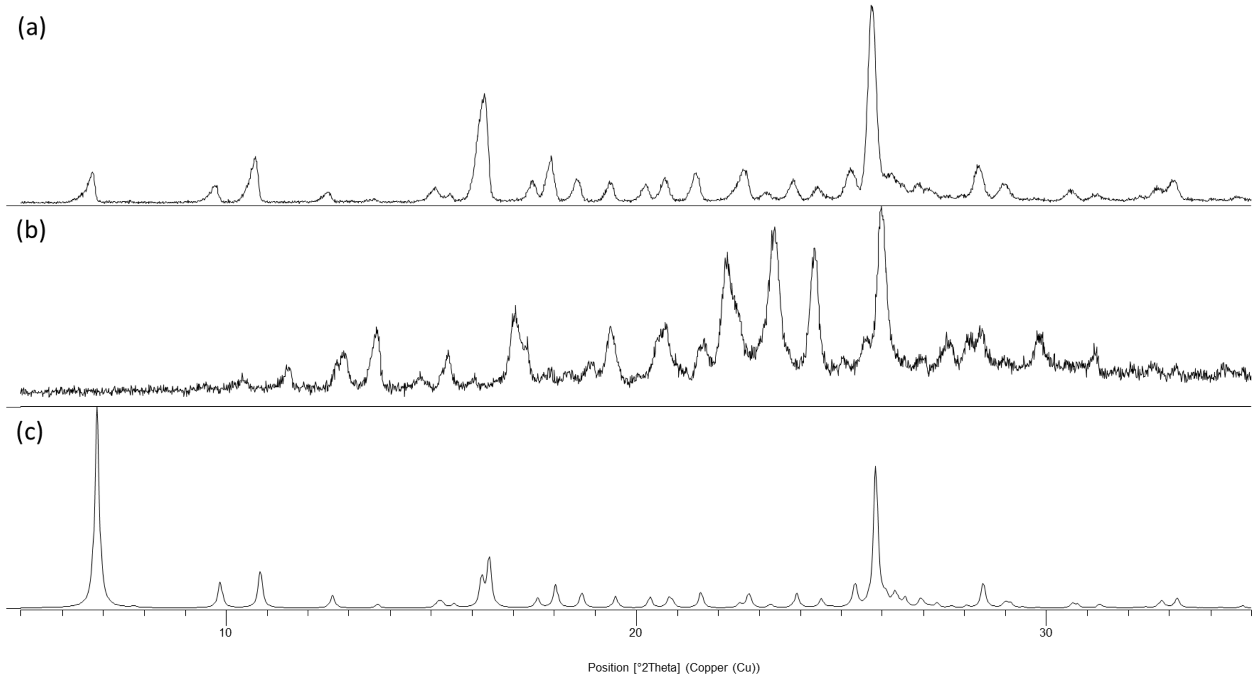

Figure S18. (a) XRPD pattern of the bulk sample of (cip$^+$)$_2$(sub^{2-})·4H_2O. (b) XRPD pattern of the residue recovered after the solubility measurement. (c) Theoretical XRPD pattern calculated from the single crystal data.

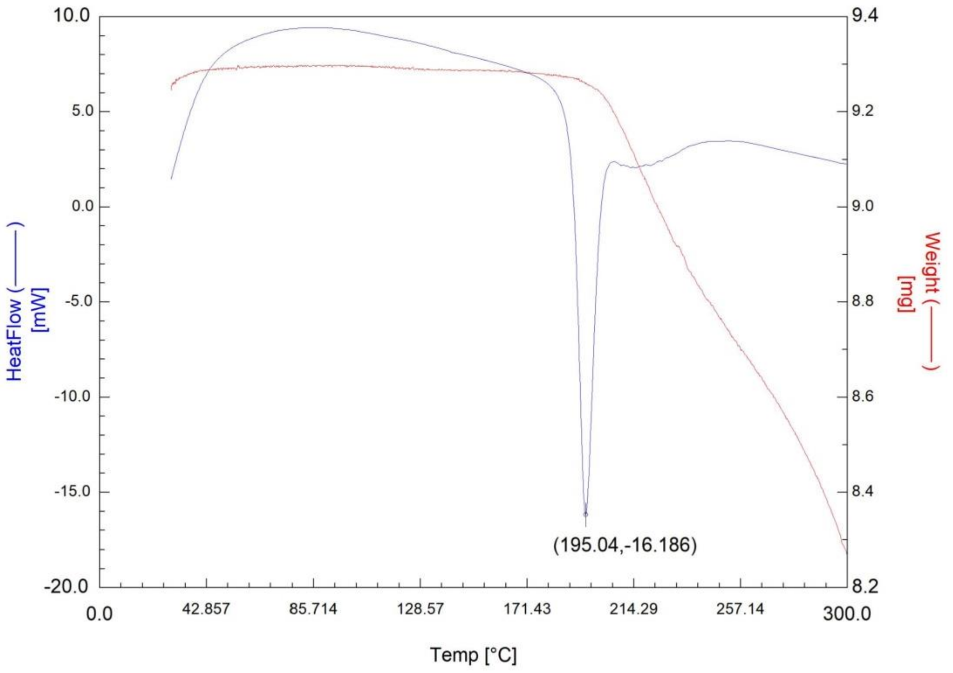

Figure S19. DSC/TGA plot of (cip$^+$)(pim$^-$).

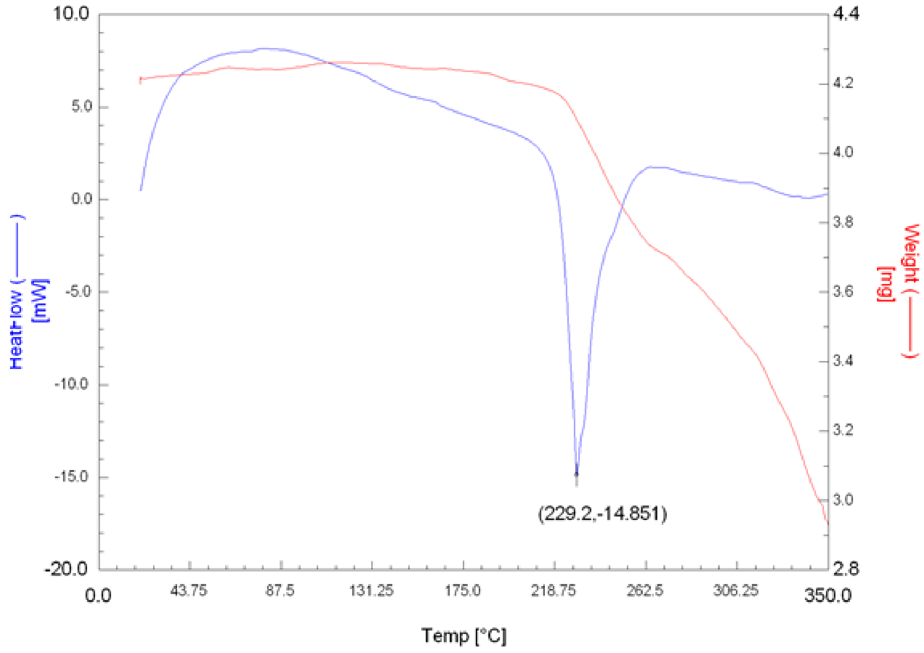

Figure S20. DSC/TGA plot of (cip$^+$)(glu$^-$).

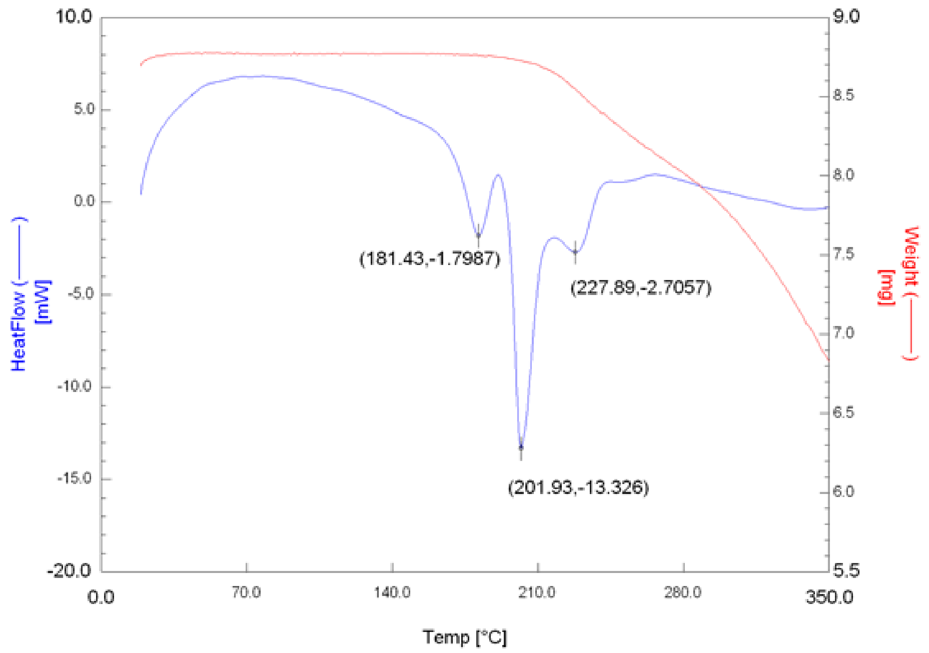

Figure S21. DSC/TGA plot of $(nor^{+})(pim^{-})$.

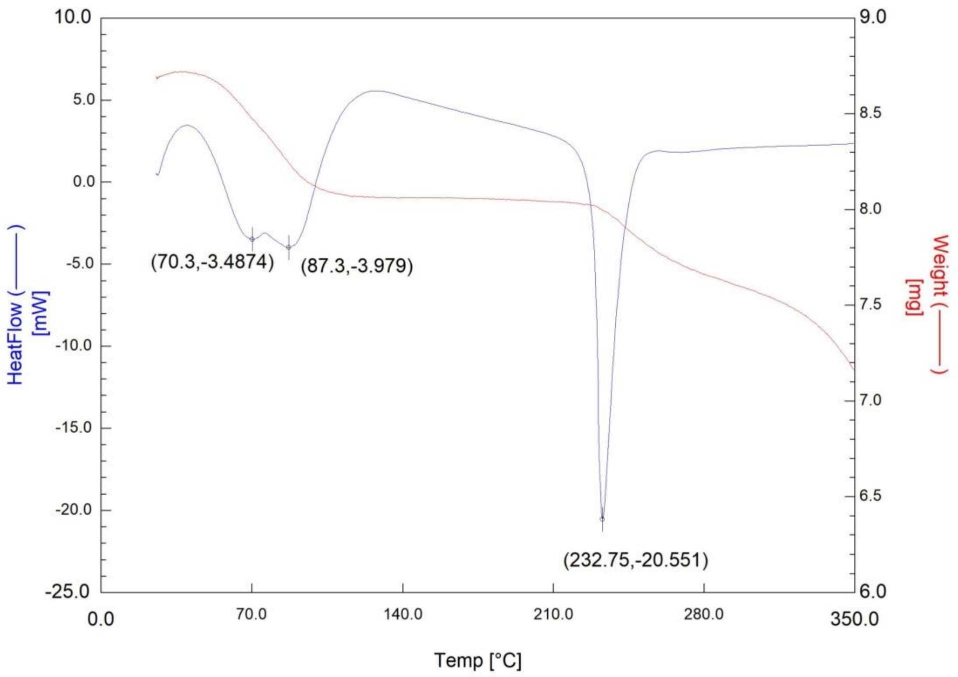

Figure S22. DSC/TGA plot of $(cip^{+})_2(sub^{2-})\cdot 4H_2O$.

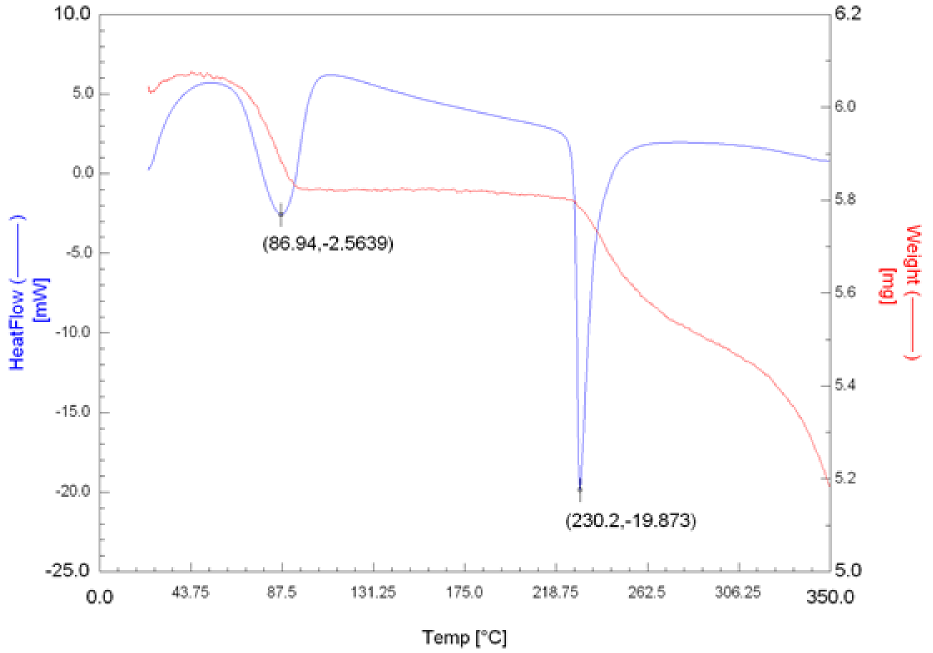

Figure S23. DSC/TGA plot of $(nor^{+})_2(adi^{2-})\cdot 2H_2O$ form I.

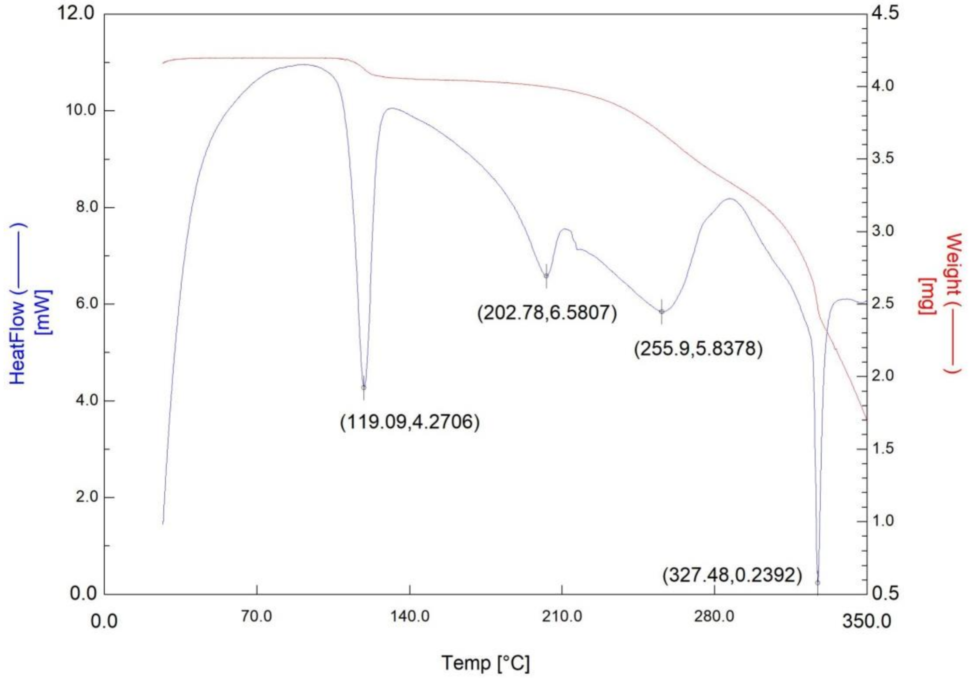

Figure S24. DSC/TGA plot of $(enro^{+})_2(pim^{2-})\cdot 1.5H_2O$.

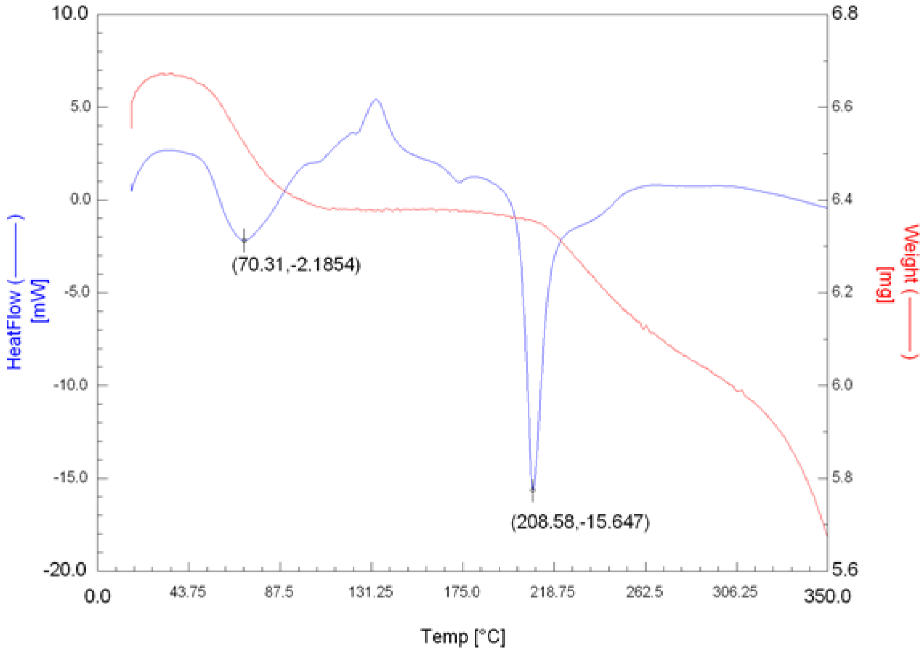

Figure S25. DSC/TGA plot of $(nor^{+})_2(seb^{2-})\cdot 3H_2O$.

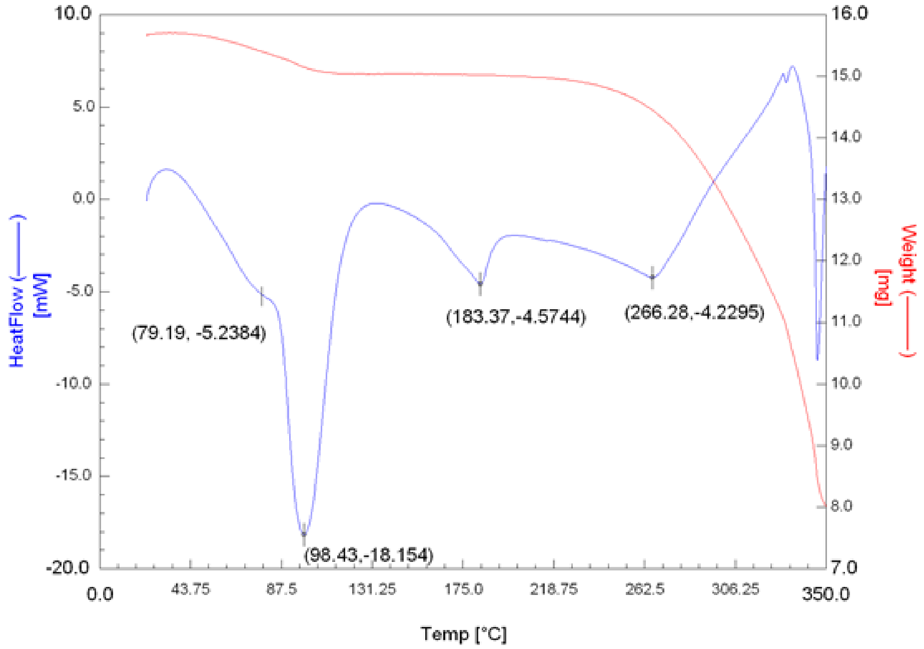

Figure S26. DSC/TGA plot of $(enro^{+})(sub^{-})\cdot H_2O$.

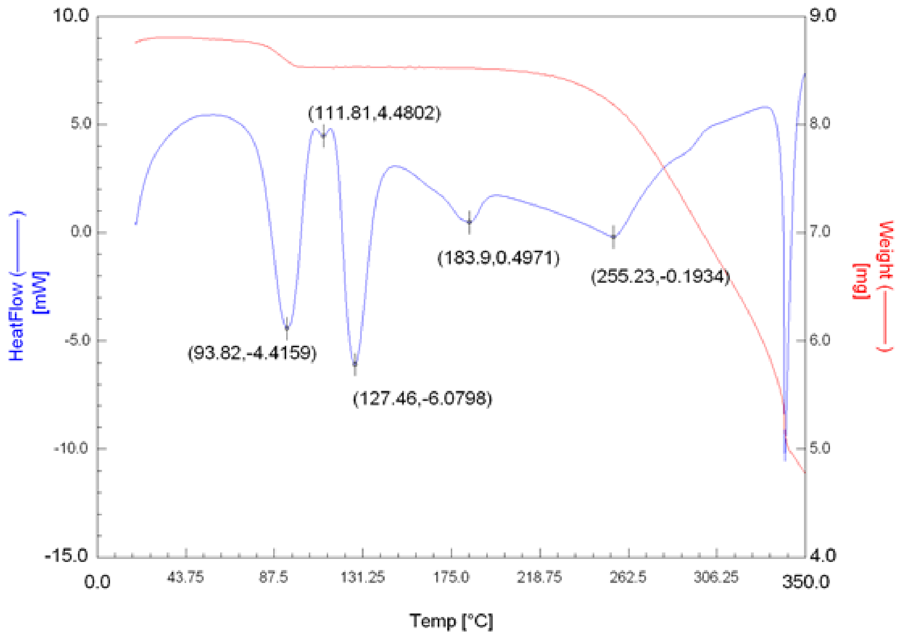

Figure S27. DSC/TGA plot of (enro$^+$)(pim$^-$)·$3H_2O$.

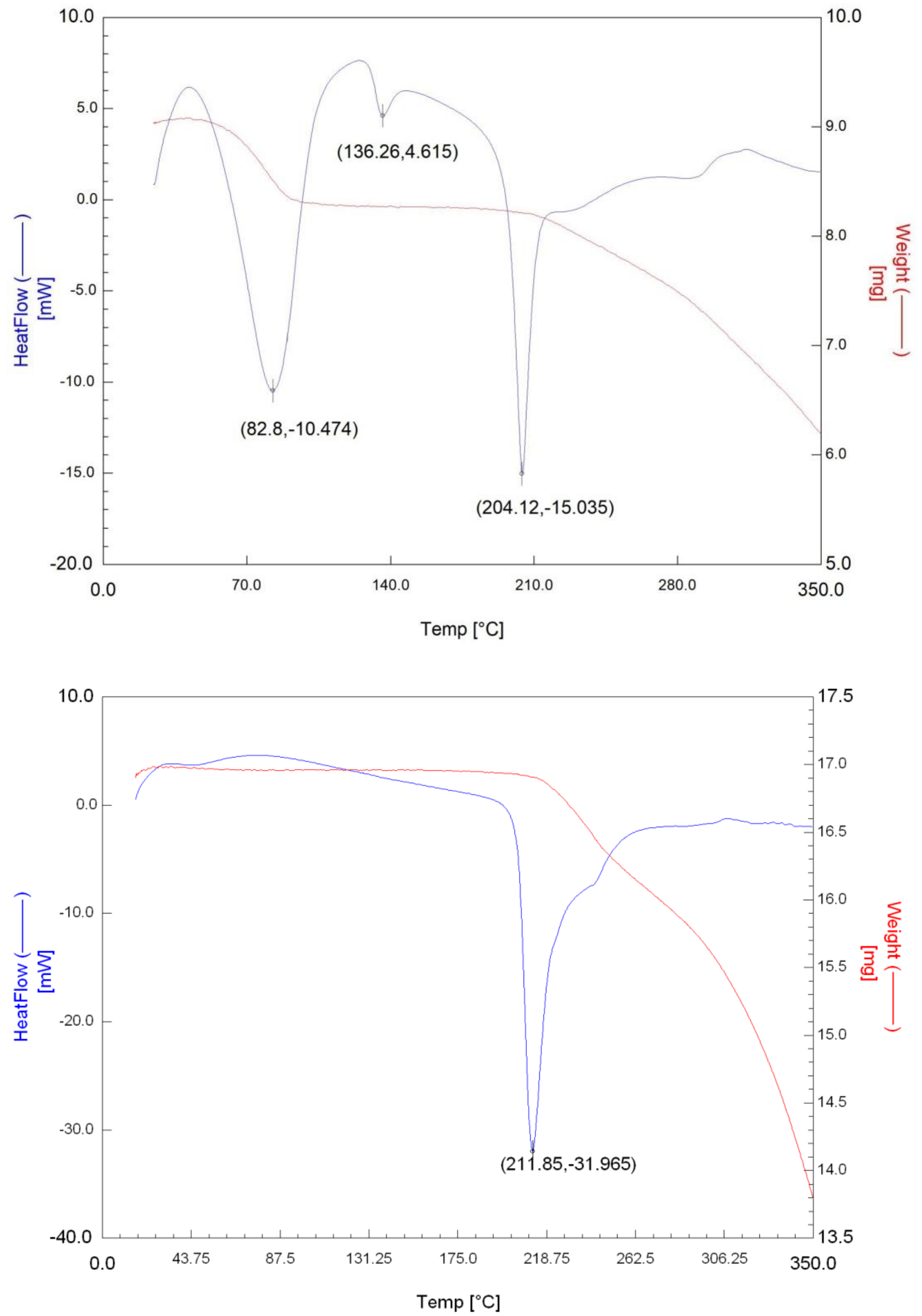

Figure S28. DSC/TGA plot of $(nor^{+})(sub^{-})\cdot 3H_2O$ before (top) and after (bottom) heating to 140 °C for 15 min.

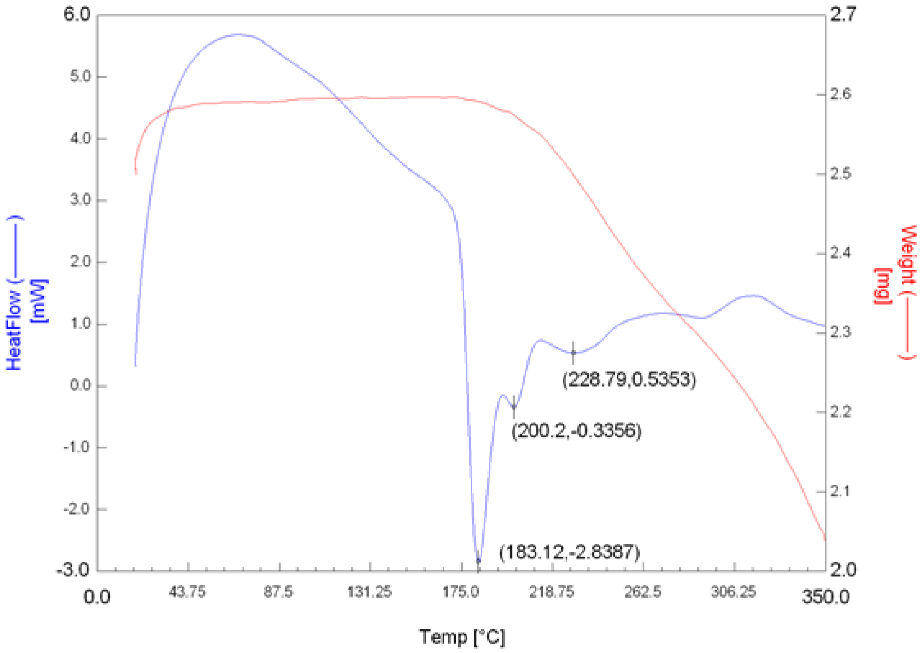

Figure S29. DSC/TGA plot of (nor^+)(sub^-).

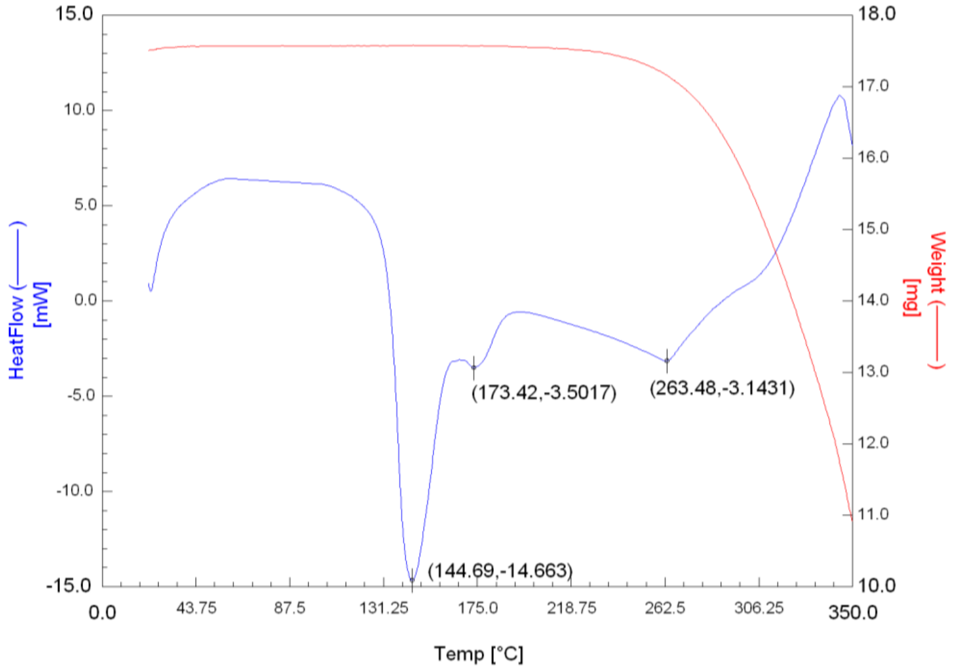

Figure S30. DSC/TGA plot of ($enro^+$)(az^-).

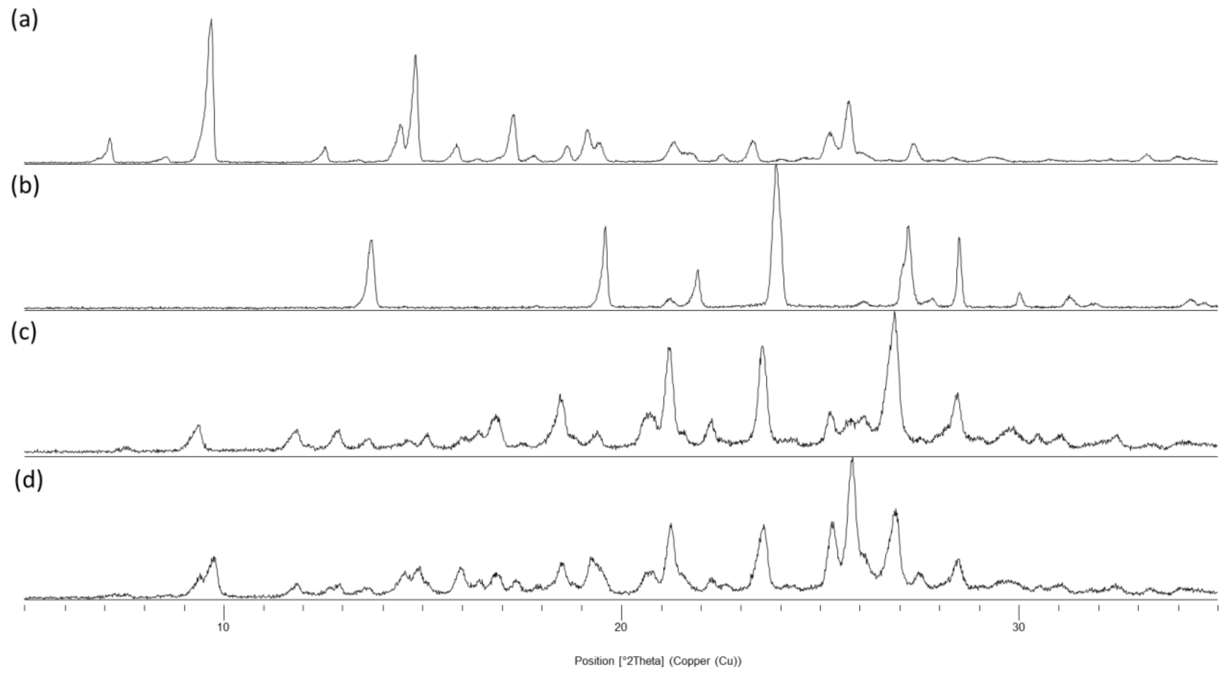

Figure S31. XRPD pattern of (a) enro, (b) glutaric acid, (c) a 1:1 mixture of enro and glutaric acid after ball-milling for 60 min at room temperature and (d) a 2:1 mixture of enro and glutaric acid after ball-milling for 60 min at room temperature.

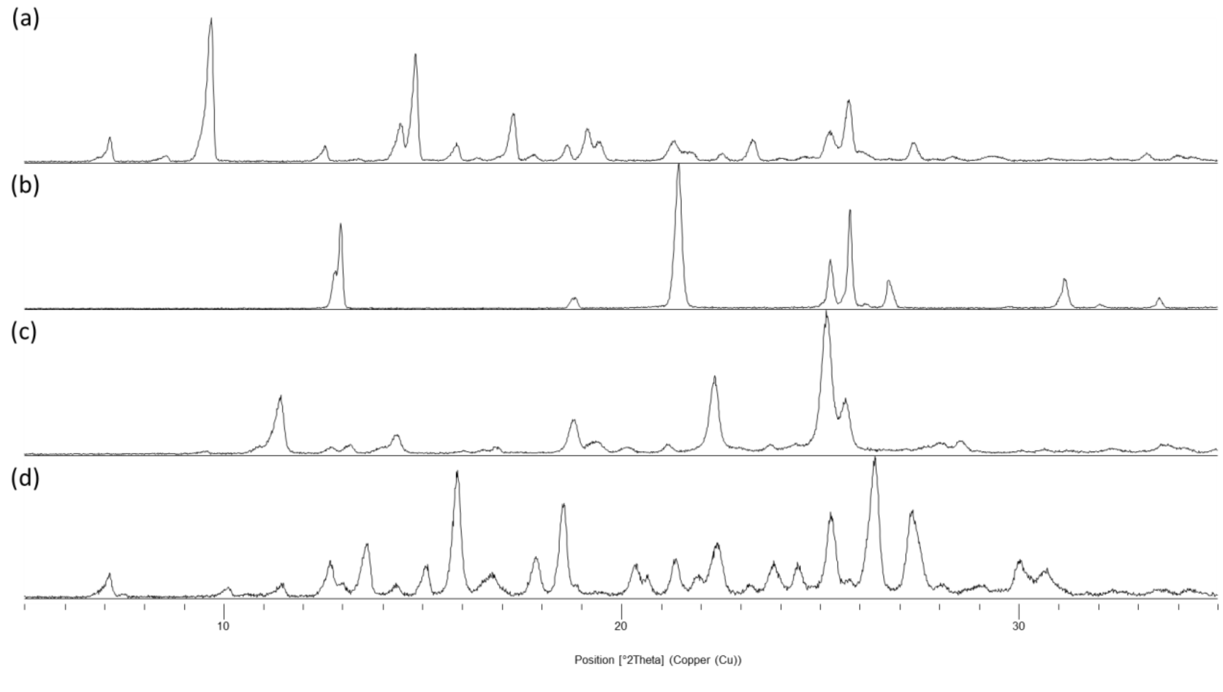

Figure S32. XRPD pattern of (a) enro, (b) adipic acid, (c) a 1:1 mixture of enro and adipic acid after ball-milling for 60 min at room temperature and (d) a 2:1 mixture of enro and adipic acid after ball-milling for 60 min at room temperature.

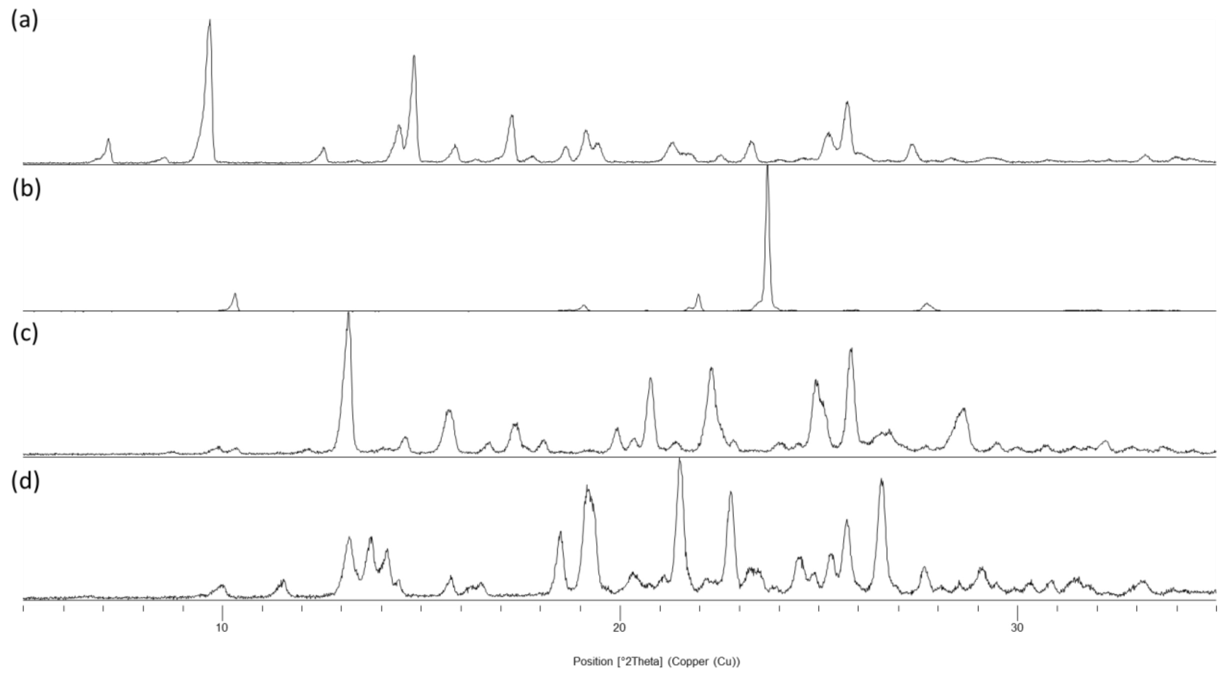

Figure S33. XRPD pattern of (a) enro, (b) pimelic acid, (c) a 1:1 mixture of enro and pimelic acid after ball-milling for 60 min at room temperature and (d) a 2:1 mixture of enro and pimelic acid after ball-milling for 60 min at room temperature.

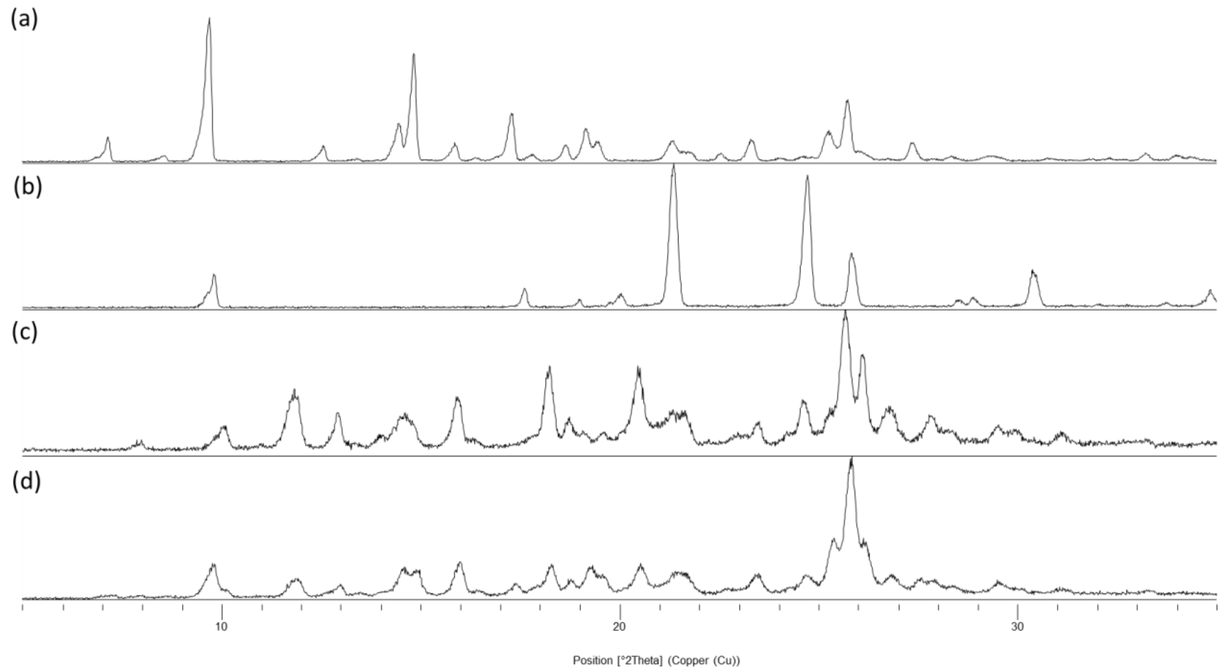

Figure S34. XRPD pattern of (a) enro, (b) suberic acid, (c) a 1:1 mixture of enro and suberic acid after ball-milling for 60 min at room temperature and (d) a 2:1 mixture of enro and suberic acid after ball-milling for 60 min at room temperature.

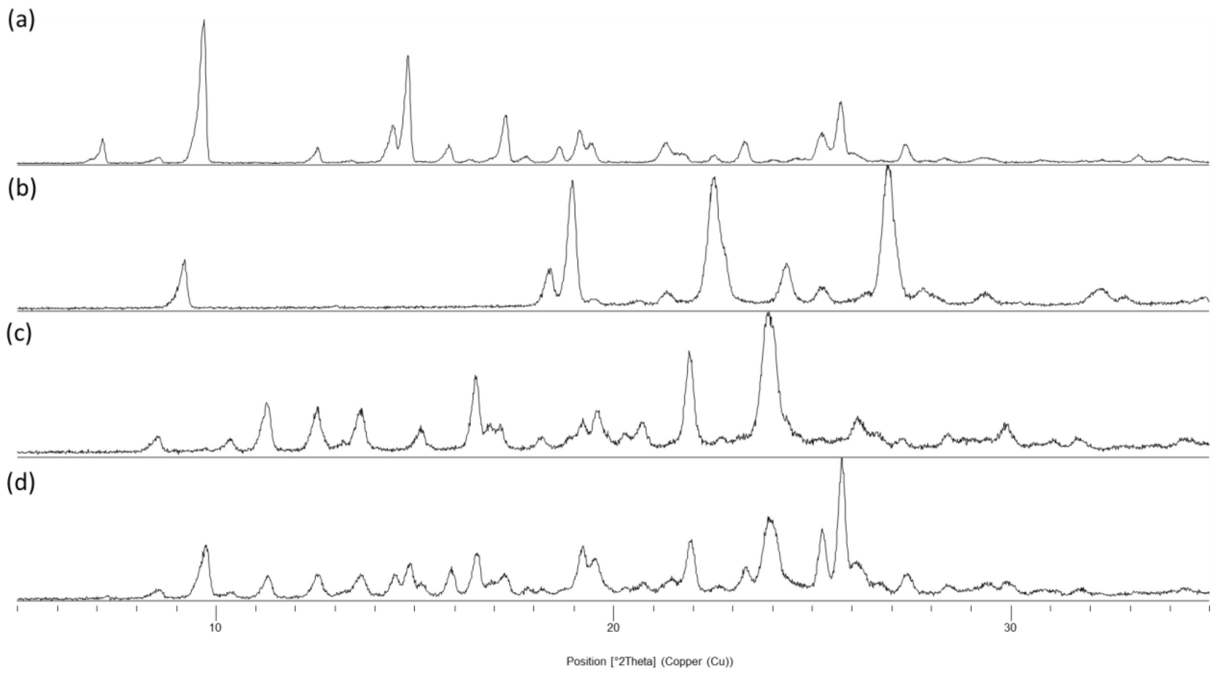

Figure S35. XRPD pattern of (a) enro, (b) azeliac acid, (c) a 1:1 mixture of enro and azeliac acid after ball-milling for 60 min at room temperature and (d) a 2:1 mixture of enro and azeliac acid after ball-milling for 60 min at room temperature.

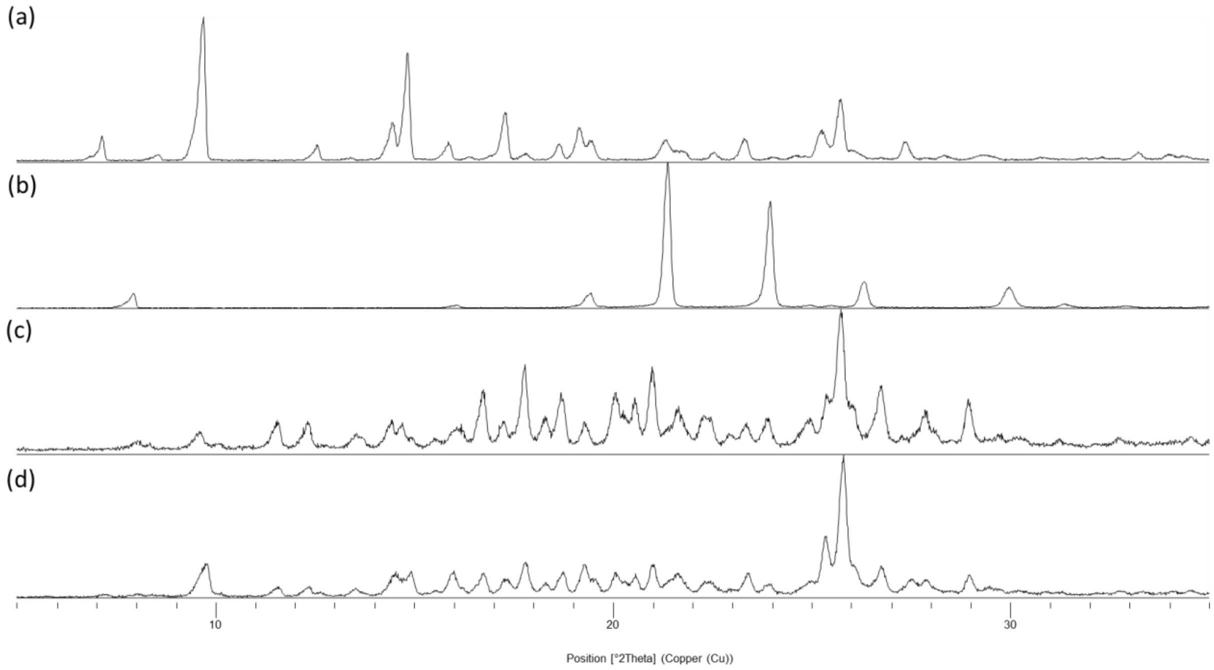

Figure S36. XRPD pattern of (a) enro, (b) sebacic acid and (c) a 1:1 mixture of enro and sebacic acid after ball-milling for 60 min at room temperature and (d) a 2:1 mixture of enro and sebacic acid after ball-milling for 60 min at room temperature.

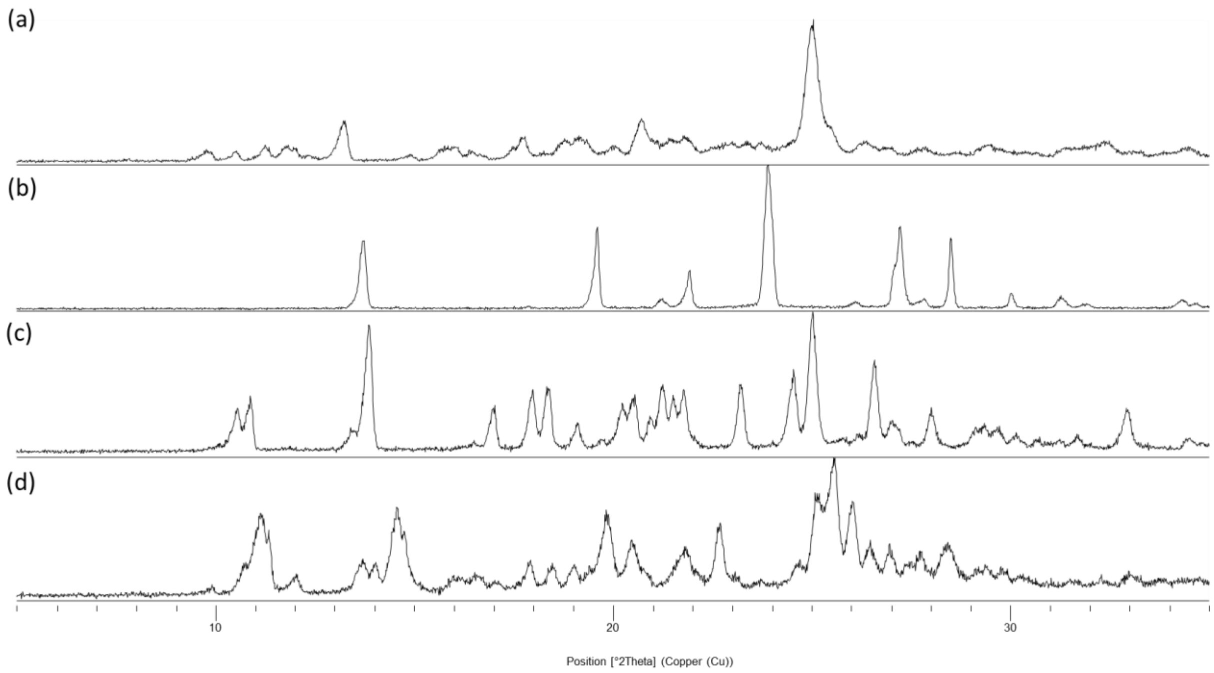

Figure S37. XRPD pattern of (a) nor, (b) glutaric acid and (c) a 1:1 mixture of nor and glutaric acid after ball-milling for 60 min at room temperature and (d) a 2:1 mixture of nor and glutaric acid after ball-milling for 60 min at room temperature.

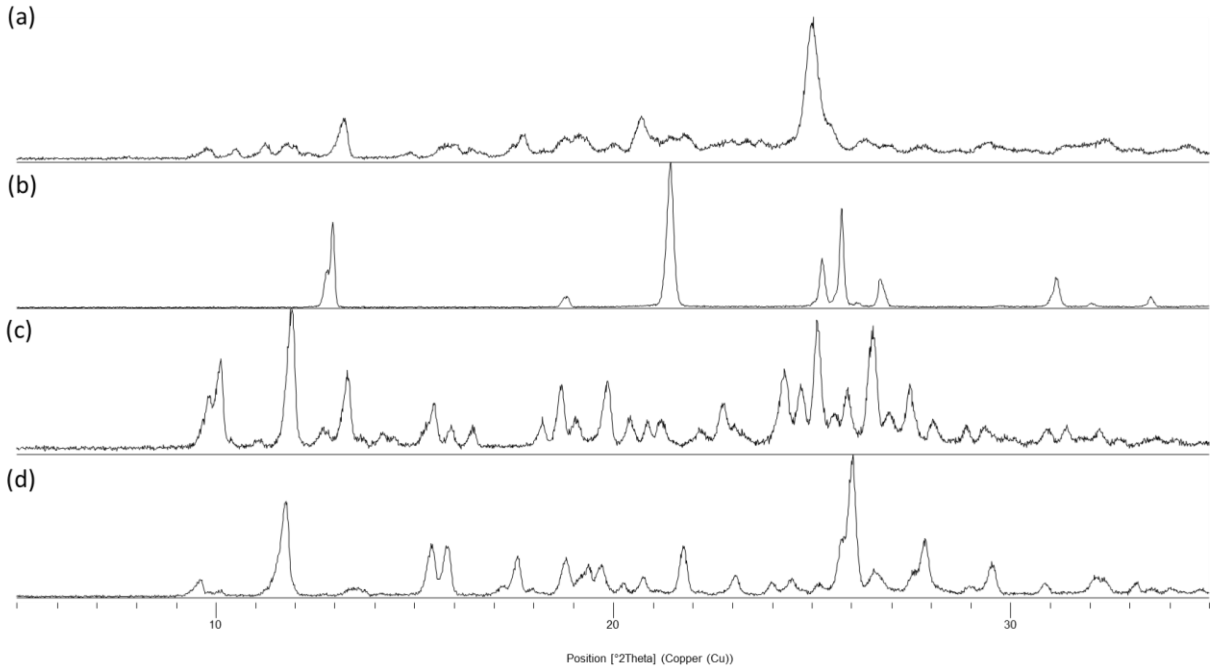

Figure S38. XRPD pattern of (a) nor, (b) adipic acid and (c) a 1:1 mixture of nor and adipic acid after ball-milling for 60 min at room temperature and (d) a 2:1 mixture of nor and adipic acid after ball-milling for 60 min at room temperature.

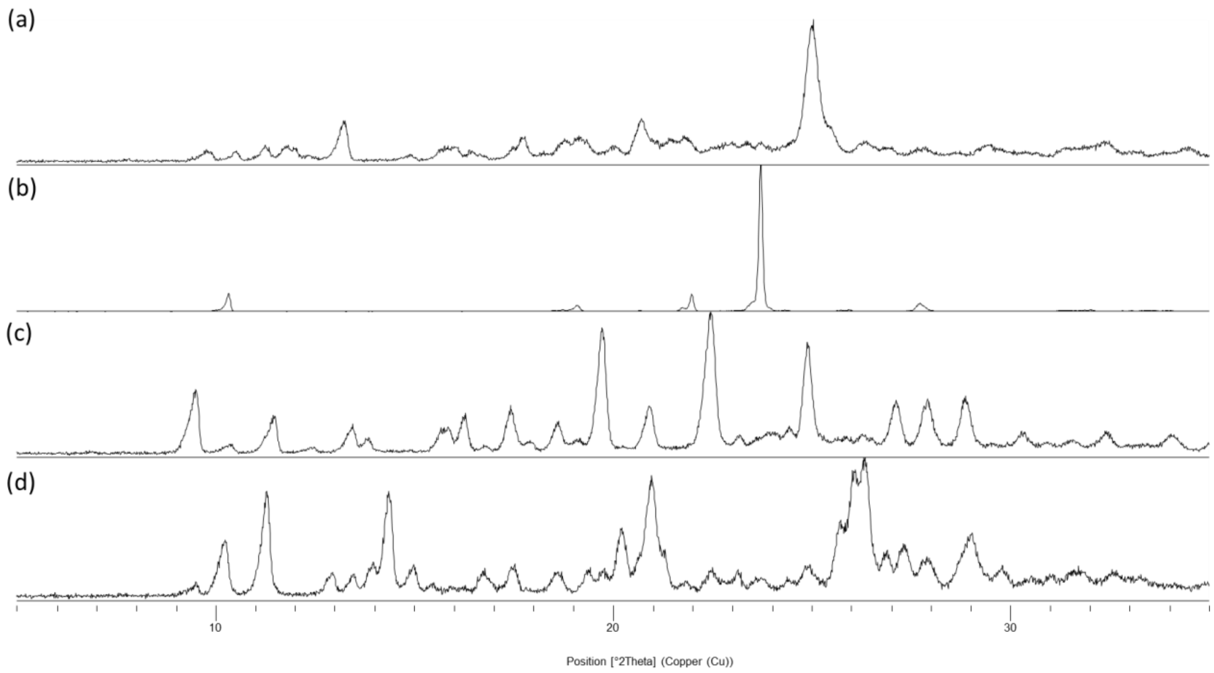

Figure S39. XRPD pattern of (a) nor, (b) pimelic acid and (c) a 1:1 mixture of nor and pimelic acid after ball-milling for 60 min at room temperature and (d) a 2:1 mixture of nor and pimelic acid after ball-milling for 60 min at room temperature.

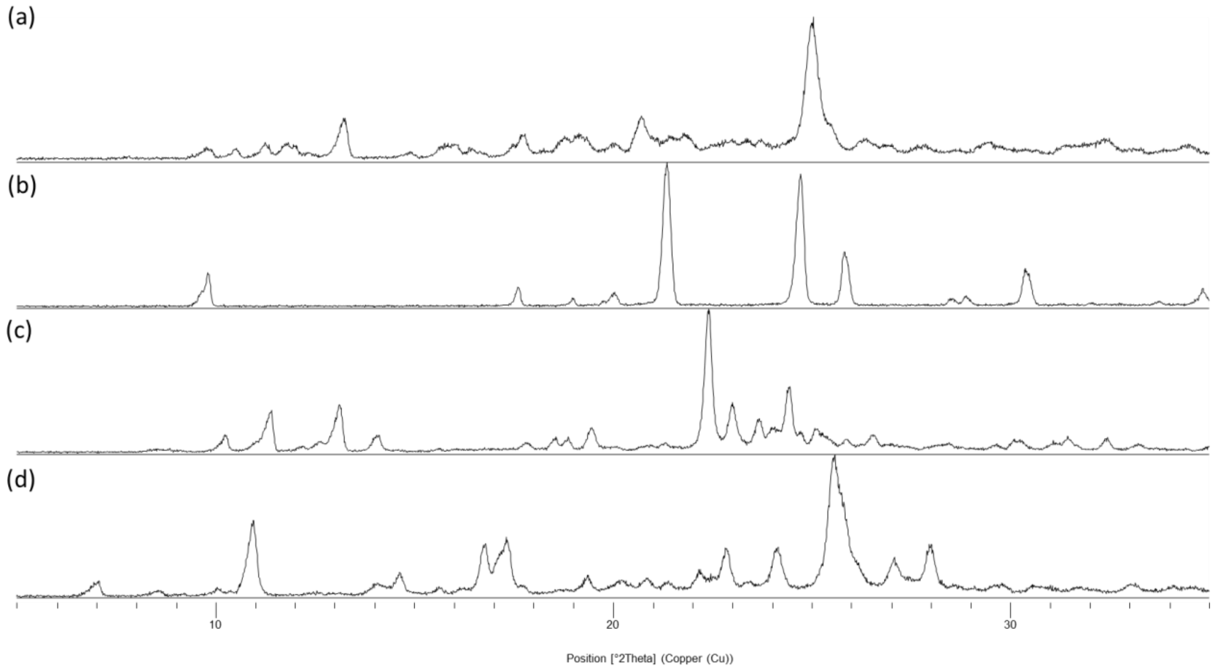

Figure S40. XRPD pattern of (a) nor, (b) suberic acid and (c) a 1:1 mixture of nor and suberic acid after ball-milling for 60 min at room temperature and (d) a 2:1 mixture of nor and suberic acid after ball-milling for 60 min at room temperature.

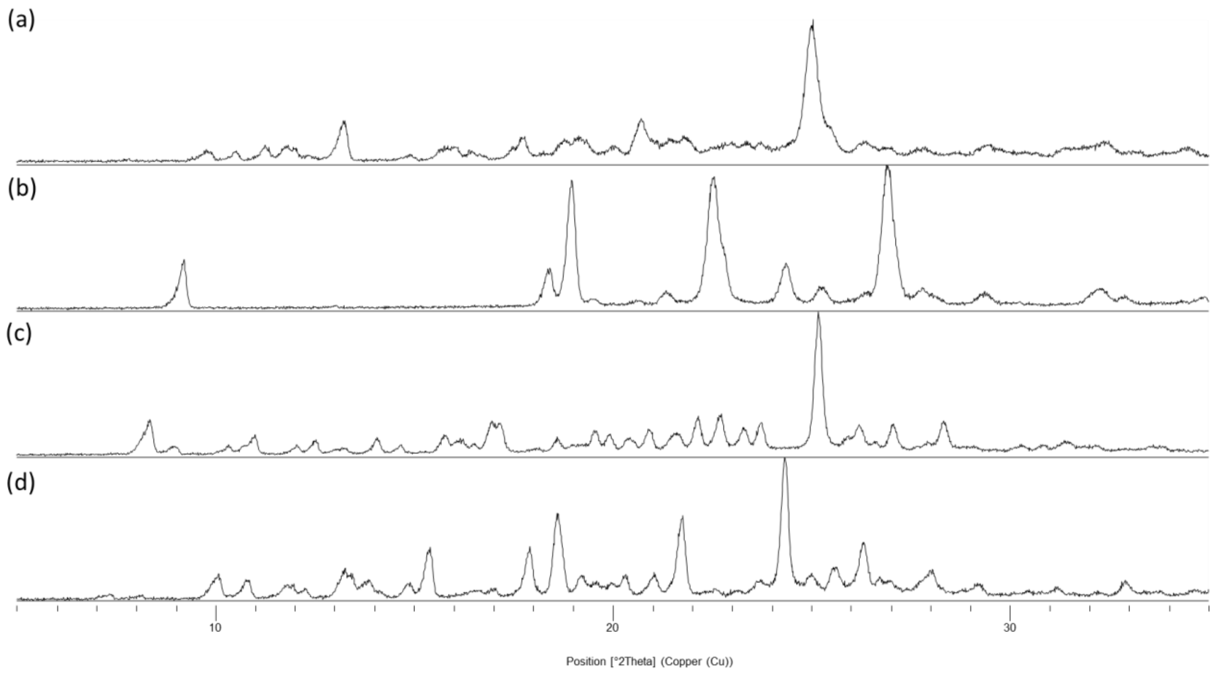

Figure S41. XRPD pattern of (a) nor, (b) azeliac acid, (c) a 1:1 mixture of nor and azeliac acid after ball-milling for 60 min at room temperature and (d) a 2:1 mixture of nor and azeliac acid after ball-milling for 60 min at room temperature.

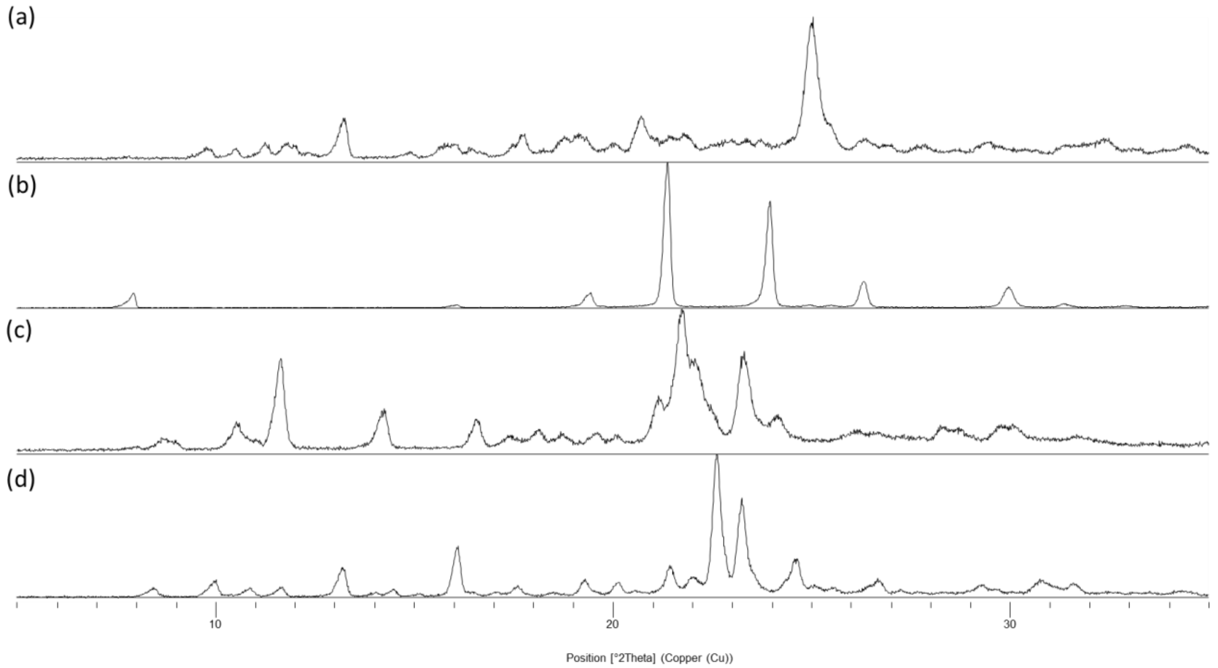

Figure S42. XRPD pattern of (a) nor, (b) sebacic acid, (c) a 1:1 mixture of nor and sebacic acid after ball-milling for 60 min at room temperature and (d) a 2:1 mixture of nor and sebacic acid after ball-milling for 60 min at room temperature.

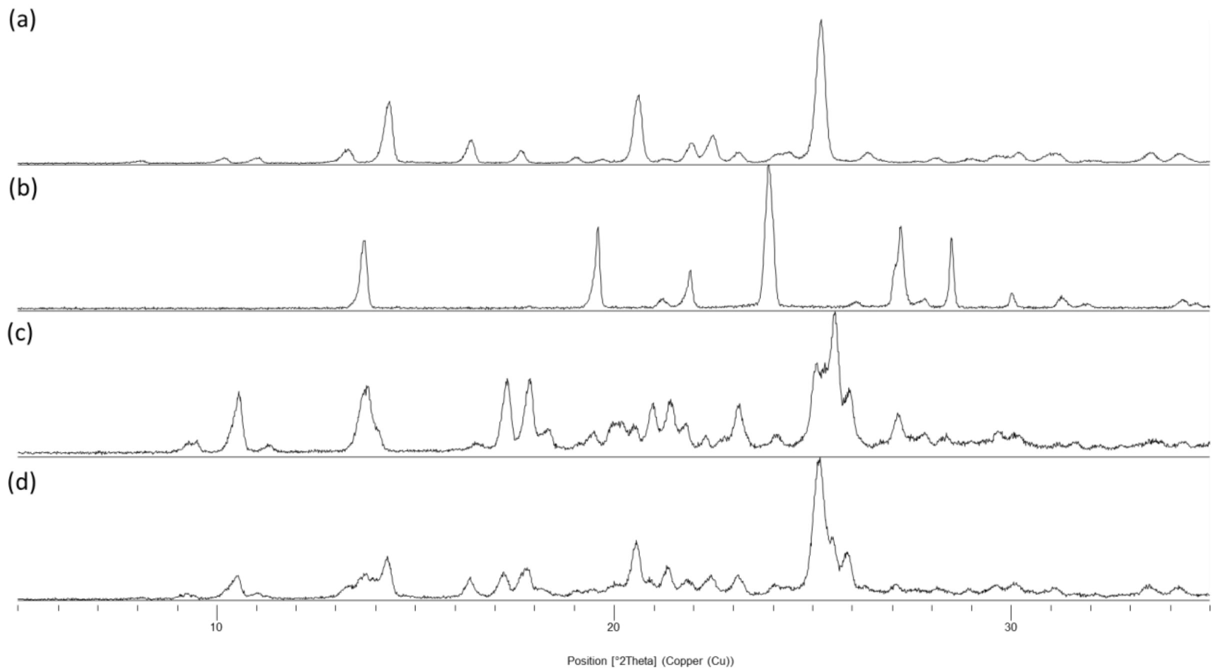

Figure S43. XRPD pattern of (a) cip, (b) glutaric acid, (c) a 1:1 mixture of cip and glutaric acid after ball-milling for 60 min at room temperature and (d) a 2:1 mixture of cip and glutaric acid after ball-milling for 60 min at room temperature.

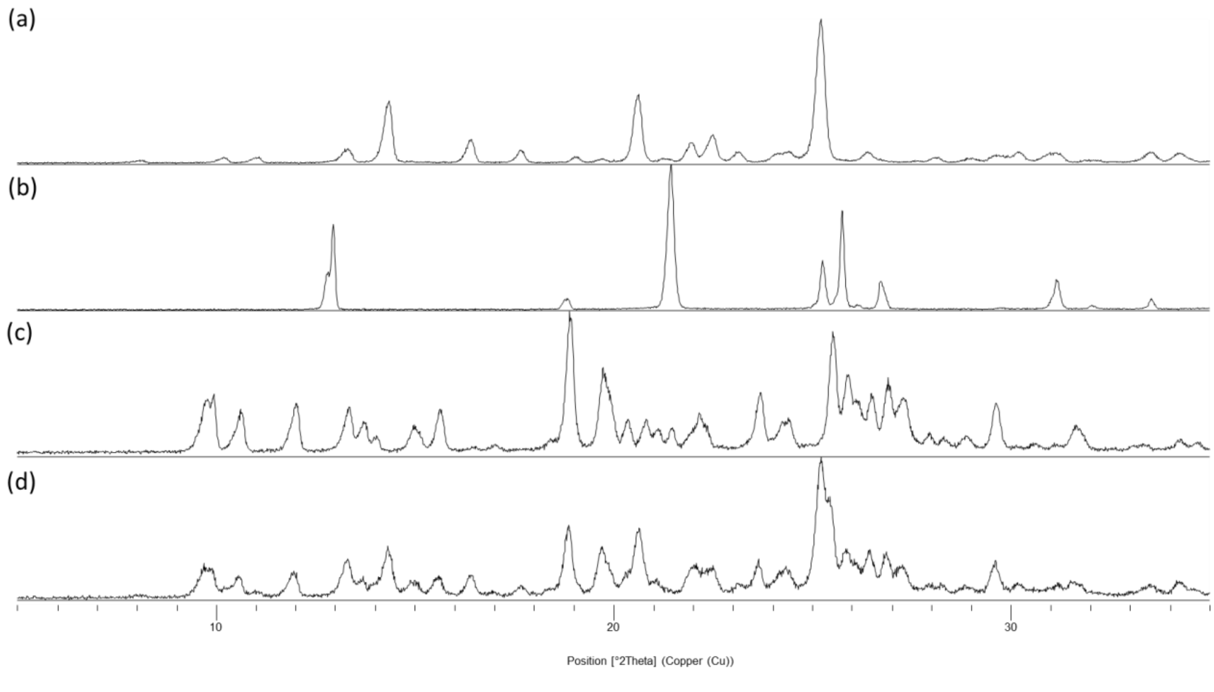

Figure S44. XRPD pattern of (a) cip, (b) adipic acid, (c) a 1:1 mixture of cip and adipic acid after ball-milling for 60 min at room temperature and (d) a 2:1 mixture of cip and adipic acid after ball-milling for 60 min at room temperature.

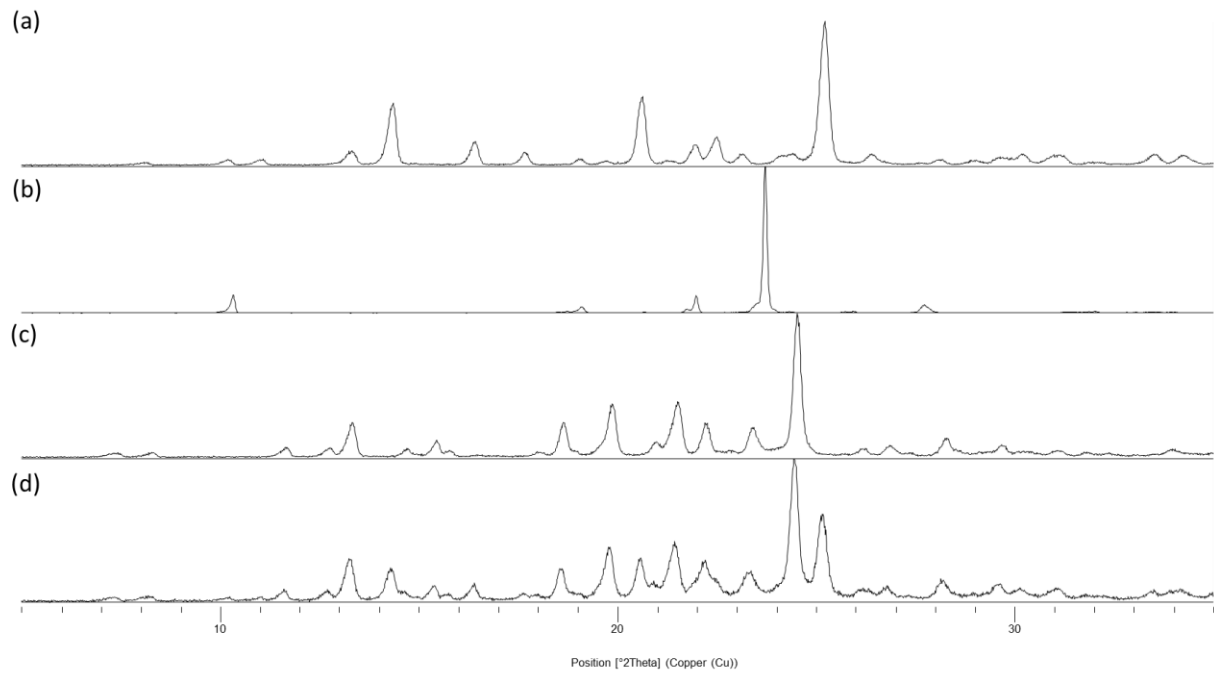

Figure S45. XRPD pattern of (a) cip, (b) pimelic acid, (c) a 1:1 mixture of cip and pimelic acid after ball-milling for 60 min at room temperature and (d) a 2:1 mixture of cip and pimelic acid after ball-milling for 60 min at room temperature.

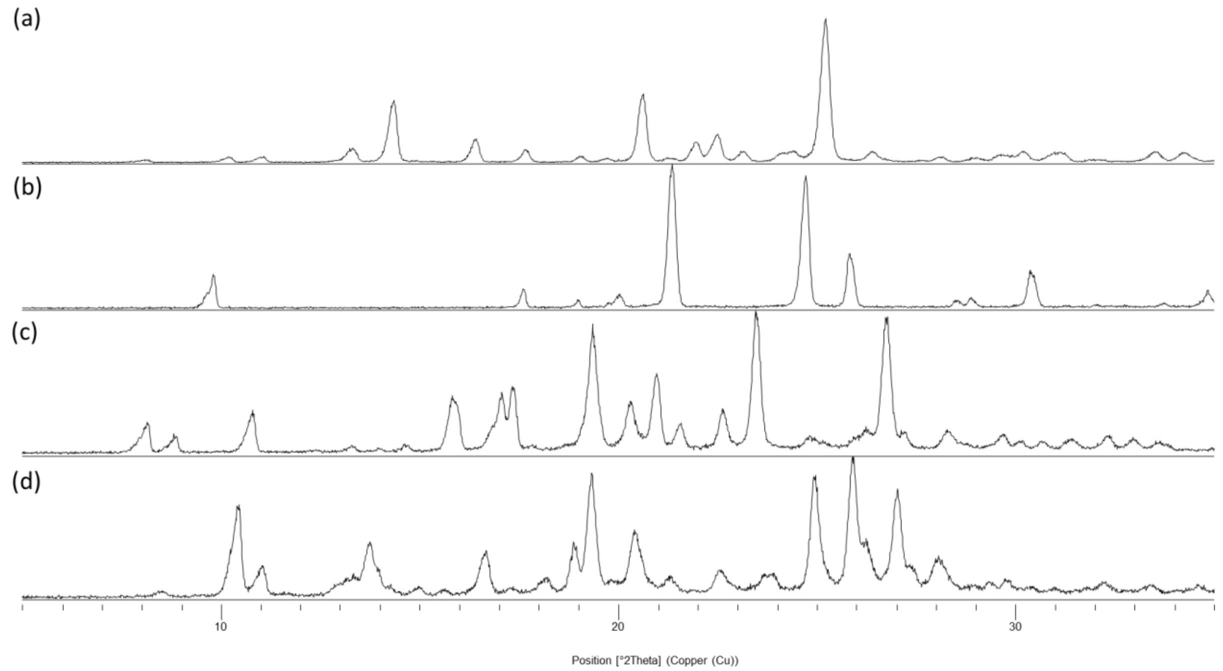

Figure S46. XRPD pattern of (a) cip, (b) suberic acid, (c) a 1:1 mixture of cip and suberic acid after ball-milling for 60 min at room temperature and (d) a 2:1 mixture of cip and suberic acid after ball-milling for 60 min at room temperature.

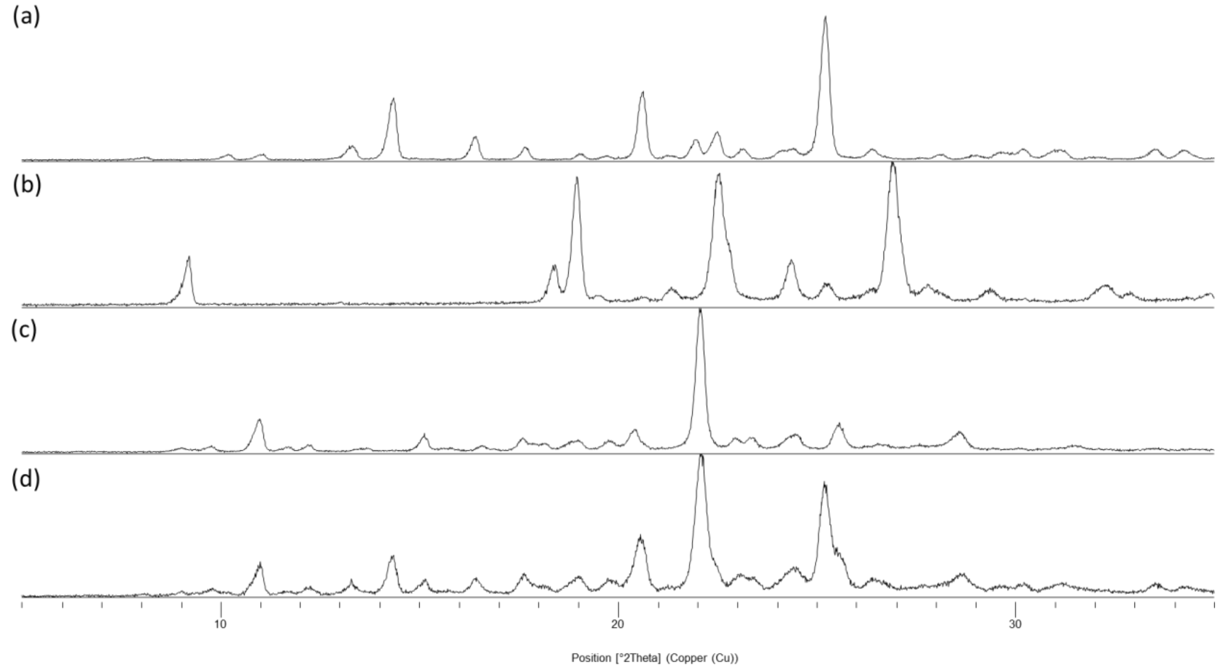

Figure S47. XRPD pattern of (a) cip, (b) azeliac acid, (c) a 1:1 mixture of cip and azeliac acid after ball-milling for 60 min at room temperature and (d) a 2:1 mixture of cip and azeliac acid after ball-milling for 60 min at room temperature.

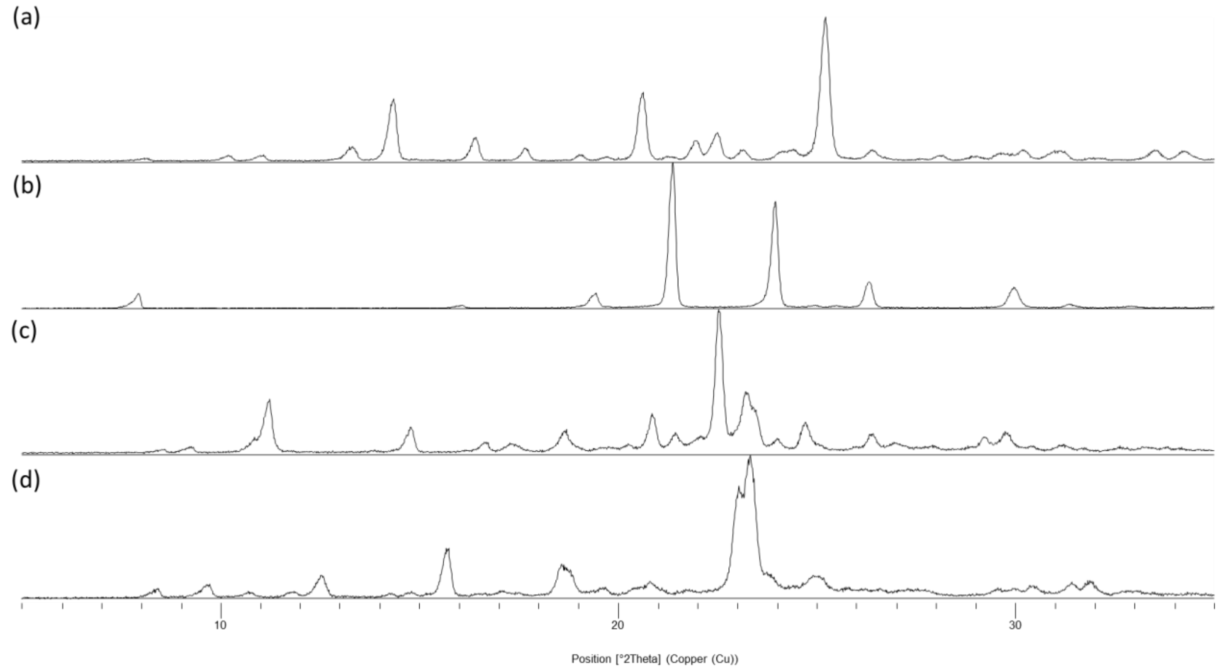

Figure S48. XRPD pattern of (a) cip, (b) sebacic acid, (c) a 1:1 mixture of cip and sebacic acid after ball-milling for 60 min at room temperature and (d) a 2:1 mixture of cip and sebacic acid after ball-milling for 60 min at room temperature.

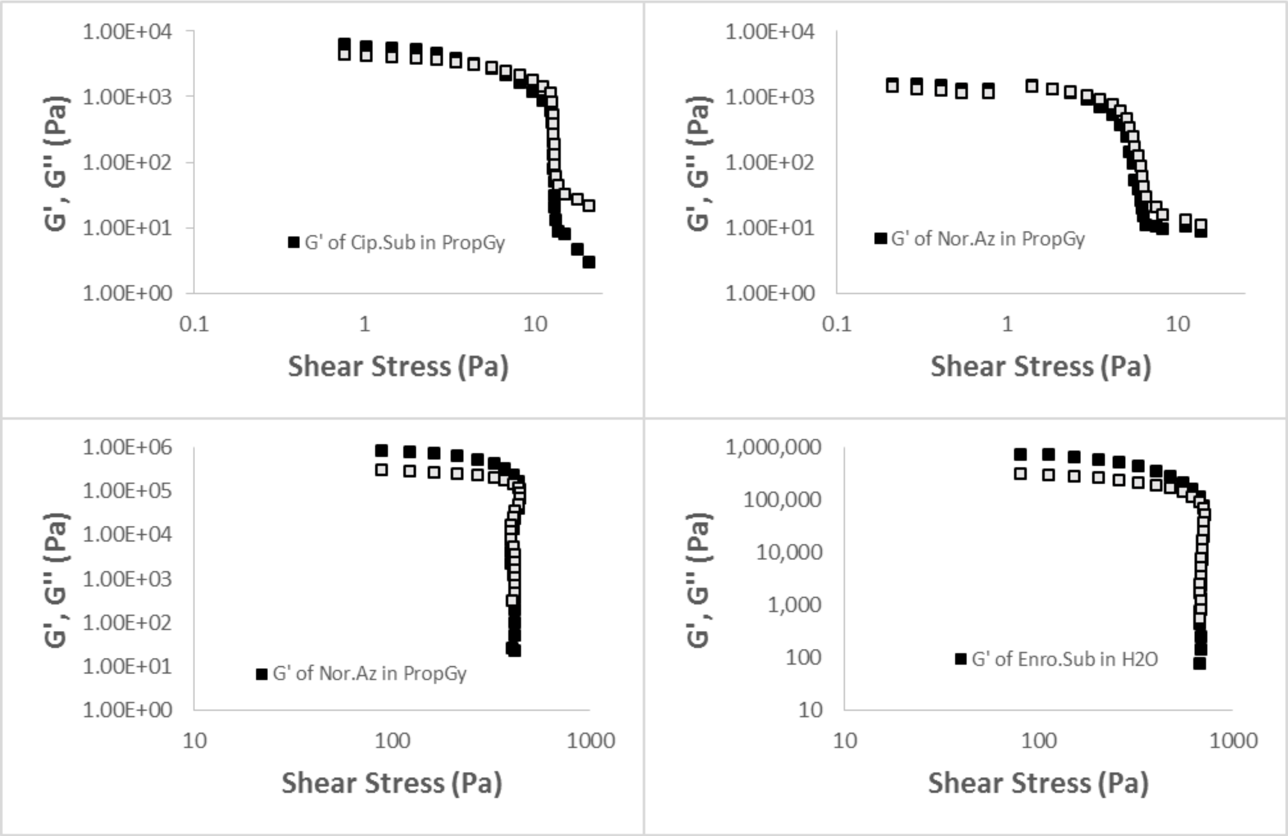

Figure S49. Rheological response (oscillatory amplitude sweep) of supramolecular gels of cip/sub from propylene glycol, nor/az from propylene glycol, enro/sub from propylene glycol and enro/sub from water.

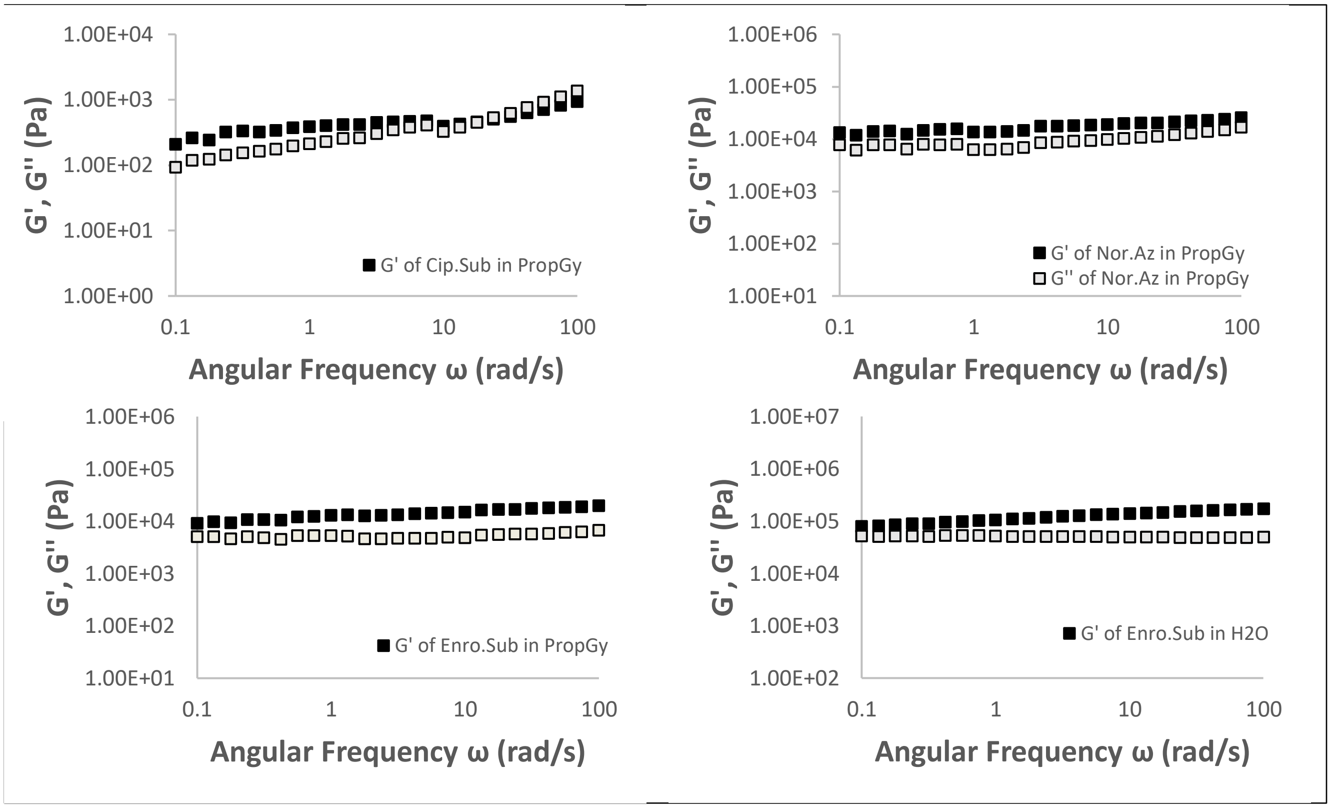

Figure S50. Rheological response (frequency sweep) of supramolecular gels of cip/sub from propylene glycol, nor/az from propylene glycol, enro/sub from propylene glycol and enro/sub from water

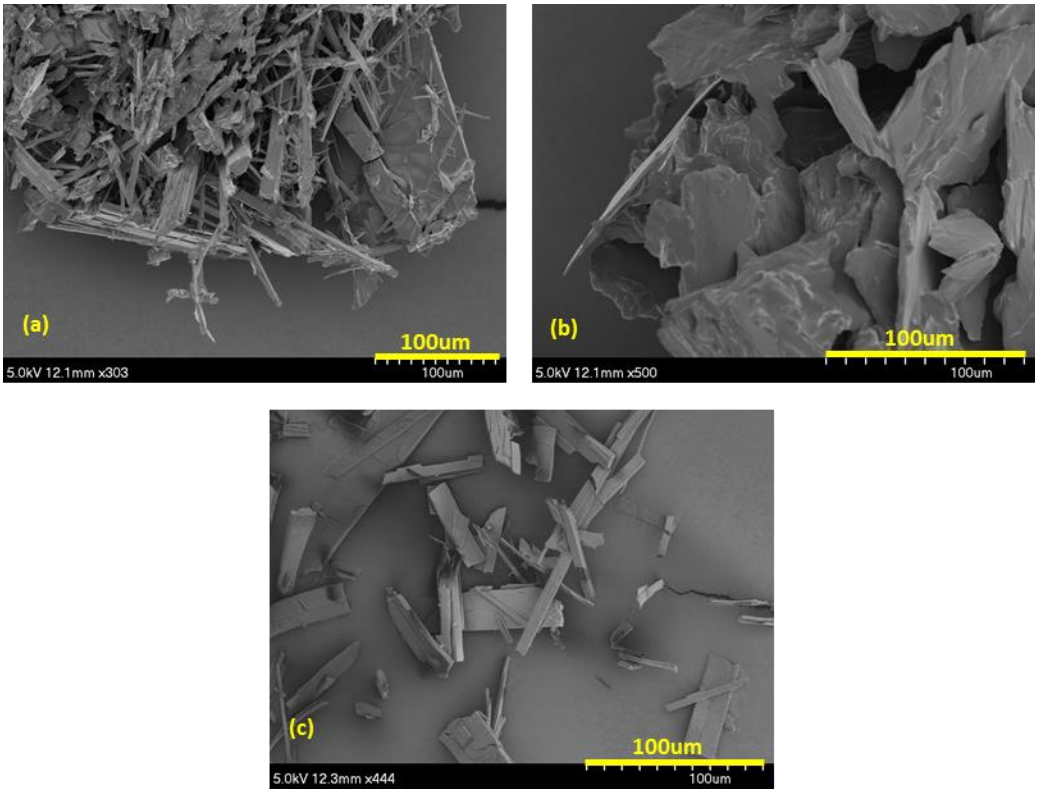

Figure S51. Scanning electron microscopy image of the (a) nor/az/propylene glycol, (b) cip/sub/propylene glycol and (c) enro/sub/water xerogels.

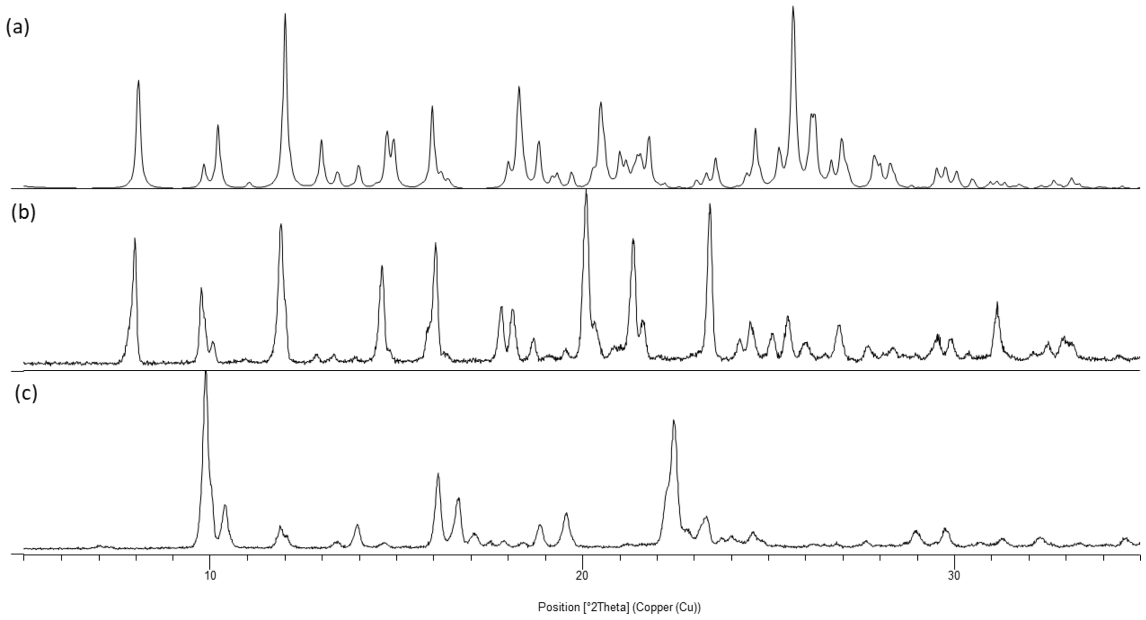

Figure S52. Comparison of the calculated powder pattern of $(enro^{+})(sub^{-})\cdot H_2O$ (a), a gel of enro/sub/water dried under vacuum (b) and a gel of enro/sub/propylene glycol dried under vacuum (c).

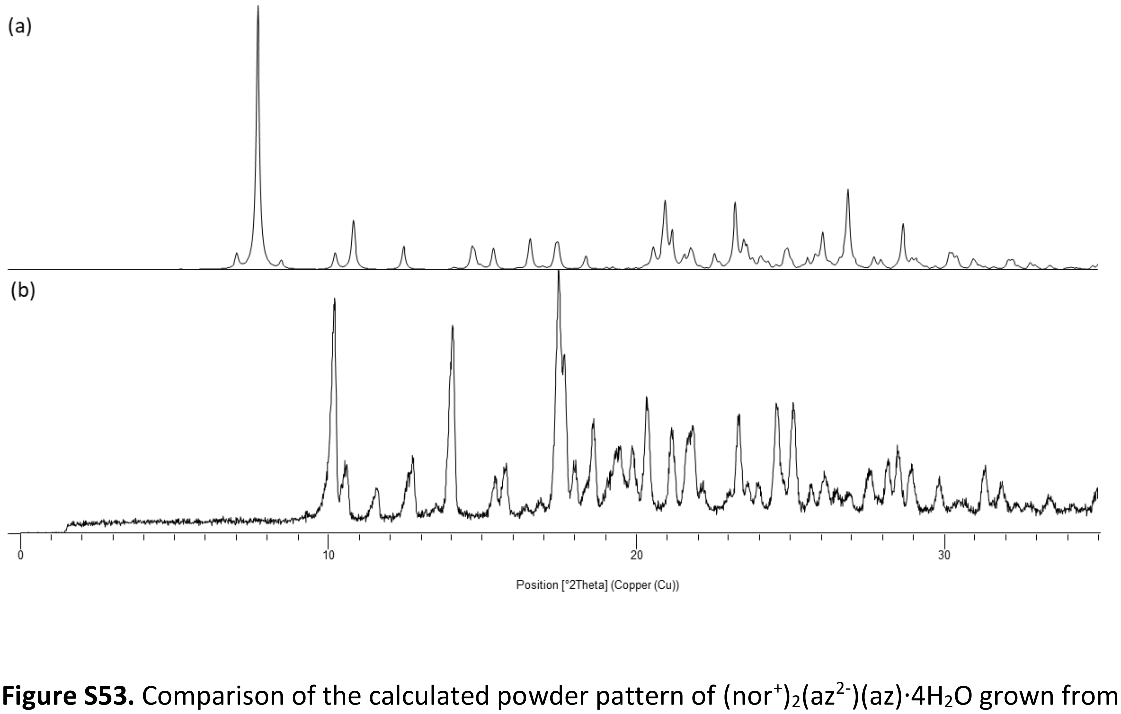

Figure S53. Comparison of the calculated powder pattern of $(nor^{+})_2(az^{2-})(az)\cdot 4H_2O$ grown from methanol (a) and a gel of nor/az/propylene glycol dried under vacuum (b).
